# SAWHALE: A Surrogate-Assisted Self-Adaptive Whale Optimization Algorithm with Novel Asymmetric Opposition-Based Learning for Expensive Optimization Problems

**DOI:** 10.3390/biomimetics11080530

**Published:** 2026-07-31

**Authors:** Oguz Emrah Turgut

**Affiliations:** Department of Industrial Engineering, Engineering and Architecture Faculty, Izmir Bakırçay University, Menemen 35665, Izmir, Turkiye; oguzemrah.turgut@bakircay.edu.tr

**Keywords:** feature selection, fitness inheritance, generalized regression neural networks, surrogate-assisted modeling, oppositional-based learning, whale optimization algorithm

## Abstract

Expensive optimization problems allow for only a small number of exact objective evaluations, and this is where most metaheuristics lose their value. This paper proposes SAWHALE, a surrogate-assisted and self-adaptive whale optimization framework built around two new asymmetric opposition-based learning operators. EDOFAS schedules entropy-driven oppositional probes at several scales, while OPADAMP perturbs the worst coordinates of promising solutions. Surrogate models price the candidates of a global phase and a local phase, and a logistic rule switches between the phases according to the state of the population. Four experiments examine the framework. A component study over nine whale variants at 500 and 1000 dimensions places EDOFAS first, with mean Friedman ranks of 1.650 and 1.575. On the CEC 2014 suite, the full framework attains the best mean rank against five surrogate-assisted optimizers, 2.233 at 30 dimensions and 2.267 at 50, with more Wilcoxon wins than losses against every one of them. An ablation over seven configurations keeps the complete design first at 1.333 and 2.033. On the CEC 2017 suite, the framework ranks first at 1.767 and 1.600 against six metaheuristics, including three newer whale variants, and the cost of the machinery on smooth unimodal ground is reported openly.

## 1. Introduction

High-fidelity simulation and engineering design are now coupled, and in many applications, a single simulation call costs more than the optimization built on it. Printed antenna design [1] and many-objective blast-furnace load optimization [2] illustrate cases in which each candidate evaluation requires a thorough physics-based investigation. Specialized flow-simulation software takes about 18 min to complete a flow analysis of a transonic axial compressor blade described by 33 design variables [3], and a vehicle crashworthiness simulation at Ford Motor Company was reported to take as long as 160 h [4]. Even a small population-based search can require days of computation. These problems, collectively known as “expensive optimization problems,” sharply limit the use of population-based solvers. The budget is the whole problem. Exact function evaluations must be drastically reduced while maintaining solution quality.

Surrogate-assisted evolutionary algorithms (SAEAs) shift part of the evaluation burden onto cheap surrogate models [5]. Recent surveys highlight their growth and the main remaining challenge, including model management, is knowing when a forecast is reliable and when a fine-grained assessment is required [6]. In this context, two economical approximators deserve mention because both are used in the present study and remain very underused, namely the generalized regression neural network (GRNN), an instance-based estimator with a single smoothing parameter [7], and fitness inheritance, which estimates offspring fitness from the weighted fitness of its parents [8].

This work uses the Whale Optimization Algorithm (WHALE) [9] as its host algorithm. WHALE is a swarm optimization technique inspired by the bubble-net foraging behavior of humpback whales and has become one of the most popular metaheuristics over the last decade because of its simple update rules and few parameters. However, this simplicity comes at a cost. The spiral update has a strong exploitative effect that leads to early convergence. The random agent search allows for exploration but lacks direction, and the balance between exploration and exploitation is controlled by a single linearly decreasing factor. To address these weaknesses, improved versions have emerged [10,11,12]. Notable examples include chaotic quasi-opposition with OBL to prevent local optima in engineering design [13], quasi-oppositional learning with a Gaussian bare-bones operator for feature selection and image segmentation [14], quasi-opposition with a nonlinear control factor for cloud task scheduling [15], and refraction-based learning for high-dimensional problems and photovoltaic parameter estimation [16]. These four studies share a single opposing rule, applied consistently throughout the entire duration, without probabilistic scheduling or diversity feedback. By contrast, the WHALE surrogate-assisted line is remarkably sparse. In fact, to the author’s knowledge, it includes a single study, ISAWOA by Wang et al. [17]. This study accelerates parameter identification for fractional-order chaotic systems by employing a local radial basis function surrogate, Lévy flights, and quadratic interpolation, but without an opposition mechanism.

Opposition-Based Learning (OBL) [18] rests on one simple observation. Comparing a candidate with its opposite counterpart and retaining the more favorable one improves our chances of approaching an undiscovered optimum. The canonical reflection x′ = lb + ub − x hits the exact center of the search space, so operators based on it tend to move toward the midpoint of the domain. This clearly illustrates the center-bias phenomenon discovered by Ma et al. [19] in many nature-inspired algorithms, a major disadvantage when the optima are not located at the origin. Each successive refinement affects the geometry of this reflection in a particular way. Quasi-opposition pulls the reflected point back toward the center [20]. Centroid opposition reflects around the population centroid [21]. Dynamic opposite learning scales the reflected solution by a factor that decreases over the course of the iterations [22]. Elite opposition constrains the reflection interval to the bounds derived from the elite solutions [23], and generalized opposition places the reflection point at a random multiple of the currently occupied interval of the population [24]. The reflection lb + s(ub − lb) − x used here places the midpoint between a solution and its reflection at c(s) = [lb + s(ub − lb)]/2, independent of the solution. It equals the domain center only at s = ub/(ub − lb), i.e., at s = 0.5 in the interval [−100,100]. In this paper, it is mentioned that an opposition operator is asymmetric if the reflection center is deliberately displaced from the domain center by a non-random amount. The multiscale array {1.0, 0.5, 0.2} places the reflection center at {+ 50, 0, −30} in the range [−100, 100], keeping the standard reflection as the center probe while moving the two asymmetric probes to either side according to the preset scale. The proposed operators differ from all the above schemes in two respects. The first is cardinality. Each of the opposition-learning schemes [18,20,21,22,23,24] produces exactly one opposite per parent and, therefore, commits to a single displacement, whereas EDOFAS produces one opposite per scale simultaneously, so that a single operation probes several displacements and lets selection decide among them. The second is the control signal. Previous methodologies set the displacement from a geometric statistic of the population, i.e., its centroid [21], bounds determined by elite members [23], the interval it occupies [24], or an iteration schedule [22] or a random selection [20,24]. EDOFAS, in contrast, modifies the scale determined by the Shannon entropy of the population distribution, which measures the population’s occupation of space rather than its location. In OPADAMP, the complementary idea is used in the local phase, where the reflection is based on the elite solutions and is only partially carried out. Therefore, the displacement is limited by the elite geometry and does not go to the full opposite point. This contrast shows that EDOFAS combines multiscale opposition with entropy-based control.

Surrogates and opposition fail in complementary ways. Over-trusted surrogates lead to over-exploitation by pushing the search toward model optima, while over-applied opposition leads to under-exploitation by dispersing the population [25]. Hence, they are logically coupled yet rarely combined. The closest work is the hierarchical RBF-assisted teaching-learning optimizer by Ling [26], where two OBL variants generate antagonistic solutions to diversify globally, but the opposition rule is applied without phase-specific design or feedback control. Thus, the gap in this paper comes down to three shortfalls. The surrogate-assisted WHALE [17] lacks an opposition mechanism. The OBL-improved WHALE variants [13,14,15,16] use a single opposition rule, without probabilistic scheduling or diversity feedback, and surrogates in hierarchical global–local surrogate-assisted evolutionary algorithms are coordinated across search phases without opposition or an information-theoretic switching signal [27,28,29,30]. To the best of the author’s knowledge, no published WHALE paradigm combines phase-specific asymmetric opposition, heterogeneous low-cost surrogates, and closed-loop switching.

The surrogate-assisted self-adaptive WHALE (SAWHALE) proposed in this study addresses this coupling. In the global phase, EDOFAS generates multiscale opposing candidates and evaluates them either exactly during exploration or via fitness inheritance during exploitation, supporting the global search contribution. In the local phase, OPADAMP diversifies elite solutions through partial reflections, while GRNN-guided refinement improves Gaussian perturbation candidates, supporting the local refinement contribution. A stagnation-gated logistic switch determines the phase at each iteration, and entropy- and diversity-based metrics select the operational mode within each phase, thereby supporting the adaptive control contribution. These components collectively reflect the paper’s main contributions. Global search, local refinement, and adaptive control, which include

1. Two asymmetric OBL operators, EDOFAS for exploration and OPADAMP for exploitation, shift their reflection geometry from the domain center under entropy and elite-rank regulation, rather than using a predetermined offset or stochastic selection;
2. The combination of fitness-inheritance and GRNN surrogates in global and local search frameworks with mode selection based on documented exploration and exploitation metrics;
3. A stagnation-aware logistic switch for controlling the two techniques in the traditional WHALE loop;
4. Validation of both operators at the component level, independently of the surrogate machinery, against six classical opposition schemes [18,20,21,22,23,24] on shifted unimodal and multimodal problems at 500 and 1000 dimensions, with performance assessed by the Friedman test with the Iman–Davenport correction and the Nemenyi post hoc procedure;
5. Comparison of five surrogate-assisted optimizers, TLSAPSO [27], ESPSO [31], MSASFS [32], GPEQI [33], and NRO [34], on the CEC 2014 problems at 30 and 50 dimensions, with a uniform limited budget of exact evaluations;
6. A comparative study of six ablated versions of the framework on the CEC problems and under the same evaluation budget, analyzing the effect of opposition, each search stage, the proposed opposition strategies, surrogate guidance, and the switching strategy itself;
7. Evaluation on the CEC 2017 problems at 30 and 50 dimensions against recent general-purpose metaheuristics [35,36,37] and enhanced whale variants [10,11,12], followed by application-level evaluation on twelve constrained engineering designs and eighteen binary feature-selection datasets.

Because this method spans five experimental tiers, the comparison set is deliberately heterogeneous, and each tier plays a distinct role. In the Component tier, two challenger operators are placed in an otherwise unchanged WHALE and face six classical opposition schemes [18,20,21,22,23,24] at dimensions of 500 and 1000. Among these benchmarks, the generalized opposition [24] is the most rigorous; it also changes the reflection point and has been validated on shifted large-scale problems. The surrogate tier is the decisive one and comprises five recent surrogate-assisted optimizers drawn from different model families, including a two-layer PSO with global and local surrogates [27], an evolutionary-sampling PSO with a local RBF [31], a multi-surrogate fractal search [32], a grey-prediction algorithm with a quadratic-interpolation surrogate [33], and an approach based on a neighborhood-regression method [34]. They are implemented under a common evaluation counter, so the comparison reflects the surrogate–opposition design rather than the budget. In the Ablation tier, reduced versions of SAWHALE replace external baselines, so a gain can be attributed to a component rather than to the framework overall. In the Metaheuristic tier, three modern general-purpose optimizers, including the Arithmetic Optimization Algorithm (AOA) [35], the Parrot Optimizer (PARROT) [36], and the Sinh–Cosh Optimizer (SINHCOSH) [37], are compared with the previous three improved whale variants introduced above [10,11,12], distinguishing gains from surrogate–opposition design and whale-family design. In the Application tier, eight optimizers for engineering problems use a uniform constraint-handling rule, and six binary metaheuristics for feature-selection problems use a single transfer function. Table 1 compares the proposed framework with ten relevant studies and highlights its novelty. It is the only framework in this area to assign two dedicated asymmetric opposition operators to different search stages, pair them with low-cost surrogates with global fitness inheritance and local GRNN, and end with entropy-, diversity-, and stagnation-based transitions. Section 6.6 presents an ablation study to test whether the observed benefits come from this combination or from any individual component.

The parts of this work are organized as follows. Section 2 reviews the whale optimization variants that build on OBL and the surrogate-assisted metaheuristics closest to this study. Section 3 presents the basic methods, and Section 4 then develops the two proposed operators together with the global and local search strategies. Section 5 assembles these pieces into the SAWHALE framework and analyses its complexity. The whole experimental campaign lives in Section 6. Section 6.1 validates the two opposition operators on their own at 500 and 1000 dimensions. Section 6.2, Section 6.3, Section 6.4 and Section 6.5 compare SAWHALE with five surrogate-assisted optimizers on the CEC 2014 problems, after which Section 6.6 takes the framework apart in an ablation study on the same problems. Section 6.7 carries the comparison over to the CEC 2017 problems against six recent metaheuristics. Section 6.8 and Section 6.9 close the experiments with applications in constrained engineering design and in feature selection. The conclusions appear in Section 7.

## 2. Related Works

### 2.1. Opposition-Enhanced Whale Optimization Algorithms

OBL has been incorporated into whale-based variants to improve diversity and convergence speed in real-world problems. These combinations differ mainly in the selected opposition rule and in the stage of the run at which it is applied. Chen et al. [13] improved the basic WHALE algorithm by incorporating chaotic random numbers and quasi-oppositional learning to escape local optima during the iterative process and then applied the approach to several engineering design scenarios. Wang et al. [38] combined elite opposition-based learning and a chaotic mechanism with the traditional whale algorithm to optimize the hyperparameters of LSTM networks for forecasting the realized volatility of the CSI 300 index. Dey et al. [39] introduced chaotic opposition earlier in the process, applying it as an oppositional initialization before the standard algorithm begins iterating, and used the resulting COWOA to identify adaptive infinite-impulse-response filter systems. Paul et al. [40] applied an opposition-enhanced whale algorithm to the hydro–thermal–wind–solar scheduling problem with transmission losses. Abd Elaziz and Oliva [41] employed an enhanced opposition-based whale optimizer to determine the diode model parameters of solar cells. Ganguly and Mukherjee [42] introduced a quasi-oppositional whale optimization algorithm for the frequency stabilization of an isolated hybrid power system consisting of a thermal plant, a diesel generator, and a wind turbine.

Together with the three variants already described in Section 1 [14,15,16], these studies give nine opposition-enhanced whale algorithms in total. All nine share one structural choice. Each keeps a single opposition rule that is fixed at design time and used across all executions, either at initialization, per iteration, or both. In none of them does probabilistic scheduling or diversity feedback decide whether the opposition step is worth its evaluation cost.

### 2.2. Surrogate-Assisted Metaheuristic Algorithms

Over the last two decades, many surrogate-assisted metaheuristic algorithms (SAMAs) have been developed for computationally expensive design problems, and they employ various regression techniques. These methods can be grouped by the surrogate model they employ or by the optimization framework they assist. Grouped by surrogate model, Gaussian processes have been used for medium-scale costly problems [43] and for estimating transformer longevity in the presence of model and measurement uncertainties [44]. Other surrogate-model approaches include polynomial regression and Gaussian models within hierarchical structures [45], radial basis functions with particle swarm optimization [46], support vector regression for predicting saltwater intrusion caused by pumping [47], and convolutional networks for modeling indoor airflow patterns [48].

Grouped by optimization method, additional approaches include the following studies. Awad et al. [49] coupled an efficiently adapted surrogate with differential evolution (DE). Cai et al. [50] presented a surrogate-guided DE for high-dimensional problems. Dong and Dong [51] improved the grey wolf optimizer through knowledge extraction based on RBF models. Liu et al. [52] addressed expensive constrained problems with a two-stage surrogate-assisted DE, and Qin et al. [53] integrated different infill criteria into a hybrid model.

Application-driven studies use a similar framework. Chen et al. [54] used Gaussian models, conventional DE, and dimensionality reduction to build surrogates for water-flooding optimization. Fujio and Ogawa [55] used surrogate-assisted evolutionary algorithms to solve a multi-objective optimization problem and to gain insight into axisymmetric scramjet intake design. Shahrokhi and Jahangirian [56] combined a multilayer perceptron with a genetic algorithm to optimize a transonic airfoil model under inviscid and viscous flow conditions. Guo et al. [57] proposed a universal surrogate-assisted differential evolution that uses a radial basis function to approximate the trial population and, thereby, improves local exploitation. They applied the method to a cascaded multi-stage compressor with 126 decision parameters and improved both solution quality and computational efficiency.

A third research line coordinates the surrogate models explicitly, so that global and local searches are unified within hierarchical SAMAs. Sun et al. [27] proposed a two-layer hierarchical surrogate PSO in which global surrogates guide the search and local surrogates improve the fitness evaluation. Chen et al. [28] proposed a hierarchical surrogate-assisted water-cycle algorithm that performs a global search before local refinement. Wang et al. [29] proposed a global-and-local surrogate-assisted differential evolution for expensive constrained optimization with inequality constraints, in which the global phase finds promising regions and the local phase refines them. Chen et al. [30] proposed a two-stage hierarchical surrogate differential evolution for high-dimensional problems that uses RBF-based fitness estimation in the first stage and two local surrogates built on the best solutions in the second stage. This hierarchical line has recently produced four optimizers adopted as comparators in Section 6.2, Section 6.3, Section 6.4 and Section 6.5. These are an evolutionary-sampling PSO with an adaptive Gaussian-kernel RBF trained on personal-best data [31], a multi-surrogate stochastic fractal search with a cubic RBF and a Gaussian process under expected-improvement criteria [32], a grey-prediction evolutionary algorithm with a quadratic-interpolation surrogate [33], and a neighborhood-regression method that predicts a descent direction using local linear models [34]. The experimental configurations and the reported results are summarized in Table 2.

### 2.3. Past Literature on OBL-Integrated Surrogate Models

The combination of OBL and surrogate models is still rare, but recent studies have shown that it can improve optimization performance, especially in high-dimensional settings. For example, Ling [26] proposed a hybrid surrogate-assisted framework that uses an RBF approximation and a teaching–learning-based optimizer with two OBL variants. In this framework, contrasting solutions support global exploration and improve the training data for the local surrogates. ISAWOA [17] approaches the intersection from the surrogate side by integrating a local cubic RBF into the whale algorithm to pre-screen candidates generated by Lévy flights and quadratic interpolation. It does not include any mechanism for opposition. Thus, the two research lines have come close to each other without meeting within a whale framework. These hybrid methods mitigate known surrogate limitations by generating more diverse training solutions, delaying early convergence, and reducing the need for exact evaluations. Taken together, the literature presents various methodologies relevant to current research, but a common principle emerges. Opposition-generated diversity can improve the accuracy and robustness of surrogate models, and it thereby enhances optimizer performance in expensive black-box scenarios.

### 2.4. Comparative Summary

Table 2 summarizes the experimental parameters and reported results for the four surrogate-assisted comparators from Section 6.2, Section 6.3, Section 6.4 and Section 6.5 and the two studies most closely aligned with the current research. Read column-wise, the table makes the three shortfalls of Section 1 concrete. The opposition column is empty for all surrogate-assisted entries, including the only whale representative [17], while the surrogate column is empty for the strongest opposition scheme [24], whose generalized rule, despite displacing the reflection point, performed poorly precisely on shifted and large-scale problems. None of the nine opposition-enhanced whale algorithms in Section 2.1 adaptively schedule their single rule.

## 3. Background Methods

Four well-known components underlie the framework proposed in this work. These are the Whale Optimization Algorithm, which performs the search, the canonical opposition rule, which the operators of Section 4 refine, and two low-cost approximators that estimate the quality of candidate solutions. Each component is presented briefly below, only to the level required by later developments. Detailed derivations and a broader discussion remain with the original sources.

### 3.1. Whale Optimization Algorithm

The whale optimization algorithm (WHALE) [9] is a collective approach inspired by the bubble-net feeding method of humpback whales. It relies on a concise set of rules governed by a single diminishing coefficient. Positions change in three ways. In the circumferential mechanism, each agent updates its position according to:

(1) $$D=\left|\;C\;X_{best}-X\left(t\right)\;\right|$$

(2) $$X\left(t+1\right)=X_{best}-M\;D$$

(3) M=2kr−k

(4) C=2r

where $X_{best}$ denotes the best solution found so far and $X\left(t\right)$ the current position. The factor r is a uniformly random number in [0,1], sampled independently at each occurrence, and k decreases linearly from 2 to 0 during the process. In this work, k and the other schedules are driven by the fraction of the evaluation budget spent rather than by the iteration count, so they are tied directly to budget consumption and end exactly when the budget runs out. The bubble-net process surrounds the best solution along a logarithmic spiral, as given by Equation (2), and the choice between the two mechanisms is made with equal probability.

(5) $$X\left(t+1\right)=\left\{\begin{array}{ll} X_{best}-M\;D, & p<0.5\\ \left|X_{best}-X\left(t\right)\right|\;e^{\;bl}cos\left(2\pi l\right)+X_{best}, & p\geq 0.5 \end{array}\right.$$

where b=1 controls the spiral shape, while $p∼U\left(0,1\right)$ and $l∼U\left(-1,1\right)$. Whenever $\left|M\right|\geq 1$ holds, the exploration mechanism replaces the best solution with a randomly chosen agent:

(6) $$X\left(t+1\right)=X_{rand}-M\left|\;C\;X_{rand}-X\left(t\right)\;\right|$$

steering agents away from the current best solution while preserving the global search. The simplicity of this framework carries costs. The spiral described in Equation (5) imposes a powerful exploitative force. The random-agent maneuver in Equation (6) offers undirected exploration, and the balance between these elements relies solely on the coefficient k. The operators of Section 4 are designed to remove exactly these deficiencies.

### 3.2. Opposition-Based Learning

Opposition-based learning (OBL) [18] evaluates, alongside a candidate x, its opposite point:

(7) $${\overset{˘}{x}}_{j}=lb_{j}+ub_{j}-x_{j},\;\;j=1,\ldots ,D$$

and keeps the fitter of the two. Equation (7)’s reflection passes through the center of the search box, while the refinements reviewed in Section 1 and Section 2 [20,21,22,23,24] change, resize, or randomize this reflection in several ways. Section 4.1 describes the geometric property that the proposed operators generalize and the way in which they avoid the center.

### 3.3. Fitness Inheritance Surrogates

Fitness inheritance [8] estimates the objective value of an unexamined candidate from the known fitness of previously evaluated solutions. The approach works by similarity over stored instances, and no training is involved. The approximator is nothing more than the catalog of accurately assessed points. Two practical rules follow from this idea [8]. The average rule assigns to an offspring the average fitness of its two parents.

(8) $$\hat{f}\left(x_{h}\right)=1/2\left(f\left(x_{p1}\right)+f\left(x_{p2}\right)\right)$$

while the weighted rule generalizes to M reference solutions:

(9) $$\hat{f}\left(x_{h}\right)=\left\{\begin{array}{ll} f\left(x_{i}\right), & \mathrm{dist}\left(x_{h},x_{i}\right)=0\\ \frac{\sum_{i=1}^{M}w_{i}\;f\left(x_{i}\right)}{\sum_{i=1}^{M}w_{i}}, & \mathrm{otherwise} \end{array}\right.$$

(10) $$w_{i}=1-\frac{dist\left(x_{h},x_{i}\right)}{dist\left(x_{U},x_{L}\right)}$$

where $dist\left(\cdot ,\cdot \right)\;$denotes the Euclidean distance while $x_{U},x_{L}$ mark the search box corners. In this way, $w_{i}\in \left[0,1\right]$ measures normalized proximity. This estimator, in its multi-reference, similarity-weighted form, is consistent with the fitness-estimation approaches defined for swarm optimizers [58]. Fitness inheritance has also been applied in noisy, multi-objective, and practical settings [59,60]. The implementation used in the proposed global search, the parameter M, and the choice of reference solutions are presented in Section 4.2.4.

### 3.4. Generalized Regression Neural Networks

The generalized regression neural network (GRNN) [61] is a nonparametric kernel regressor governed by a single smoothing parameter. Given an archive $\left\{\left(x_{i},f\left(x_{i}\right)\right)\right\}$, the estimate at a query point x is the kernel-weighted average:

(11) $$\hat{f}\left(x\right)=\frac{\sum_{i}K_{i}\left(x\right)\;f\left(x_{i}\right)}{\sum_{i}K_{i}\left(x\right)}$$

(12) $$K_{i}\left(x\right)=exp\left(-\frac{d_{i}^{\;2}\left(x\right)}{2\sigma^{2}}\right)$$

where $d_{i}\left(x\right)$ denotes the distance from x to the archive point $x_{i}$, and σ stands for the smoothing bandwidth. Like fitness inheritance, GRNN is a lazy learner. There is no training phase. The archive itself is the model, and the prediction cost grows with the archive size. Recursive and adaptive versions have appeared in recent work [7]. GRNN surrogates have also served many engineering applications [62]. The proposed system, therefore, constrains Equation (11) to the k-nearest archive sites for each query and updates the archive in real time.

## 4. Proposed Opposition-Based Learning Search Schemes

This section develops the two opposition operators contributed by this study and the global and local search schemes built around them. The notation is collected in Table 3.

### 4.1. Design Rationale for Controlled Asymmetric Reflection

Both operators are instances of the scaled reflection ${x^{\prime }}_{j}=lb_{j}+s\;\left(ub_{j}-lb_{j}\right)-x_{j}$, whose midpoint between a solution and its image is:

(13) $$c\left(s\right)=\frac{lb+s\;\left(ub-lb\right)}{2}$$

The reflection center is independent of the solution and coincides with the domain center only at $s^{*}=ub/\left(ub-lb\right)$. On the symmetric domain [−100,100], $s^{*}=0.5$. Throughout this work, “asymmetric” describes an opposition operator whose reflection center is deliberately displaced from the domain center by a non-random amount. The base scale set chosen here $S_{base}=\{0.2,0.5,1.0\}$ has reflection centers at {−30, 0, +50} on [−100,100] at a unit scale factor. The canonical reflection is the middle probe, and the two asymmetric probes sit on either side of it. As the population spread varies, the entropy modulation expands or shrinks the set around those centers.

Two structural properties separate the proposed operators from standard methods. First, they treat cardinality as a design element. Traditional models are limited to one opposite per parent per application, whereas EDOFAS creates one opposite for every scale at once and allows selection to negotiate among them. Since $S_{base}\;$includes $s^{*}\;$itself, the probe set contains, by construction, the classical antithesis of Equation (7). After scoring the probes and keeping the best, the selected candidate is no less effective than the classical alternative, at the managed cost of the extra probes. The second property concerns the control signal. Previous approaches determine displacement using a geometric statistic of the population, a sequence of iterations, or a random selection, whereas EDOFAS calibrates its scale based on the Shannon entropy of the population’s fitness distribution. This is a measure of how the population interacts with the objective landscape rather than of its location in the parameter space. OPADAMP, by contrast, leans toward exploitation, since its reflection is anchored on elite solutions and is only partial. Its displacement is confined by elite geometry and never reaches the exact opposite point.

### 4.2. Entropy-Driven Opposition with Fitness-Adaptive Sampling (EDOFAS)

EDOFAS is the global phase operator. It assesses how the evolving population fills the target landscape, uses that assessment to decide how competing candidates are priced and at which scales they are created, concentrates opposition where it is most informative, and contracts the augmented population back to size N through fitness with a density tiebreak. The subsections that follow trace these phases in the execution order of Algorithm 1.
**Algorithm 1.** EDOFAS global search**Input:** population X, fitness F, archive A, incumbent $\left(x^{*},f^{*}\right)$, budget state $\left(FES,FES_{max}\right)$
**Output:** updated X, F, A, $\left(x^{*},f^{*}\right)$, FES 1. compute $H_{n}$ from the population fitness distribution by Equations (14)–(18) 2. compute $r_{imp}$ by Equation (19) and the scores $E_{expl}$, $E_{expt}$ by Equations (20) and (21) 3. build the middle-ranked band $I_{mid}$ by Equation (26); $M\leftarrow |I_{mid}|$ 4. compute the scale set $\{s_{k}\left(t\right){\}}_{k=1}^{K}$ by Equation (23) 5. **for each** $i\in I_{mid}$ **do** 6.  **for**
k=1 **to**
K **do** 7.   generate the opposite ${x^{\prime }}_{i,k}$ by Equations (24) and (25) 8.  **end for** 9. **end for** 10. **if**
$E_{expl}>E_{expt}+\gamma$ **then** ▹ exploration dominates, Equation (22) 11.  **for each** opposite $x^{\prime }$ **while**
$FES<FES_{max}$ **do** 12.   evaluate $f\left(x^{\prime }\right)$ exactly; FES←FES+1; append $\left(x^{\prime },f\left(x^{\prime }\right)\right)$ to A; update $\left(x^{*},f^{*}\right)$ 13.  **end for** 14. **else** ▹ exploitation dominates 15.  price every opposite by Equations (9) and (10) with M=4 references drawn uniformly at random from X 16. **end if** 17. form the combined set of the N parents and the MK opposites 18. select the N survivors by the density-tiebroken lexicographic order of Equations (27) and (28) 19. **if** the inheritance mode was used **then** 20.  **for each** surviving new position x **while**
$FES<FES_{max}$ **do** 21.   evaluate $f\left(x\right)$ exactly; FES←FES+1; append to A; update $\left(x^{*},f^{*}\right)$ 22.  **end for** ▹ no inherited price persists in any record 23. **end if** 24. truncate A to its newest 5N entries (FIFO) 25. **return** X, F, A, $\left(x^{*},f^{*}\right)$, FES

#### 4.2.1. Population Entropy

The fitness values are first normalized to $\left[0,1\right]$:

(14) $$f_{i}^{norm}\left(t\right)=\frac{f\left(x_{i}\right)-\underset{j}{min}f\left(x_{j}\right)}{\underset{j}{max}f\left(x_{j}\right)-\underset{j}{min}f\left(x_{j}\right)+\epsilon },\;\;i=1,\ldots ,N$$

with ϵ being a small constant that prevents division by zero. The unit interval is partitioned into $n_{bins}=10$ equal bins. Each individual is assigned to one, and the bin probabilities follow:

(15) $$b_{i}=min\left(n_{bins},\;\lfloor f_{i}^{norm}\;n_{bins}\rfloor +1\right)$$

(16) $$\;p_{b}=\frac{count_{b}}{\sum_{b^{\prime }=1}^{n_{bins}}count_{b^{\prime }}},\;\;b=1,\ldots ,n_{bins}$$

where $count_{b}$ counts the individuals that Equation (15) assigns to bin b. The Shannon entropy [63] of this distribution and its normalized form are:

(17) $$H\left(t\right)=-\sum_{b:\;p_{b}>0}p_{b}lnp_{b}$$

(18) $$H_{n}\left(t\right)=\frac{H\left(t\right)}{ln(n_{bins})}$$

Here, $H_{n}\approx 0$ indicates a fitness-concentrated population, while $H_{n}\approx 1$ marks a fitness-diverse population. Since the empty bins do not carry any component and are not included in the sum, Equation (16) yields a proper probability distribution.

#### 4.2.2. Mode Selection by Exploration and Exploitation Scores

Whether the oppositional candidates are scored by exact evaluations or evaluated by inheritance is decided by two recorded scores. For the relative improvement rate:

(19) $$r_{imp}\left(t\right)=min\left(\frac{\left|f_{best}\left(t-1\right)-f_{best}\left(t\right)\right|}{|f_{best}\left(t-1\right)|+\epsilon },\;1\right)$$

the scores read:

(20) $$E_{expl}\left(t\right)=H_{n}\left(t\right)\;\left(1-\hat{t}\;\right)$$

(21) $$E_{expt}\left(t\right)=\left(1-H_{n}\left(t\right)\right)\;\hat{t}\;\left(1-r_{imp}\left(t\right)\right)$$

and exact scoring is selected when exploration dominates by the margin γ=0.5:

(22) $$E_{expl}\left(t\right)>E_{expt}\left(t\right)+\gamma$$

Equation (22) typically holds at the start of the process, when the fitness diversity is large, the budget is largely unused, and the opposites are evaluated exactly. As diversity shrinks and progress increases, the imbalance collapses, and evaluation shifts toward fitness inheritance. The margin γ deliberately keeps the mode stable when the budget runs low, saving exact evaluations for the stage at which contradictory information matters most.

#### 4.2.3. Multiscale Opposite Generation

The scale set responds jointly to budget progress and to the entropy of Equation (18):

(23) $$s_{k}\left(t\right)={\bar{s}}_{k}\;\left(1-\hat{t}\;\right)\left(1+\lambda \;\left(1-H_{n}\left(t\right)\right)\right),\;\;{\bar{s}}_{k}\in S_{base},\;k=1,\ldots ,K$$

where K = 3 and λ = 1.$S_{base}=\left\{0.2,0.5,1.0\right\}.\;$The probes begin at maximum width, contract as the budget is depleted, and then re-broaden when the fitness distribution collapses ($H_{n}\to 0$). A decline in diversity thus translates into a wider opposite move, with $1+\lambda \;\left(1-H_{n}\left(t\right)\right).\;$For each selected parent, one opposite is created for each scale within the box constraints.

(24) $${x^{\prime }}_{i,k,j}=lb_{j}+s_{k}\left(t\right)\;\left(ub_{j}-lb_{j}\right)-x_{i,j}$$

(25) $${x^{\prime }}_{i,k,j}\leftarrow max\left(lb_{j},\;min\left(ub_{j},\;{x^{\prime }}_{i,k,j}\right)\right)$$

#### 4.2.4. Fitness-Adaptive Sampling and Candidate Pricing

**Figure 1:**
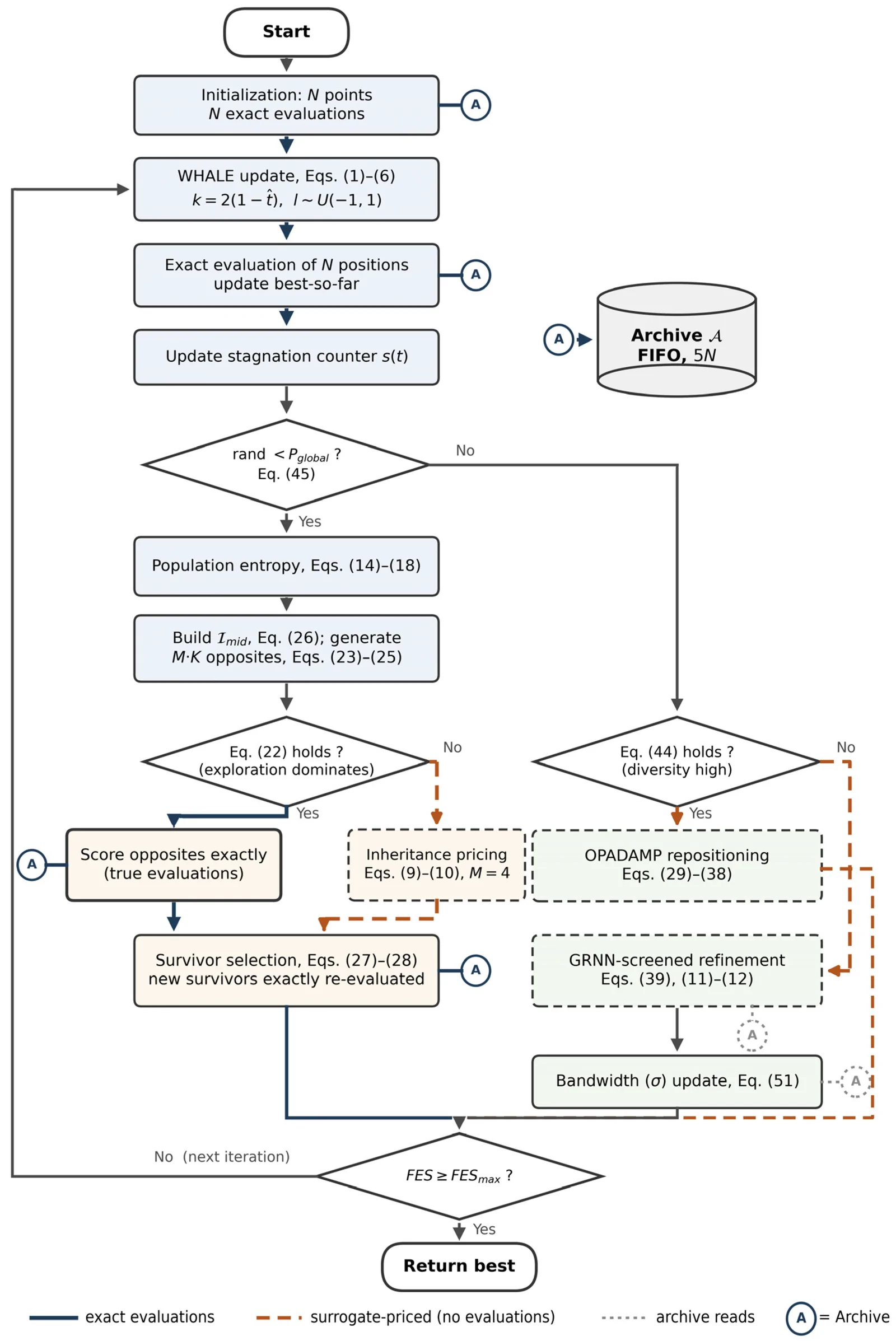
Flowchart of SAWHALE.

Opposition is not applied uniformly. The parents selected for it are drawn from the middle-ranked band:

(26) $$I_{mid}=\left\{\pi \left(round\left(0.25N\right)+1\right),\;\ldots ,\;\pi \left(round\left(\left(0.25+\rho_{sel}\right)N\right)\right)\right\},\;\;\rho_{sel}=0.5$$

where π denotes the fitness-sorted permutation. The opposition budget targets the middle band. Its members are established enough to transmit information about the landscape, yet distant enough from the incumbent region that their reflections probe genuinely novel territory. Elites are left intact because their refinement is a local phase. In the exact mode, the objective function evaluates each of the MK opposites (M = |$I_{mid}$|) and adds it to the archive. In contrast, the inheritance-mode evaluation scheme prices each opposite according to Equations (9) and (10), with M = 4 reference solutions chosen uniformly at random, with replacement, from the current population. A candidate that coincides with a reference directly inherits that reference’s fitness. Reflected candidates have no parents in the recombinative sense, so random references are the appropriate choice here. Four references average individual reference noise while bounding the pricing cost. The framework’s default pricing mode is inheritance by design. As a result, most oppositional candidates over a run are evaluated by Equations (9) and (10) at a cost of O(4MKD) floating-point operations and zero exact evaluations, which is the dashed path of Figure 1. The margin γ of Equation (22) then limits precise scoring to the early, exploration-dominated phase. The K-fold cardinality in Section 4.1 multiplies the number of candidates, and inheritance keeps that multiplication in arithmetic rather than in simulation calls. Multiscale opposition, therefore, stays affordable under a tightly rationed budget.

#### 4.2.5. Diversity-Driven Survivor Selection

Parents and opposites compete together. A replacement subsample of size $n_{comb}=N+MK$ is chosen uniformly. That is, a subsample of half the combined size, and each combined member is assigned a density score based on the distance to its nearest subsample member.

(27) $$d_{i}^{min}=\underset{x_{s}\in Sub}{min}\;∥x_{i}-x_{s}∥$$

where Sub stands for the subsample. Survivors are the N smallest under the lexicographic order of the key:

(28) $$key\left(i\right)=\left(f_{i},\;-d_{i}^{min}\right)$$

The key follows the fitness ranking and breaks ties in favor of isolated individuals, thus preserving the spatial spread of the surviving population. In the inheritance mode, only inherited individuals contribute to this ranking. Each new surviving position is then promptly evaluated exactly, which ensures that no estimated value remains in the population, archive, or best-so-far record (Section 5.3).

### 4.3. Adaptive-Amplification-Based Oppositional Learning (OPADAMP)

The OPADAMP operator adds diversity to the local phase. It implements a partial reflection for the elite half of the population about the elite centroid. The amplitude varies with each elite’s quality and recent movement, according to a sinusoidally weighted schedule. No objective evaluations are spent by the operator. It only repositions the solutions, which are then reviewed in the main sweep of the next iteration.

The displacement record of the population and the elite set are given by:

(29) $$\delta_{i}=x_{i}\left(t\right)-x_{i}\left(t-1\right)$$

(30) $$∥\delta {∥}_{max}=\underset{i}{max}\;∥\delta_{i}∥$$

(31) $$E=\{\pi \left(1\right),\ldots ,\pi \left(k_{elite}\right)\},\;\;k_{elite}=N/2$$

The amplitude schedule is:

(32) $$s_{t}=\varphi \;\left(1+sin\left(\pi \hat{t}/2\right)\right)\left(1-\hat{t}\;\right)\left(1+0.2u\right),\;\;\;u∼U\left(0,1\right),\;\varphi =0.5$$

The ascending sinusoidal component and the diminishing linear factor concentrate the operator’s power in the middle of the run. At the beginning ($\hat{t}$ ≈ 0), the elite centroid carries little information, and the amplitude is moderate. At $\hat{t}$ ≈ 0.3, both factors reach their maximum, so the most important diversification occurs precisely when elites are established, while convergence is still incomplete. As time progresses, the factor 1 − $\hat{t}$ reduces the amplitude to zero, which protects the final refinement. A uniform linear decline would place its maximum amplitude at a non-informative centroid at $\hat{t}$ = 0, whereas an exponential decay would eliminate diversification before the onset of mid-run stagnation. The sinusoidal–linear combination is the simplest schedule with an internal maximum. The multiplicative jitter 1 + 0.2u desynchronizes the elites, preventing them from probing at the same radius. The weights used for each elite link the magnitude to the solution’s quality and mobility.

(33) $$w_{i}=\frac{f_{max}-f\left(x_{i}\right)}{f_{max}-f_{min}+\epsilon }$$

(34) $$u_{i}=\frac{∥\delta {∥}_{max}}{∥\delta_{i}∥+∥\delta {∥}_{max}}$$

(35) $$\;z_{i}=w_{i}\;u_{i}$$

directing the largest displacements to the best elites ($w_{i}\to 1$) and the stalled ones ($u_{i}\to 1$). That is, the individuals for which diversification is most valuable, while recently mobile or lower-ranked elites are perturbed gently. The reflection itself is anchored at the elite centroid:

(36) $${\overset{¯}{x}}_{E}=\frac{1}{k_{elite}}\sum_{i\in E}x_{i}$$

(37) $$x_{i,j}^{opp}=x_{i,j}+2\;z_{i}\;s_{t}\;\left({\bar{x}}_{E,j}-x_{i,j}\right),\;\;i\in E$$

(38) $$x_{i,j}^{opp}\leftarrow max\left(lb_{j},\;min\left(ub_{j},\;x_{i,j}^{opp}\right)\right)$$

Equation (37) represents a partial reflection by ${\overset{¯}{x}}_{E}$. When $2z_{i}s_{t}=1$, the elite is exactly the centroid. When $2z_{i}s_{t}=2$, it is the full mirror image. For the amplitudes of Equations (32)–(35), the shift is a limited step toward or slightly over the centroid. The mapping’s fixed point is the elite centroid itself, confining the displacement to the elite geometry, in contrast to centroid opposition [21], which mirrors every population member around the overall centroid without amplitude control, and to the domain-centered rule of Equation (7), which pulls to the box center. The non-elite members keep their positions.

### 4.4. GRNN-Screened Local Refinement

The refinement mode of the local phase perturbs each elite and lets a k-nearest-neighbor GRNN choose the most promising perturbation. For each i∈E, $n_{cand}=8$ candidates are drawn:

(39) $$x_{m}^{c}=x_{i}+\beta \;\left(ub-lb\right)⊙\xi_{m},\;\;\;\xi_{m}∼N\left(0,I_{D}\right),\;\beta =0.05,\;m=1,\ldots ,n_{cand}$$

Each candidate is clipped to the box and then evaluated using Equations (11) and (12) against its $k_{pred}=20$ nearest archive sites in the current bandwidth σ. The candidate with the lowest fitness value then replaces the elite at that position, and no predicted fitness is carried over $x_{i}\;$The moved elite is evaluated at the start of the next iteration before it can affect selection, the archive, or the best-so-far record. The GRNN ranks eight local options, so an incorrectly evaluated candidate can replace at most one elite in a single iteration.

### 4.5. Local Mode Selection

Between diversification (Section 4.3) and refinement (Section 4.4), the local phase chooses based on spatial diversity. With population diversity:

(40) $$Div_{pop}\left(t\right)=\frac{1}{D}\sum_{j=1}^{D}std\left(x_{1,j},\ldots ,x_{N,j}\right)$$

normalized by the running maximum $Div_{max}$ maintained in Equation (47):

(41) $$nDiv\left(t\right)=min\left(\frac{Div_{pop}\left(t\right)}{Div_{max}+\epsilon },\;1\right)$$

the local scores mirror the global construction:

(42) $$E_{expl}^{loc}\left(t\right)=nDiv\left(t\right)\;\left(1-\hat{t}\;\right)$$

(43) $$E_{expt}^{loc}\left(t\right)=\left(1-nDiv\left(t\right)\right)\;\hat{t}\;\left(1-r_{imp}\left(t\right)\right)$$

and OPADAMP runs when:

(44) $$E_{expl}^{loc}\left(t\right)>E_{expt}^{loc}\left(t\right)+\gamma$$

with the same margin γ = 0.5. Otherwise, the GRNN refinement is run, followed by the bandwidth update. Thus, diversification is scheduled while the population is spatially distributed relative to its own history. And refinement takes over as the search contracts.

## 5. The SAWHALE Framework

### 5.1. Architecture and Information Flow

SAWHALE joins the operators of Section 4 to the WHALE loop of Section 3.1. Each cycle first runs the standard WHALE update over the entire population and evaluates the moved population exactly. The results enter the archive, and a stagnation-gated logistic switch then chooses the second-stage scheme, which is either the global scheme (Algorithm 1) or the local scheme (Algorithm 2). Every schedule is driven by the budget progress $\hat{t}=FES/FES_{max}$. The scheduled quantities are the WHALE factor k, the opposition scales of Equation (23), and the OPADAMP amplitude of Equation (32). Every schedule, therefore, ends precisely when the budget does, regardless of how the evaluations are distributed across the stages. The full algorithm flow, with exact evaluation, free surrogate evaluation, and archive access, is shown in Figure 1. 

**Algorithm 2.** Local search phase with OPADAMP diversification or GRNN-screened refinement**Input:** X$,\;X_{prev}$, F$,\;F_{prev}$, archive A, bandwidth σ$,\;Div_{max}$$,\;\mathrm{budget}\;\mathrm{state}\;\left(FES,FES_{max}\right)$ **Output:** repositioned X, updated σ$\;\mathrm{and}\;Div_{max}$ 1. $\mathrm{compute}\;Div_{pop}$ by Equation (40) and nDiv by Equation (41) 2. $\mathrm{compute}\;r_{imp}$$\;\mathrm{by}\;\mathrm{Equation}\;(19)\;\mathrm{and}\;\mathrm{the}\;\mathrm{scores}\;E_{expl}^{loc}$$,\;E_{expt}^{loc}$ by Equations (42)–(43) 3. build the elite set E$\;\mathrm{of}\;\mathrm{the}\;\mathrm{best}\;k_{elite}=N/2$ individuals by Equation (31) 4. $\mathrm{if}\;E_{expl}^{loc}>E_{expt}^{loc}+\gamma$ **then** ▹ diversification, Equation (44) 5.  $\mathrm{compute}\;\delta_{i}$$\;\mathrm{and}\;∥\delta {∥}_{max}$$\;\mathrm{from}\;X-X_{prev}$ by Equations (29)–(30) 6.  $\mathrm{compute}\;\mathrm{the}\;\mathrm{amplitude}\;s_{t}$$\;\mathrm{by}\;\mathrm{Equation}\;(32)\;\mathrm{and}\;\mathrm{the}\;\mathrm{elite}\;\mathrm{centroid}\;{\overset{¯}{x}}_{E}$ by Equation (36) 7.  for each i∈E **do** 8.    $\mathrm{compute}\;\mathrm{the}\;\mathrm{weights}\;w_{i}$$,\;u_{i}$$,\;z_{i}$ by Equations (33)–(35) 9.    $\mathrm{reposition}\;x_{i}$$\;\mathrm{by}\;\mathrm{the}\;\mathrm{partial}\;\mathrm{reflection}\;\mathrm{through}\;{\overset{¯}{x}}_{E}$, Equations (37)–(38) 10.  **end for** 11. **else** ▹ refinement 12.  **if**
$|A|>k_{pred}$
**then** 13.    for each i∈E **do** 14.     $\mathrm{draw}\;n_{cand}$ Gaussian candidates by Equation (39), clipped to the box 15.     $\mathrm{predict}\;\mathrm{each}\;\mathrm{candidate}\;\mathrm{by}\;\mathrm{Equations}\;(11)–(12)\;\mathrm{restricted}\;\mathrm{to}\;\mathrm{its}\;k_{pred}$ nearest archive points 16.     $x_{i}\leftarrow$ the candidate with the smallest prediction ▹ position only; no fitness assigned 17.    **end for** 18.  **end if** 19.  $\mathrm{update}\;Div_{arch}$$,\;Div_{max}$, SCI$,\;\tau_{t}$, and σ by Equations (46)–(51) 20. **end if** 21. **return**
X, σ$,\;Div_{max}$

Algorithm 3 also provides the full workload of the proposed SAWHALE optimizer and subsequently explains each algorithm step in the correct order.


**Algorithm 3.** SAWHALE main loop**Input:** objective f, population size N$,\;\mathrm{bounds}\;\left[lb,ub\right]$$,\;\mathrm{budget}\;FES_{max}$ **Output:**$\;\mathrm{best}\;\mathrm{solution}\;x^{*}$$\;\mathrm{and}\;\mathrm{its}\;\mathrm{objective}\;\mathrm{value}\;f^{*}$ 1. initialize X with N uniform points; evaluate each exactly; FES←N; fill A 2. $\left(x^{*},f^{*}\right)\leftarrow$ the best initial individual; stagnation counter s←0 3. **while**
$FES<FES_{max}$ **do** 4.  $\hat{t}\leftarrow FES/FES_{max}$$;\;k\leftarrow 2\left(1-\hat{t}\right)$ 5.  $\mathrm{record}\;X_{prev}\leftarrow X$$,\;F_{prev}\leftarrow F$ 6.  **for**
i=1 to N$\;\mathrm{do}\;\mathrm{update}\;x_{i}$$\;\mathrm{by}\;\mathrm{the}\;\mathrm{WHALE}\;\mathrm{rules}\;\mathrm{of}\;\mathrm{Equations}\;(1)–(6)\;\mathrm{with}\;l∼U\left(-1,1\right)$ **end for** 7.  **for** i=1 **to** N **while** $FES<FES_{max}$ **do** 8.    $\mathrm{evaluate}\;f\left(x_{i}\right)$ exactly; FES←FES+1; append to A (FIFO, 5N$);\;\mathrm{update}\;\left(x^{*},f^{*}\right)$ 9.  **end for** 10.  s←0$\;\mathrm{if}\;f^{*}$ improved this iteration, **else**
s←s+1 11.  $\mathrm{compute}\;P_{global}$ from s by Equation (45) 12.  **if**
$\mathrm{rand}\;<P_{global}$ **then** run Algorithm 1 ▹ global scheme 13.  **else** run Algorithm 2 ▹ local scheme 14.  **end if** 15. **end while** 16. **return**
$\left(x^{*},f^{*}\right)$


### 5.2. The Stagnation-Gated Logistic Switch

The switch converts the integer stagnation counter $s\left(t\right)$ into the probability of running the global scheme. The counter records the number of consecutive iterations without improvement of the best-so-far value and returns to zero whenever an improvement occurs. The probability reads:

(45) $$\;P_{global}\left(t\right)=\frac{1}{1+e^{-\left(s\left(t\right)-\tau \right)}},\;\;\tau =3$$

The logistic form is preferred to linear or exponential alternatives because it stays in (0,1) for every counter value, which removes any need for clipping. It also places the midpoint at s = τ, giving the threshold an operational interpretation. The value τ is the stagnation length at which the global and local search probabilities are equal. Around this threshold, the response saturates and flattens over roughly two counter units. At s = τ−2, P ≈ 0.12, while at s = τ + 2, P ≈ 0.88. Short interruptions in the improvement signal, therefore, hardly perturb the routing, while prolonged stagnation reliably ends in a global search. Transient fluctuations in the improvement signal are dampened rather than amplified. The rule enables a seamless restart when stagnation occurs. Progress continues while profitable. Repeated failure steadily raises the chance of an oppositional global search, and any success resets the counter. Because the best record is updated only through exact evaluations, no routing decision can jeopardize the existing solution. Saturation limits sensitivity to the threshold itself. Shifting by one unit moves the response curve sideways by one stagnation step and leaves its shape untouched.

### 5.3. Surrogate and Archive Management

A single repository A of precisely assessed points serves all surrogate mechanisms. It is managed on a first-in, first-out (FIFO) basis with capacity |A|=5N. Every exact evaluation enters the archive, whether it comes from initialization, the WHALE sweep, exact-mode opposites, or the exact re-evaluation of inheritance-mode survivors. And the oldest entries are discarded once the capacity is exceeded. The FIFO principle keeps the archive focused on the most recently visited region, which is where both approximators are queried.

Neither approximator is trained. Fitness inheritance evaluates a candidate directly against four randomly selected members of the population. The GRNN predicts from the k-nearest archive points. In both cases, the archive is the model. The iterative retraining cost that usually accompanies surrogate methods is, therefore, avoided by construction, and the remaining costs of neighbor search and kernel evaluation are detailed in Section 5.4.

Only one GRNN hyperparameter exists, the bandwidth σ, and it is adapted once per refinement invocation. The archive diversity and its running maximum are:

(46) $$Div_{arch}\left(t\right)=\frac{1}{D}\sum_{j=1}^{D}std\left(x_{1,j},\ldots ,x_{|A|,j}\right),\;\;x_{i}\in A$$

(47) $$Div_{max}\leftarrow max\left(Div_{max},\;Div_{arch}\left(t\right)\right)$$

The kernel mass of each archive point over its $k_{SCI}=10$ nearest archive neighbors, and the resulting separation coverage index, are:

(48) $$W_{i}=\sum_{j\in kNN_{i}}exp\left(-\frac{d_{ij}^{\;2}}{2\sigma^{2}}\right)$$

(49) $$SCI\left(t\right)=\frac{1}{\left|A\right|}\sum_{i=1}^{\left|A\right|}\frac{1}{max\left(W_{i},\epsilon \right)}$$

A large SCI indicates that archive points are mutually isolated at the current bandwidth; a small SCI signals dense mutual coverage. The adaptive target couples the acceptable isolation to the search state:

(50) $$\begin{array}{l} {\;\tau }_{t}=\tau_{min}+\left(\tau_{max}-\tau_{min}\right)\left(1-\frac{Div_{arch}\left(t\right)}{Div_{max}+\epsilon }\right),\\ \tau_{min}=0.05,\;\tau_{max}=0.25 \end{array}$$

so that a contracted archive ($Div_{arch}\ll Div_{max}$) raises the target. Higher apparent isolation is thus tolerated once the search has genuinely narrowed. The bandwidth then follows a bounded multiplicative rule:

(51) $$\begin{array}{l} \sigma \leftarrow clip\left(\sigma \;e^{\;\alpha \;\left(SCI\left(t\right)-\tau_{t}\right)},\;10^{-3},\;10\right),\\ \alpha =0.05,\;\sigma \left(0\right)=1 \end{array}$$

The rule widens the kernels when the archive is under-covered and narrows them when it is over-smoothed. The internal control parameters of SAWHALE are reported in Table 4.

#### Prediction-Error Containment

Within the framework, approximation errors are limited by one structural feature. Surrogate estimates are used only for ranking, so no estimated value is retained. Under inherited pricing, parents compete with their counterparts for survival under Equation (28), and each surviving new position is then evaluated. The GRNN ranks the eight local contenders, yet the winner is evaluated before storage in the next iteration, so no projected fitness can reach the population fitness vector (beyond the final, budget-exhausted population), the archive, or the best-so-far trajectory. The best-so-far value changes only through exact evaluations, so a consistently skewed prediction can mislead a single candidate selection for one cycle at worst. It cannot persist because a later accurate assessment supersedes it. Three additional safeguards reduce the risk further. The FIFO archive discards inferior regions, which limits stale guidance. The bandwidth is confined to $\left[10^{-3},10\right]$, which constrains the kernel range. The exact-scoring mode of Equation (22) intermittently refreshes the archive with newly evaluated points.

### 5.4. Computational and Memory Complexity

Every neighbor search relies on vectorized brute-force distance computation, so all the above costs are exact and independent of any data structure. No spatial index is assumed, and the above orders hold even in high dimensions, where tree-based data structures degenerate to linear scans. For M≤N/2 and K=3, the aggregate set discussed in Section 4.2.5 satisfies $n_{comb}=N+MK\leq 2.5N$, and the archive satisfies |A|≤5N.

The totals follow directly. The per-iteration time complexity is $O\left(N^{2}D\right)$, and the three exhaustive neighbor calculations dominate it. Because the number of iterations is bounded by $FES_{max}/N$, the overall time complexity is $O\left(FES_{max}\;N\;D\right)$, which is linear in the evaluation budget. The memory footprint of the algorithmic state, which includes the population, the archive, and the candidate buffers, is $O\left(ND\right)$. Only the per-evaluation convergence history scales with the budget $O\left(FES_{max}\;D\right)$. This experimental instrumentation can be switched off at deployment.

The two phases are unequal by design, and the analysis keeps them separate rather than blending them. The complete local phase consumes no exact evaluations, and the only consumer after the primary sweep is the global exact-mode invocation, which uses up to MK=1.5N. In floating-point operations, the balance shifts. The global phase spends its work on candidate creation, pricing, and selection, while the local phase spends it mainly on GRNN screening and bandwidth adjustment. The framework’s complexity is, therefore, split between two budgets. Global probing is evaluation-intensive, while local screening is computation-intensive, as Table 5 shows. Neither phase carries a surrogate training cost. Both approximators are lazy learners, whose model is the archive itself, and the neighbor-search and kernel costs that replace training are exactly the ones tabulated above.

## 6. Numerical Experiments on SAWHALE

This section reports the complete experimental campaign. Section 6.1, Section 6.2, Section 6.3, Section 6.4, Section 6.5, Section 6.6 and Section 6.7 evaluate the proposed operators and the full framework on the CEC 2014 and CEC 2017 suites, and Section 6.8 and Section 6.9 then carry the framework to constrained engineering design and to feature selection. Unless a subsection states otherwise, every configuration is run 30 times independently, and all reported statistics are computed from exact objective values.

### 6.1. Performance Analysis of the Proposed Oppositional-Based Learning Variants

The first experiment isolates the two proposed operators at the component level. Each of the eight oppositional variants grafts one opposition scheme onto an otherwise identical WHALE host, so that any performance difference is attributable to the opposition stage alone. WHALE-EDOFAS and WHALE-OPADAMP carry the operators of Section 4.2 and Section 4.3. The six comparison variants carry the canonical opposition rule of Tizhoosh (WHALE-OBL) [18] and five of its established refinements: quasi-opposition (WHALE-QOBL) [20], elite opposition (WHALE-EOBL) [23], centroid opposition (WHALE-COBL) [21], dynamic-opposite learning (WHALE-DOBL) [22], and generalized opposition-based learning (WHALE-GOBL) [24]. The unmodified WHALE completes the field of nine. All algorithms were run on twenty shifted benchmark functions covering unimodal, ill-conditioned, and multimodal landscapes at D = 500 and D = 1000, with a budget of 10^5^ exact function evaluations per run and 30 independent runs per configuration. Table 6 and Table 7 report the mean and standard deviation of the final objective error at 500 dimensions and Table 8 and Table 9 at 1000 dimensions, and Table 10 and Table 11 summarize the Friedman analysis with Iman–Davenport correction and Nemenyi post hoc comparison at α = 0.05.

Table 6 covers the unimodal and ill-conditioned half of the suite at D = 500, and its clearest feature is how quickly the field separates there. The Generalized Dixon–Price function (F2) separates it least. Five variants, including the unmodified baseline, reach the optimum exactly (OPADAMP, EDOFAS, OBL, COBL, and WHALE all report 0.0000E+00), so the function discriminates only against the aggressive reflection schemes, for which QOBL, DOBL, and GOBL retain errors between 2.3E+02 and 5.2E+02. For everywhere else, the ordering is one-sided. WHALE-EDOFAS records the lowest mean on eight of the remaining nine problems in the table (F1, F3, F5–F8, and F10, plus the F2 tie), often by margins that span orders of magnitude. On the Bent Cigar function (F7), whose conditioning of 10^6^ punishes any residual error along the sensitive axis, EDOFAS reaches 9.2656E+02, while OPADAMP reaches 1.5050E+04, the baseline 1.1838E+05, and the aggressive variants between 1.65E+08 and 3.85E+08, a spread of six orders of magnitude produced by nothing but the choice of opposition rule. The Quadratic function (F6) tells the same story at a smaller scale, with 1.0827E-01 for EDOFAS against 8.0390E+01 for the baseline and 1.7408E+05 for GOBL.

Two patterns in Table 6 deserve mechanistic comment. The first is the near-identical clustering of QOBL, DOBL, and GOBL on the shifted Rosenbrock function (F1), at 4.9590E+02, 4.9612E+02, and 4.9407E+02. Three unrelated algorithms agreeing to four significant figures is not search variability. It is the signature of collapse onto a common attractor, and the attractor is where single-center reflection rules place it. On shifted problems, the optimum sits away from the domain center, which is exactly the regime in which center bias is documented to be most damaging [19], and Section 4.1 argued on geometric grounds that every single-center scheme keeps pulling candidates back toward that center. The plateau makes the argument empirical. The second pattern is the column of NaN entries on the Sum of Different Powers function (F5). This objective raises coordinate magnitudes to exponents that grow with the index, up to the order of the dimension, so any solution with a coordinate outside the unit band overflows IEEE double precision and the reported mean becomes non-finite. Six of the nine variants, the baseline among them, fail this representability test at D = 500. Only three return finite errors, and their values rank the operators cleanly, at 3.3333E-07 for EDOFAS, 2.5667E-06 for OPADAMP, and 7.9256E-01 for GOBL. Finiteness on F5 is itself an accuracy statement, as it certifies that every coordinate of every returned solution lies inside the unit band. The two exceptions to EDOFAS’s dominance in this table are also informative. On the max-norm Schwefel 2.21 function (F4), where progress requires attacking the single worst coordinate, the diversification-oriented OPADAMP is the best variant at 1.9310E-03. On Zakharov (F9), no variant is credible. All nine stall at errors of order 10^5^, and the lowest mean belongs to DOBL at 7.0912E+04, whose long displacement steps are least penalized on a landscape none of the algorithms solves. This failure is reported as a shared limitation rather than a ranking result.

Table 7 moves to the multimodal half at D = 500, where opposition schemes are usually expected to earn their keep, and the proposed operators hold their positions. WHALE-EDOFAS has the lowest mean on eight of the ten problems (F11–F14 and F16–F19). On the shifted Rastrigin function (F12), it retains an error of 2.3461E-01 while the baseline retains 5.5721E+01 and the aggressive cluster again collapses onto a plateau, this time at 1.9912E+03 to 1.9943E+03 with standard deviations near one, as with the same common-attractor signature as on F1, now on a rugged landscape. On the Trigonometric function (F11), both proposed variants sit essentially at the basin floor (1.0004E+00 for EDOFAS, 1.0015E+00 for OPADAMP) while QOBL and DOBL remain near 1.2E+03. The Ackley (F17) and Griewank (F13) results, 8.2409E-03 and 4.8667E-06 for EDOFAS, show that the entropy-scheduled probes do not disrupt fine convergence on landscapes whose global structure is easy but whose local structure is noisy. On HGBat (F15), centroid opposition is the best variant at 2.7828E-01, and EDOFAS (5.5580E-01) trails even the baseline (4.5899E-01), the single problem in the suite where this happens. On the Deflected Corrugated Spring function (F20), OPADAMP takes its second individual win at 7.4998E-02, consistent with its role as the diversification specialist. Across the twenty functions at D = 500, every best result except F9 and F15 belongs to one of the two proposed variants, which is what the fractional best-function credits of Table 10 (15.2 for EDOFAS, 2.2 for OPADAMP) express in aggregate form.

**Figure 2:**
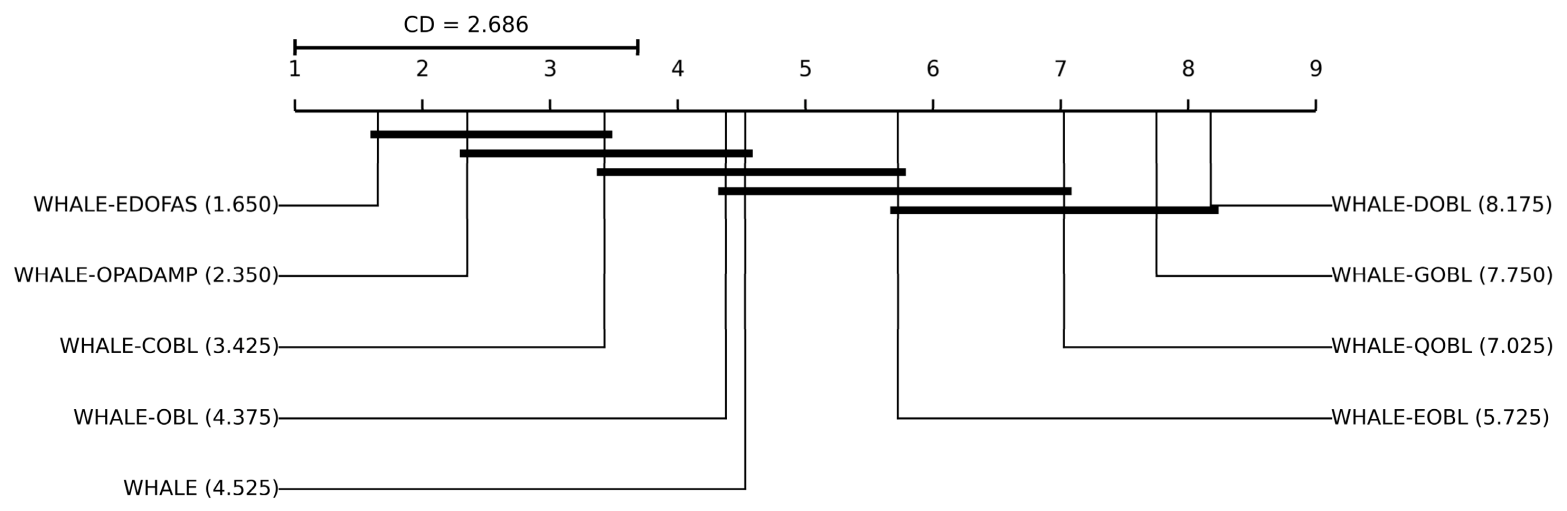
Nemenyi critical difference diagram for the nine WHALE variants over the 20 benchmark problems at D = 500 (α = 0.05).

The Friedman analysis confirms what the tables show function by function. The Friedman test, the Iman–Davenport correction, and the Nemenyi post hoc analysis are applied to the twenty benchmark functions to evaluate the nine WHALE variants. The tie-corrected Friedman test rejects equal performance ($\chi_{F}^{2}\;$= 124.410, *df* = 8, *p* = 4.07 × 10^−23^), and the Iman–Davenport statistic confirms the rejection (*F_F_* = 66.418, *df_1_* = 8, *df_2_* = 152, *p* < 0.001). Table 10 reports the Friedman ranking with fractional best-function allocations. WHALE-EDOFAS ranks first, WHALE-OPADAMP second, and WHALE-COBL third, with mean ranks of 1.65, 2.35, and 3.35. The Nemenyi critical-difference diagram in Figure 2 shows that WHALE-EDOFAS significantly outperforms WHALE-OBL, the standard WHALE, WHALE-EOBL, WHALE-QOBL, WHALE-GOBL, and WHALE-DOBL, while WHALE-OPADAMP significantly outperforms WHALE-EOBL, WHALE-QOBL, WHALE-GOBL, and WHALE-DOBL. The two proposed algorithms do not differ significantly from each other; their rank difference of 0.70 is below the critical distance.

Doubling the dimension changes the numbers but not the structure. In Table 8, the per-function winner map at D = 1000 is identical to the 500-dimensional one, with EDOFAS lowest on the same sixteen functions, OPADAMP on F4 and F20 plus the F2 tie, COBL on F15, DOBL on F9. So, the component ranking is stable under a twofold increase in dimensionality. The absolute degradation of the proposed operators is mild. EDOFAS moves from 1.6325E-02 to 4.1644E-02 on Rosenbrock and from 9.2656E+02 to 1.8504E+03 on Bent Cigar. The classical plateaus, by contrast, track the dimension exactly. The Rosenbrock cluster shifts from roughly 4.95E+02 to 9.91E+02–9.95E+02 with standard deviations below one (9.9377E+02 ± 0.85 for QOBL, 9.9454E+02 ± 0.57 for DOBL, 9.9078E+02 ± 0.56 for GOBL), and the Rastrigin plateau doubles from about 1.99E+03 to 3.98E+03–3.99E+03. Scale-invariant collapse of this kind confirms that the failure is structural and not budget-limited. More dimensions simply move the common attractor further from the shifted optimum. Dimension also extends the numerical-representability filter. At D = 1000, the coordinate product inside Schwefel 2.22 (F8) multiplies a thousand factors, and the same six variants that overflow on F5 now overflow on F8 as well. Meanwhile, EDOFAS (6.8612E-01) and OPADAMP (2.6886E+00) remain finite, and GOBL survives representability at a distant 4.0553E+02. A problem pair that was merely hard at 500 dimensions becomes, at 1000, a pass/fail test that only the proposed operators pass with competitive accuracy.

Table 9 completes the 1000-dimensional picture on the multimodal half and adds the scaling synthesis. EDOFAS again leads on eight of ten (Rastrigin at 6.2749E-01 against a classical plateau of 3.98E+03; Trigonometric at 1.0011E+00; Griewank at 8.2667E-06; Ackley at 9.1539E-03). COBL keeps HGBat, and OPADAMP keeps the spring function, where its error actually improves with dimension, from 7.4998E-02 to 6.3399E-02. Measured as the median ratio of 1000-dimensional to 500-dimensional means over the finite entries, the flattest scalers are COBL (×1.38) and the baseline (×1.41), followed by OPADAMP (×1.66), with EDOFAS at ×2.00. The ratio, however, is computed from the lowest base in the field, so the absolute gaps widen rather than close. On Bent Cigar, the distance between EDOFAS and the best classical variant grows from a factor of 95 to a factor of 137. On Schaffer F6, the EDOFAS error rises from 21.2 to 51.9, while DOBL rises from 920 to 1778. Two robustness counts summarize the component value of each operator. WHALE-OPADAMP beats the unmodified baseline on 17 of 20 functions at 500 dimensions and 16 of 20 at 1000, so the diversification operator is almost never harmful, even where it is not the winner. WHALE-EDOFAS is at least as good as the baseline on 19 of 20 functions at both dimensions, with HGBat the sole exception.

**Figure 3:**
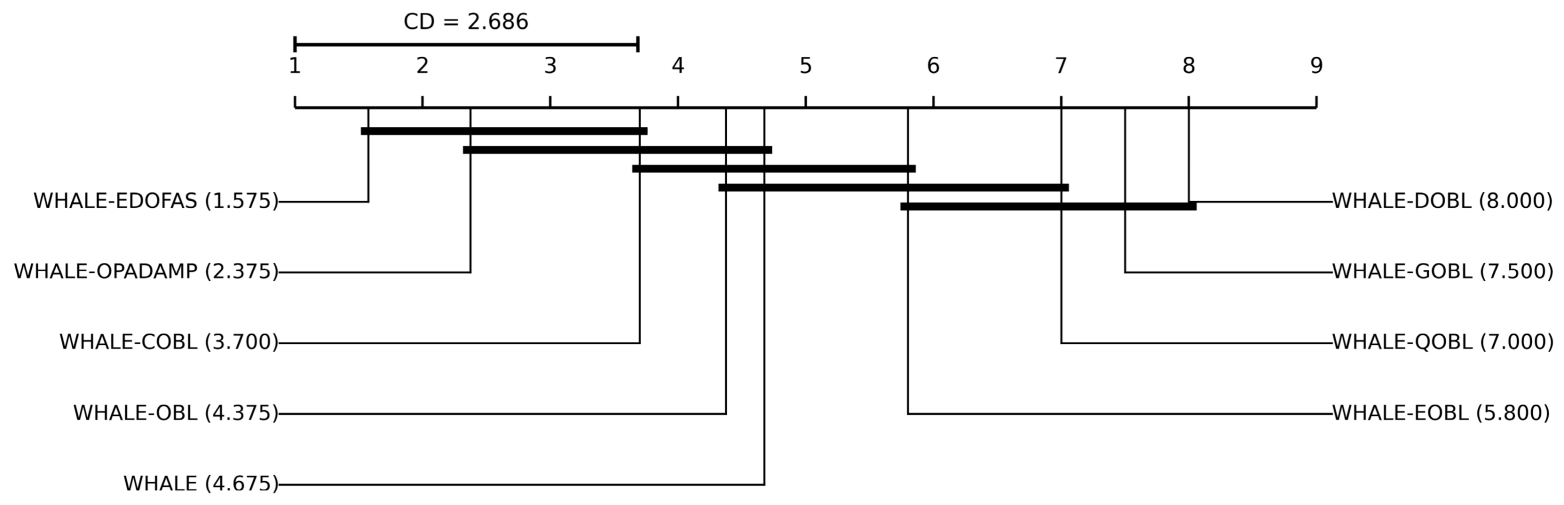
Nemenyi critical-difference diagram for the nine WHALE variants over the 20 benchmark problems at D = 1000 (CD = 2.686, α = 0.05).

The tie-corrected Friedman test rejects the null hypothesis of equal performance ($\chi_{F}^{2}\;$= 119.866, *df* = 8, *p* = 3.53 × 10^−22^), and the Iman–Davenport statistic confirms it (*F_F_* = 56.747, *df_1_* = 8, *df*_2_ = 152, *p* < 0.001). Table 11 shows WHALE-EDOFAS first overall, with an average rank of 1.575, followed by WHALE-OPADAMP at 2.375 and WHALE-COBL at 3.450. Figure 3 (CD = 2.686, α = 0.05) shows that WHALE-EDOFAS significantly outperforms the original WHALE, WHALE-EOBL, WHALE-QOBL, WHALE-DOBL, and WHALE-GOBL, while WHALE-OPADAMP significantly outperforms WHALE-EOBL, WHALE-QOBL, WHALE-DOBL, and WHALE-GOBL.

Taken together, the component study assigns the two operators distinct and complementary roles. EDOFAS is an accuracy engine, as it obtains sixteen individual wins out of twenty at both dimensions, finite and near-optimal behavior on the two overflow-prone objectives, and the top Friedman rank with the broadest statistical superiority. OPADAMP is the safe diversifier, which is rarely the single best, almost never worse than the host it modifies, and the strongest variant on the two landscapes (F4, F20), where progress depends on perturbing the worst coordinates rather than refining the best ones. The classical single-center schemes, and above all the three aggressive reflections, degrade through a mechanism that the tables expose directly and collapse onto a dimension-scaled attractor near the domain center. These roles motivate the architecture of SAWHALE, in which the two operators are not alternatives but stages, and the next experiments evaluate that combination against external state-of-the-art competitors.

### 6.2. Comparative Performance Analysis on the CEC 2014 Benchmark Suite

This section compares SAWHALE with five surrogate-assisted optimizers on the CEC 2014 suite. The five methods have been described in Section 2 already, and they will not be described in detail again. Briefly, TLSAPSO [27] carries a two-layer hierarchical surrogate inside a particle swarm, ESPSO [31] fits a radial basis model to the personal best history of its swarm, MSASFS [32] joins a cubic radial basis function with a Gaussian process, GPEQI [33] leans on a quadratic interpolation surrogate, and NRO [34] obtains a descent direction from local linear regression. The suite itself contains thirty functions. Functions F1 to F3 form the rotated unimodal group. F4 to F16 form the shifted and rotated multimodal group. F17 to F22 are the hybrid problems, and F23 to F30 are the composition problems. Tests are made at 30 and at 50 dimensions. A practical difficulty here is that the five comparators were published under budgets that differ a lot from each other, so some common ground had to be chosen. The budget of 50·D exact function evaluations was selected for all six algorithms, since the original NRO paper [34] used exactly this budget. And each configuration was repeated for 30 independent runs. Whatever the surrogates compute internally is not charged to this budget. Table 12 and Table 13 list the mean and the standard deviation of the final objective errors for the two dimensions.

Table 12 covers the 30-dimensional case. The multimodal group is where SAWHALE separates itself. It obtains the lowest mean error on eleven of the thirteen functions there, and the margins are not small ones. On the shifted Rastrigin function F9, its mean error is 8.0411E+01, roughly a third of the 2.4512E+02 that the closest competitor reaches, and on the shifted Schwefel function F11, the value of 4.1708E+03 stands against 7.5380E+03, so a little more than half. The unimodal side of the table looks completely different. GPEQI takes the best mean on all three rotated functions. This was expected because a quadratic interpolation surrogate is close to an exact model for landscapes of this type, and against such a modeling advantage, no diversification mechanism has much to offer.

The hybrid group reverses the situation. Not one of its six functions is won by SAWHALE at this dimension. NRO and TLSAPSO divide the best results between themselves, which matches the smooth function advantage that the NRO paper itself reports, and it also matches how Table 2 describes these two methods. As for the composition group, no single algorithm owns it. Four different methods share the best means, with SAWHALE keeping only F28 with 5.3289E+02. Over the whole table, SAWHALE ends up with twelve of the thirty best means, the largest share. NRO comes second with eleven, and neither ESPSO nor MSASFS manages a single best mean at 30 dimensions.

Table 13 tells a better story for SAWHALE. At 50 dimensions, its count of best means rises to fourteen, covering the entire multimodal family except F15 and adding the compositions F23 and F24, with the F23 mean being 3.7375E+02. NRO, meanwhile, falls from eleven wins to five, less than half of what it had before. GPEQI holds the unimodal group again, and on F1, it holds it by a distance that is hard to overstate, since its mean of 2.2767E+01 lies more than five orders of magnitude below every population-based competitor. Two weaknesses of SAWHALE do not go away at this dimension, and they should be written down as weaknesses. One is F15, the expanded Griewank plus Rosenbrock function, on which NRO stays better by an order of magnitude at both dimensions. The other is the hybrid group, where even at 50 dimensions, TLSAPSO wins five of the six pairwise comparisons. Beyond these two points, the message of both tables is the same message. The entropy-scheduled opposition and the archive-driven refinement, the two mechanisms that the component study examined, bring their benefit to the composite CEC landscapes on the multimodal core, and the benefit grows with the dimension instead of shrinking.

### 6.3. Statistical Ranking Analysis via the Friedman Test

**Figure 4:**
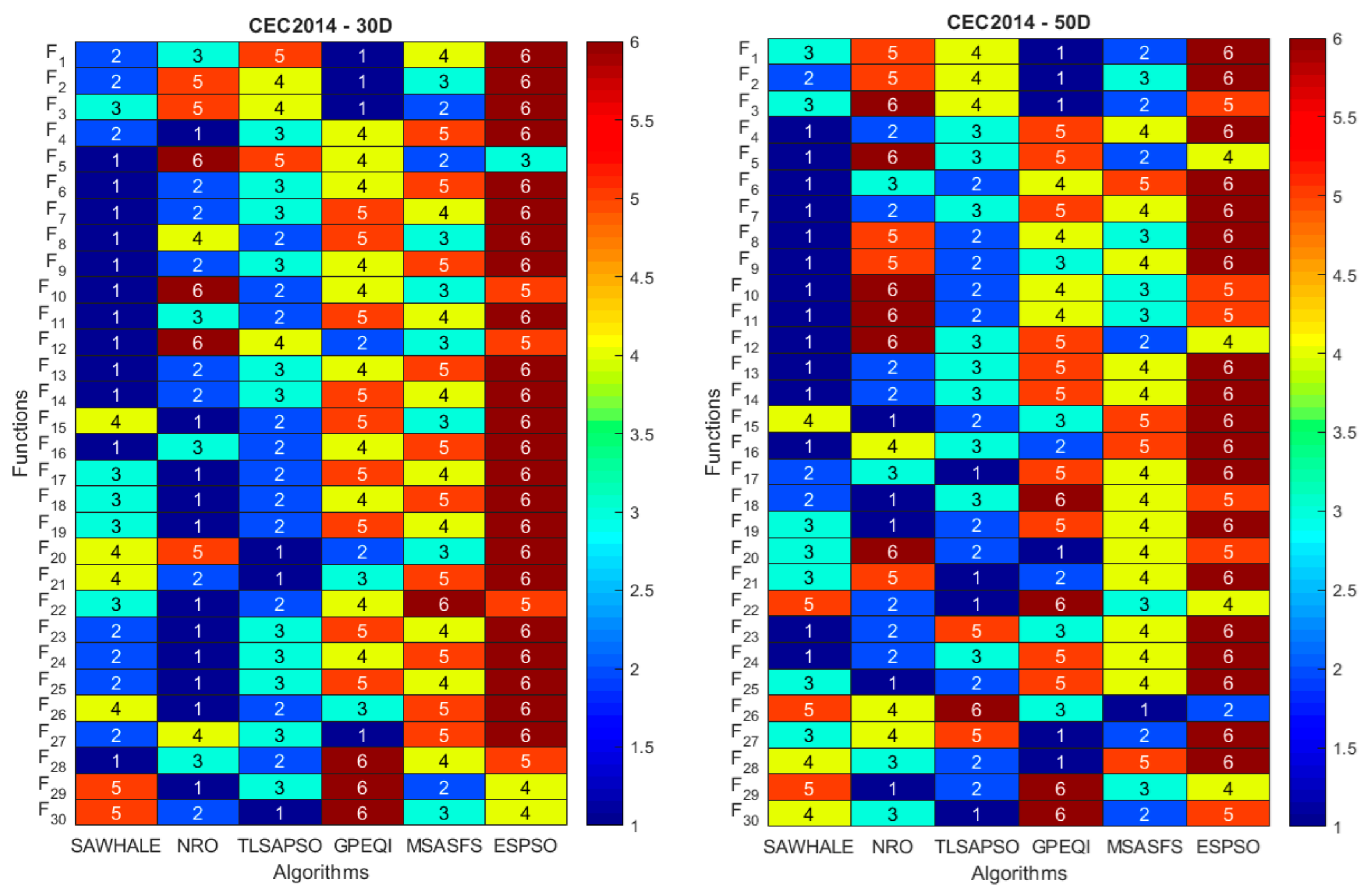
Friedman ranking heatmap of the six algorithms over the thirty CEC 2014 problems at 30 dimensions (left) and 50 dimensions (right); cell colors encode the per-function rank from blue (rank 1, best) to red (rank 6, worst).

All thirty comparisons are then compressed into one ranking per dimension with the Friedman test, and Figure 4 shows the resulting rank matrix as a heatmap. In the 30-dimensional case, the tie-corrected statistic comes out as $\chi_{F}^{2}\;$= 68.895 with *df* = 5, which corresponds to *p* = 1.74 × 10^−13^. The Iman–Davenport correction gives *F_F_* = 24.634 with *df_1_* = 5 and *df_2_* = 145, and equal performance is rejected with *p* < 10^−17^. The mean ranks are 2.233 for SAWHALE, 2.600 for NRO, 2.667 for TLSAPSO, 3.900 for GPEQI, 3.967 for MSASFS, and 5.633 for ESPSO. In the 50-dimensional case the rejection repeats, with $\chi_{F}^{2}\;$= 49.981, *p* = 1.40 × 10^−9^, *F_F_* = 14.492, and *p* < 10^−10^, and SAWHALE stays first with an average rank of 2.267, followed by TLSAPSO with 2.700, MSASFS with 3.433, NRO with 3.467, GPEQI with 3.733, and ESPSO with 5.400. NRO going down from second place to fourth place is worth a remark, because it repeats in rank form the performance loss that the tables showed between the two dimensions. What the heatmap adds is the location of all this. The SAWHALE row holds rank one on twelve functions at 30 dimensions and on fourteen at 50, and it never once enters the two worst rank levels, on any function, at either dimension. No other row can claim the same. The ESPSO row, for comparison, sits in those two worst levels on twenty-three of the thirty functions at 30 dimensions, more than three-quarters of the suite. The Nemenyi critical distance for six algorithms over thirty functions is CD = 1.377 at α = 0.05. Against this value, the mean rank of SAWHALE is significantly better than the ranks of GPEQI, MSASFS, and ESPSO at 30 dimensions and better than those of GPEQI and ESPSO at 50. The remaining distances, 0.367 and 0.434 to NRO and TLSAPSO at 30 dimensions and 0.433, 1.167, and 1.200 to TLSAPSO, MSASFS, and NRO at 50, all stay under the critical distance. So, the rank test settles half of the field and leaves a tie at the top, and that tie is taken apart function by function in the next subsection.

### 6.4. Wilcoxon Signed-Rank Analysis for the CEC 2014 Test Problems

The two-sided Wilcoxon signed-rank test is run at α = 0.05 between SAWHALE and every competitor on every function, over the thirty runs, and Table 14 collects the outcomes family by family. Summed over the suite, SAWHALE wins 14, 17, 20, 21, and 27 of the thirty comparisons against NRO, TLSAPSO, GPEQI, MSASFS, and ESPSO at 30 dimensions, and 21, 20, 19, 23, and 26 in the 50-dimensional case. Against ESPSO, this means all but three comparisons at 30 dimensions and all but four at 50. In all ten opponent and dimension pairs, the wins outnumber the losses, and the two methods that the Friedman averages could not separate from SAWHALE are included in this statement. SAWHALE loses at most one of its thirteen comparisons there against any opponent at either dimension, and against ESPSO and against MSASFS at 50 dimensions, it takes all thirteen. The hybrid block at 30 dimensions answers the second one. NRO wins five of those six comparisons, and TLSAPSO wins all six, and even at 50 dimensions TLSAPSO keeps five. So, the improvement with dimension is only a partial one. The composition block moves the other way, from a losing 0/3/5 record against NRO at 30 dimensions to a winning 3/3/2 at 50. None of this contradicts the Friedman ranking. The first place of SAWHALE rests on per-function superiority that concentrates on the multimodal core and strengthens with dimension, and the hybrid group stays the single region where two competitors keep a confirmed advantage.

### 6.5. Convergence Behavior on CEC 2014 Problems

**Figure 5:**
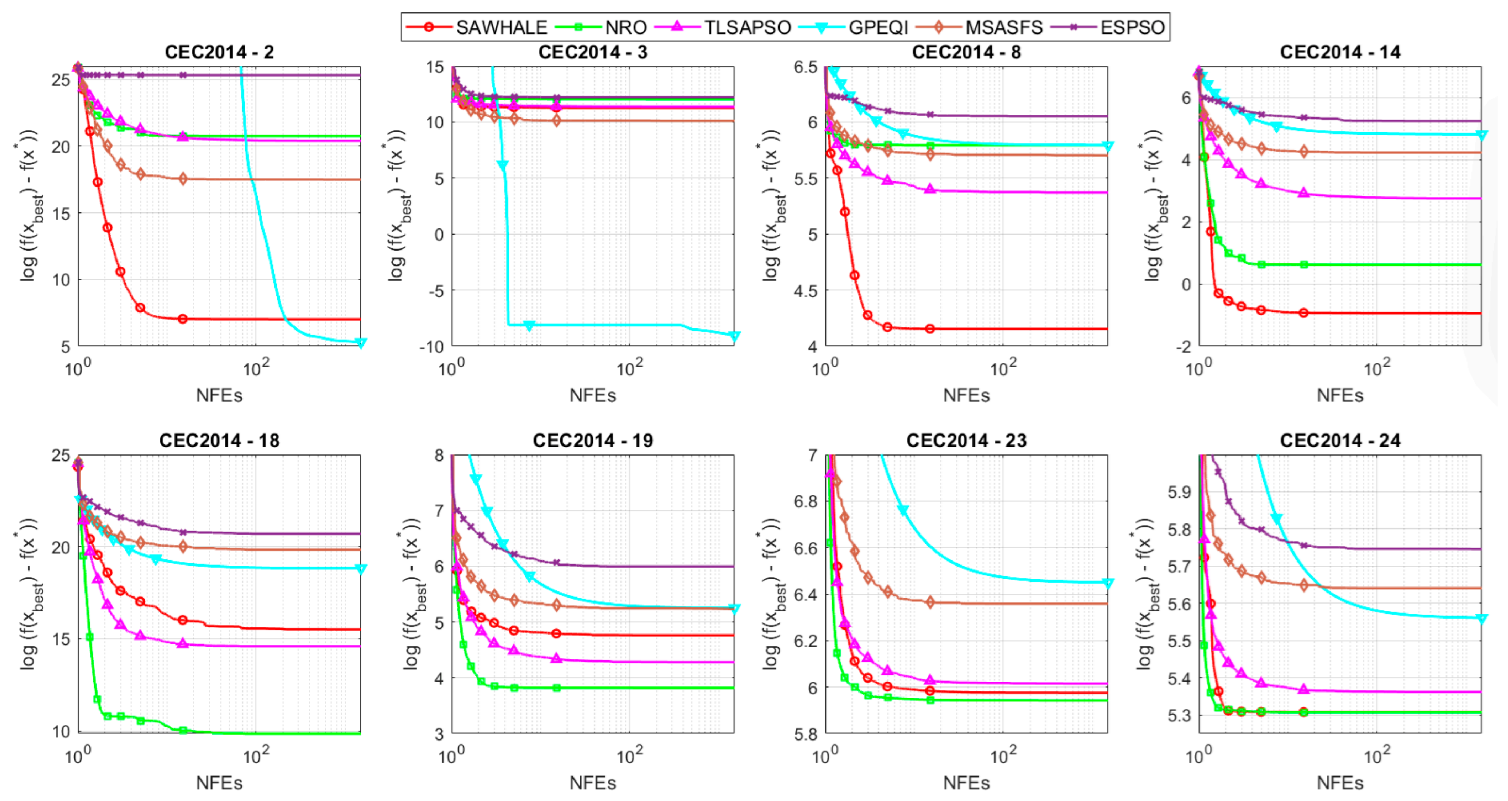
Convergence curves of the mean best-so-far objective error on eight representative CEC 2014 problems (F2, F3, F8, F14, F18, F19, F23, F24) at 30 dimensions, over the 50·D exact-evaluation budget (logarithmic axes).

**Figure 6:**
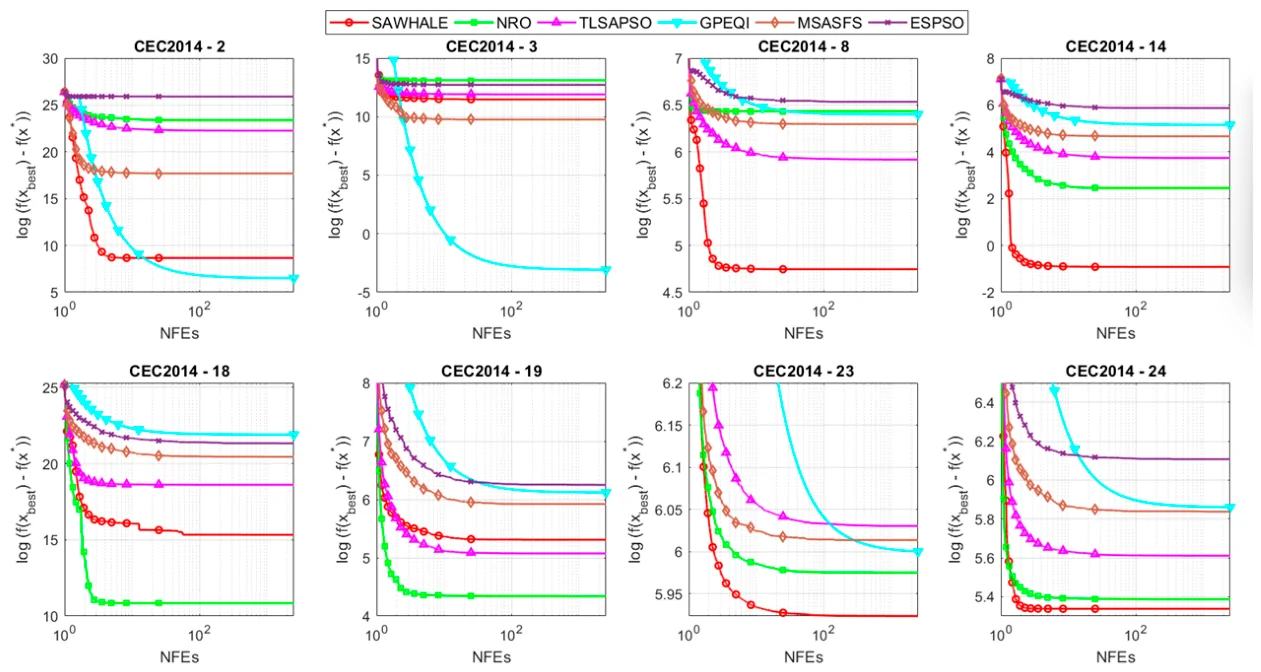
Convergence curves of the mean best-so-far objective error on the same eight CEC 2014 problems at 50 dimensions, over the 50·D exact-evaluation budget (logarithmic axes).

Figure 5 and Figure 6 plot the mean best-so-far error at 30 and at 50 dimensions, in that order, on eight functions picked so that each family appears twice: F2 and F3 for the unimodal group, F8 and F14 for the multimodal group, F18 and F19 for the hybrids, and F23 and F24 for the compositions. The horizontal axis counts the exact function evaluations up to the 50·D limit. One thing follows from the design before any curve is even read. Since surrogate-priced candidates cost nothing on this counter, a SAWHALE curve is made of exact evaluations only. All six methods are measured in one and the same currency, and every curve must end at the mean value that Table 12 or Table 13 report. What the curves add on top of that is timing. On the multimodal panels, SAWHALE drops fastest during the early part of the run, the part in which the logistic switch holds the exact-scoring global mode active and each EDOFAS call turns evaluation cost into archive content. Within roughly the first tenth of the logarithmic axis, its curve is already under the final levels of all competitors, so the margin seen at the end was collected at the beginning. On the unimodal panels, the GPEQI curve runs below every population-based competitor and is never caught. On the hybrid panels at 30 dimensions, the roles change hands, with the NRO and TLSAPSO curves finishing below the SAWHALE curve, and their final levels stay out of reach for the whole budget.

### 6.6. Ablation Analysis of the SAWHALE Components

The last experiment takes the framework apart piece by piece. Six ablated versions of SAWHALE are constructed for this purpose, and each of them removes or replaces exactly one design decision while everything else is kept as it is. The idea is simple. OBLREM removes the opposition-based learning module completely, so the surrogate-assisted and hybrid search parts must work without any oppositional input. SGLOBAL removes the local search phase, which leaves only the global component of the original architecture, and SLOCAL does the opposite, removing the global phase so that the search relies on the local exploitation mechanism alone. STDOBL keeps the whole architecture but replaces the proposed opposition strategy with a conventional opposition-based learning rule. SREMOVED strips the surrogate-assisted candidate generation out of both search stages, and this variant, therefore, gives a direct look at what the surrogate guidance is worth. RNDSWTCH keeps both phases and both surrogates but switches between the global and local search at random rather than through the adaptive logistic rule. The WHALE host itself is present in every variant. All seven algorithms are run on the CEC 2014 suite at 30 and at 50 dimensions, with the same 50·D budget of exact evaluations and 30 independent runs per configuration that the previous subsections used. The means and standard deviations are collected in Table 15 and Table 16.

Table 15 gives the outcome for 30 dimensions, and the full framework wins it, and wins it clearly. SAWHALE takes the lowest mean on twenty-seven of the thirty functions. On the thirteen multimodal problems, it beats each of the six variants on every single function, with no exception anywhere. Also, the margins in this group are far from small. On F9, the full version reaches 8.0411E+01 while the six variants stay between 3.2389E+02 and 3.8671E+02. And on F14, its value of 3.8749E-01 stands against the variant means that run from 1.3572E+02 up to 2.2536E+02. Nothing in this group is even close. OBLREM turns out to be the only variant that loses to the full framework on all thirty functions, and STDOBL loses on twenty-nine of them. The three functions that SAWHALE does not win are exactly the three unimodal ones, and all three of them go to SREMOVED. On F1, for instance, the surrogate-free variant reaches 1.8676E+03 while the full framework stays up at 3.0200E+07. Here the trade-off of the design shows itself in its plainest form. The surrogate and opposition machinery buys a large multimodal advantage at a real cost on smooth landscapes, and the ablation puts that cost on the table instead of hiding it.

As Table 16 shows, the same structure repeats at 50 dimensions, only with sharper edges. SAWHALE keeps twenty-four best means. The multimodal sweep against all six variants remains complete, with thirteen out of thirteen. And on F4, the full version holds 2.5341E+02, while the variants range from 1.6115E+04 up to 4.3395E+04. The unimodal side, on the other hand, becomes almost dramatic. SREMOVED brings F1 down to 1.2498E-09. SLOCAL reaches 2.5704E-03. RNDSWTCH reaches 2.1099E-02, and the full framework stays at 2.4112E+07. So, the six best means go to SREMOVED, which takes the three unimodal functions, the new best on F26, and the shared composition plateaus at F27 and F28. SLOCAL shares those two plateaus as well, and RNDSWTCH shares the one at F27.

Two composition results deserve a remark of their own. On F23, the full framework wins with 3.7375E+02, while five variants stop together on a plateau at or very near 4.0000E+02, which is the floor value of this composition. On F27, however, things turn around, and the full framework is the worst of the seven, with 4.6666E+02, while the same plateau holds the variants near 400. The full search keeps moving where the reduced versions stall, and on this one function the movement ends in a worse basin. These rows are kept on the paper on purpose because an ablation that shows only its flattering side would not be worth much.

For the ranking view, the Friedman test is applied once more, in the same manner as before. At 30 dimensions, the tie-corrected statistic is $\chi_{F}^{2}\;$= 110.071 with *df* = 6, corresponding to *p* = 1.97 × 10^−21^, and the Iman–Davenport value is *F_F_* = 45.648 with *df_1_* = 6 and *df_2_* = 174, so equal performance is rejected with *p* < 10^−32^. The mean ranks come out as 1.333 for SAWHALE, 2.733 for SLOCAL, 3.300 for RNDSWTCH, 4.200 for STDOBL, 4.567 for OBLREM, 5.733 for SGLOBAL, and 6.133 for SREMOVED. At 50 dimensions, the rejection repeats, this time with $\chi_{F}^{2}$ = 80.729, *p* = 2.53 × 10^−15^, *F_F_* = 23.583, and *p* < 10^−19^, and the ordering reads 2.033 for SAWHALE, 2.283 for SLOCAL, 3.400 for RNDSWTCH, 4.500 for STDOBL, 4.867 for OBLREM, 5.283 for SREMOVED, and 5.633 for SGLOBAL

**Figure 7:**
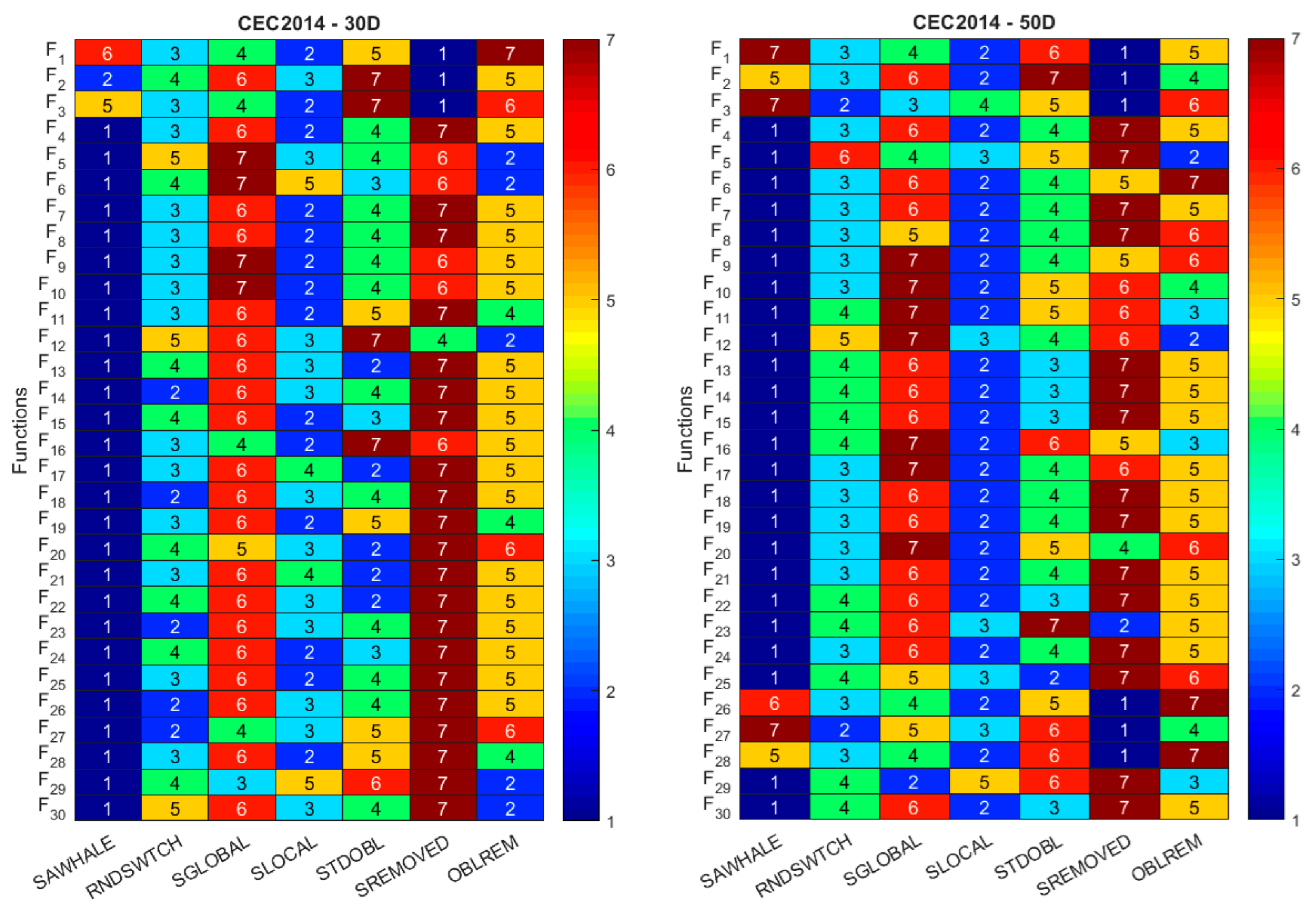
Friedman ranking heatmaps of the full SAWHALE and its six ablated variants over the thirty CEC 2014 problems at 30 dimensions (left) and 50 dimensions (right); cell colors encode the per-function rank from blue (rank 1, best) to red (rank 7, worst), with exact ties broken to integers for display.

The two rank matrices behind these averages are drawn as heatmaps in Figure 7, and the SAWHALE column there is almost uniformly at rank one. No other column comes close to this. Exact ties in the tables are broken to integers for this display. For seven algorithms over thirty functions, the Nemenyi critical distance is CD = 1.645 at α = 0.05. Held against this value, the full framework is significantly better than every variant except SLOCAL at 30 dimensions, where the distance of 1.400 stays just under the threshold. At 50 dimensions, it is significantly better than STDOBL, OBLREM, SREMOVED, and SGLOBAL, while SLOCAL at 0.250 and RNDSWTCH at 1.367 remain inside the tie region. So, the rank view alone leaves the closest variants unresolved, and the pairwise tests must take over from here.

Table 17 lists the two-sided Wilcoxon results at α = 0.05, family by family, seen from the full framework’s side. The totals come first, and they are one-sided. At 30 dimensions, SAWHALE wins 26, 26, 26, 27, 27, and 28 of the thirty comparisons against RNDSWTCH, SGLOBAL, SLOCAL, STDOBL, SREMOVED, and OBLREM, and the corresponding totals at 50 dimensions are 24, 25, 24, 26, 24, and 26. Even the two variants that the Nemenyi distance could not separate lose the large majority of the per-function contests. The multimodal rows are a wall, with thirteen wins and no losses in eleven of the twelve variant and dimension pairs and 12/1/0 in the remaining one. The hybrid rows contain no losses at all, and at 50 dimensions, every one of them reads 6/0/0. Where does the full framework lose? The unimodal rows give the answer. Since they carry every loss, it takes on smooth ground. There, SREMOVED wins all three functions at both dimensions, and at 50 dimensions, four variants do the same. The composition rows stay favorable but not perfect, and the losses that appear there at 50 dimensions include the F26 and F27 cases discussed above.

**Figure 8:**
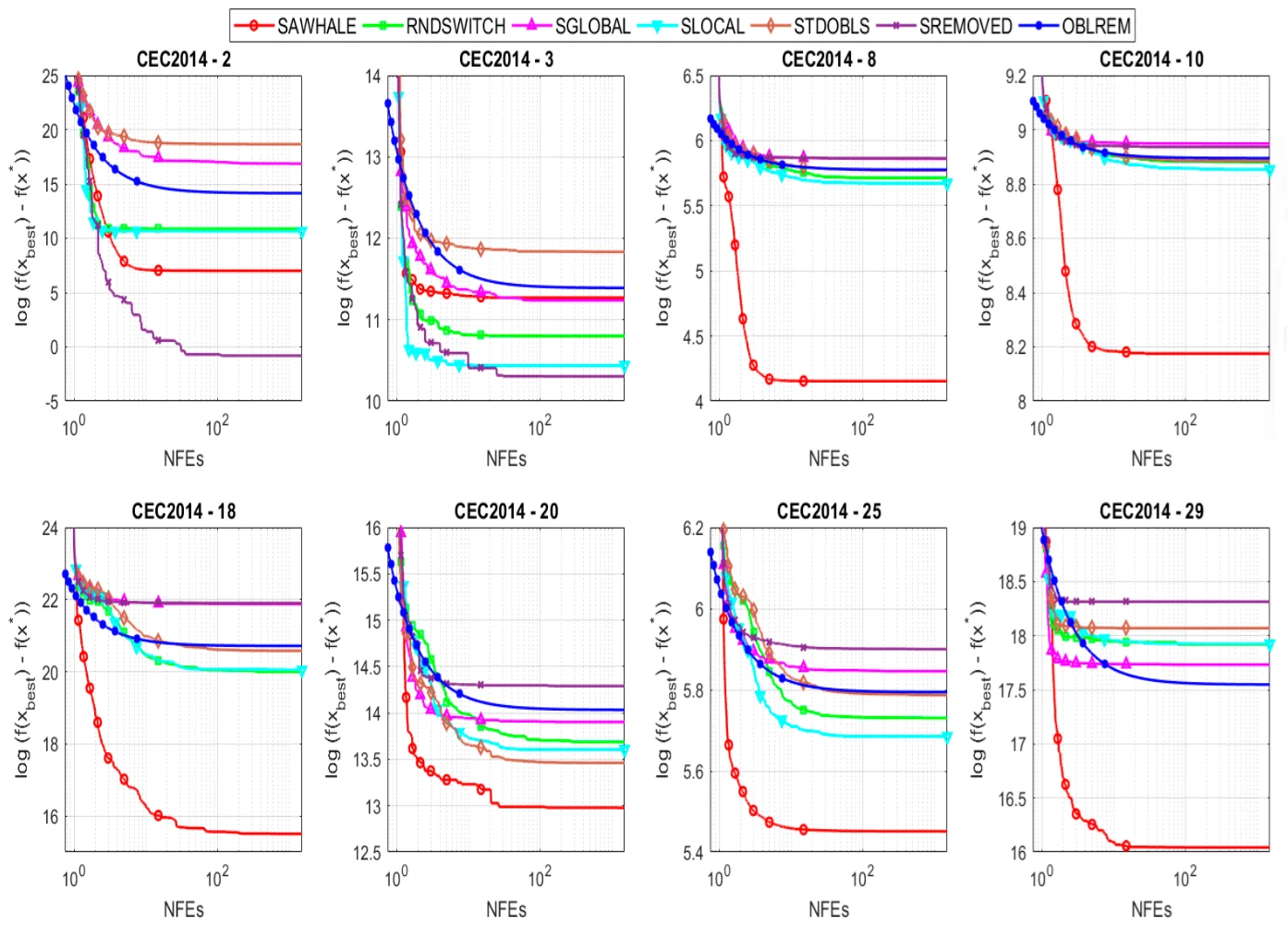
Convergence curves of the mean best-so-far objective error for the full SAWHALE and its six ablated variants on eight representative CEC 2014 problems (F2, F3, F8, F10, F18, F20, F25, F29) at 30 dimensions (logarithmic axes; budget 50·D exact evaluations).

**Figure 9:**
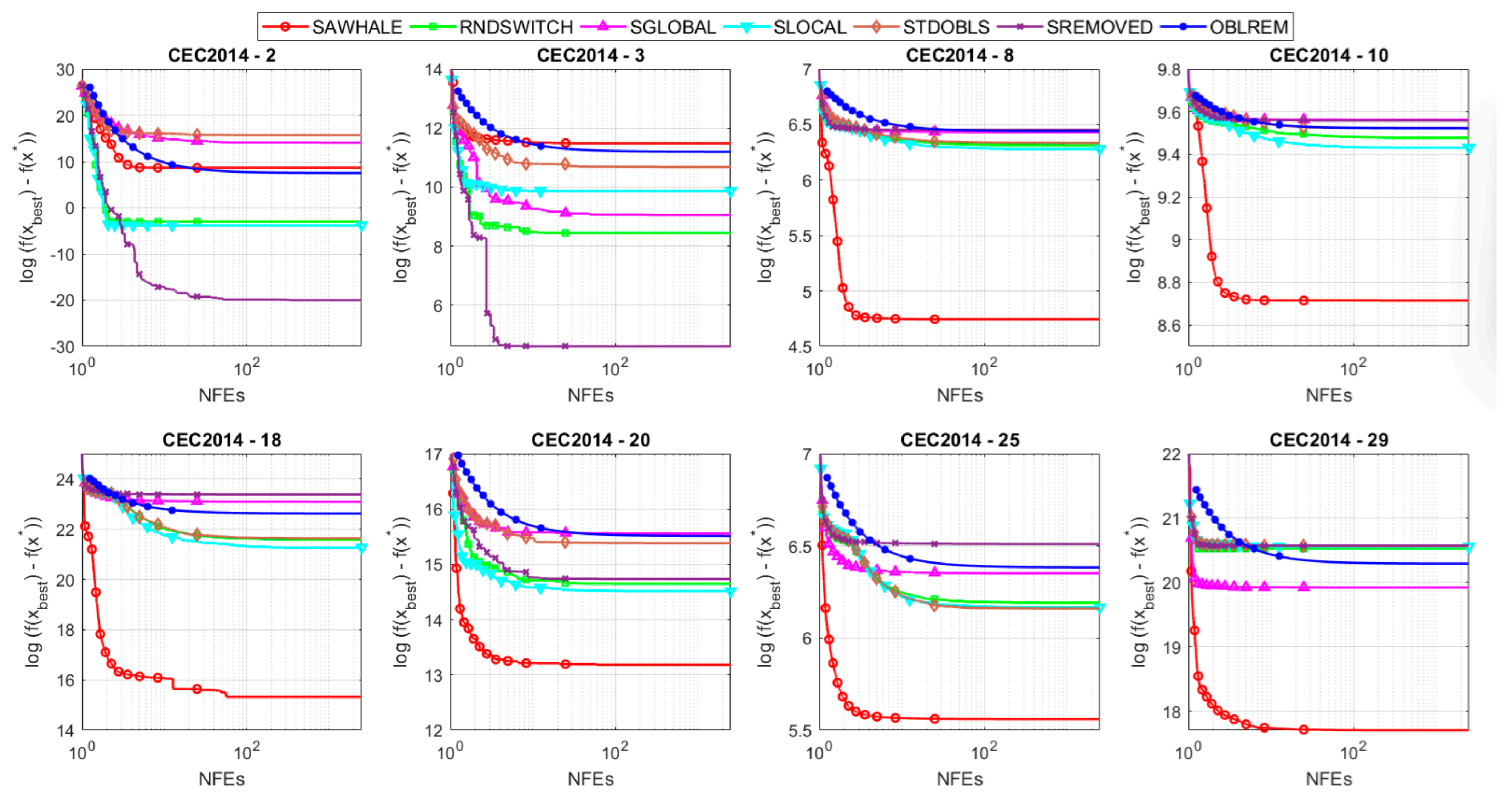
Convergence curves of the same seven algorithms on the same eight CEC 2014 problems at 50 dimensions.

Figure 8 and Figure 9 close the section with the convergence curves at 30 and at 50 dimensions, plotted for eight functions that cover the four families. These are F2, F3, F8, and F10 in the upper rows and F18, F20, F25, and F29 in the lower ones. The reading rules stay the same as in Section 6.5. Every curve spends only exact evaluations, and every curve must end at the mean that Table 15 or Table 16 report. On the multimodal panels, the full SAWHALE curve separates from the six variant curves early in the run and never gives the separation back, which is simply the temporal face of the thirteen-out-of-thirteen rows in Table 17. On the unimodal panels, the picture inverts, and several variant curves, the SREMOVED curve above all, dive far below the full one, exactly as the mean tables had already mentioned. The composition panels show the plateau behavior described earlier, with the variant curves flattening near the floor value while the full curve continues to move.

Read as a whole, the ablation attaches a measured price to every module. Removing the surrogate guidance is the most expensive single cut at 30 dimensions, where SREMOVED falls to the last average rank of 6.133, and removing the local phase costs nearly as much, SGLOBAL sitting at 5.733 and at 5.633 across the two dimensions. Replacing the proposed opposition scheme with the conventional rule costs 2.9 rank points at 30 dimensions and 2.5 at 50, and removing opposition altogether costs slightly more, which says something worth mentioning. Conventional opposition buys almost nothing over no opposition at all in this framework, so the benefit comes from the proposed operators themselves and not from the mere presence of an oppositional step. Randomizing the switch costs about two rank points at 30 dimensions and about 1.4 at 50, and these numbers are the measured value of the adaptive logistic rule. The mildest cut is the removal of the global phase. SLOCAL stays within the Nemenyi tie region, yet even this closest variant loses all thirteen multimodal comparisons at both dimensions. So the two phases are complements rather than substitutes. One honest caveat runs through everything above, and it should stay visible. Every module earns its keep on the multimodal core that the framework was built for, and every module has a price on smooth unimodal ground, where the leanest variants win.

### 6.7. Comparative Analysis of the CEC 2017 Benchmark Suite

The comparison so far has stayed inside the surrogate-assisted family, and a fair question is how the framework behaves against recent metaheuristics that spend their budget without any surrogate at all. This section answers it on the CEC 2017 suite. Six competitors are used, namely the Arithmetic Optimization Algorithm AOA [35], the Parrot Optimizer PARROT [36], the Sinh-Cosh optimizer SINHCOSH [37], and three recent whale optimization variants, DGSWOA [10], EWOA [11], and MHWOA [12]. The last three are of special interest here because they improve the same WHALE host that SAWHALE is built on, so the comparison also shows what the proposed additions are worth against other improvements of the same base. The CEC 2017 suite contains thirty functions, where F1 to F3 are unimodal, F4 to F10 are simple multimodal, F11 to F20 are hybrid, and F21 to F30 are composition problems. All seven algorithms run at 30 and at 50 dimensions under the common budget of 50·D exact function evaluations with 30 independent runs per configuration, exactly as in the previous subsections. Table 18 and Table 19 collect the means and standard deviations. One remark about F2 should be made before the tables are read. This function is numerically explosive. Its errors reach values around 10^44^ at 30 dimensions and around 10^83^ at 50, and single runs can dominate the reported means.

Table 18 gives the 30-dimensional set of predictions, and the proposed framework takes twenty-two of the thirty best means. The multimodal group is again a clean sweep. On all seven functions from F4 to F10, SAWHALE beats every one of the six competitors, without a single exception. The unimodal group is mixed, which the F2 remark above already suggested, and on F1 the difference is drastic. SAWHALE reaches 2.9569E+03 there while the six competitors sit between 2.7559E+10 and 5.8891E+10. That is, about seven orders of magnitude higher. The remaining eight best means belong to two algorithms. EWOA collects six of them, five inside the hybrid group together with the unimodal function F3, and PARROT takes the other two, one of which is the unstable F2. This makes EWOA the strongest rival in the field, and its strength is concentrated exactly where the CEC 2014 study already located the softest ground of the framework, on the hybrid functions. The composition group, on the other hand, belongs to SAWHALE almost completely, with nine of the ten best means in this dimension.

The 50-dimensional results in Table 19 move further in favor of the proposed framework. Its count of best means rises to twenty-five. The multimodal sweep from F4 to F10 stays complete against all six competitors, and the composition group is nearly complete as well, with nine of its ten functions. On F1, the gap keeps its size, 4.6493E+03, against competitor means between 6.9733E+10 and 1.1288E+11. The remaining five best means are split among EWOA with three, PARROT with one, and SINHCOSH with one. In short, the hybrid weakness that was visible at 30 dimensions shrinks when the dimension grows, which repeats the pattern of the CEC 2014 study, and the framework ends up closer to a full sweep at the higher dimension rather than further from it.

**Figure 10:**
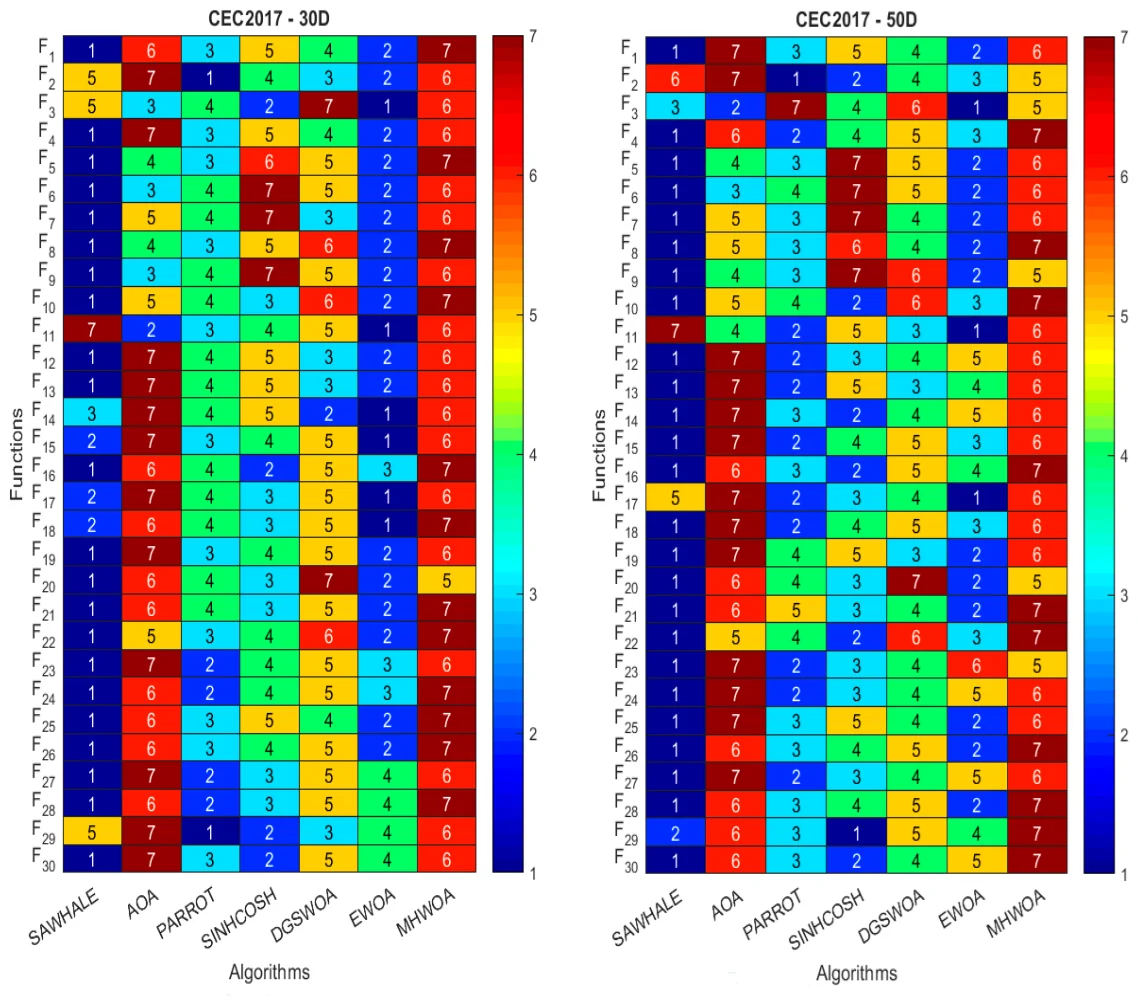
Friedman ranking heatmaps of SAWHALE and the six metaheuristic competitors over the thirty CEC 2017 problems at 30 dimensions (left) and 50 dimensions (right); cell colors encode the per-function rank from blue (rank 1, best) to red (rank 7, worst).

The Friedman test compresses these thirty comparisons in the usual way, and Figure 10 shows the two per-function rank matrices as heatmaps. At 30 dimensions, the statistic is $\chi_{F}^{2}$= 116.671 with *df* = 6, which corresponds to *p* = 8.14 × 10^−23^, and the Iman–Davenport correction gives *F_F_* = 53.427 with *df_1_* = 6 and *df_2_* = 174. So equal performance is rejected, with *p* < 10^−36^. The mean ranks come out as 1.767 for SAWHALE, 2.167 for EWOA, 3.167 for PARROT, 4.100 for SINHCOSH, 4.700 for DGSWOA, 5.733 for AOA, and 6.367 for MHWOA. At 50 dimensions, the rejection repeats with $\chi_{F}^{2}$ = 105.914, *p* = 1.46 × 10^−20^, and *F_F_* = 41.459, and the ordering reads 1.600 for SAWHALE, 2.933 for EWOA, 2.967 for PARROT, 3.900 for SINHCOSH, 4.567 for DGSWOA, 5.867 for AOA, and 6.167 for MHWOA. The Nemenyi critical distance for seven algorithms over thirty functions is CD = 1.645 at α = 0.05. Measured against it, SAWHALE is significantly better than SINHCOSH, DGSWOA, AOA, and MHWOA at both dimensions. EWOA and PARROT stay inside the tie region, with distances of 0.400 and 1.400 at 30 dimensions and 1.333 and 1.367 at 50. So, the rank view separates the bottom four but not the top three, and the pairwise tests are needed once more to finish the job.

Table 20 lists the two-sided Wilcoxon outcomes at α = 0.05, family by family, from the side of the proposed framework. Summed over the suite, SAWHALE wins 26, 24, 24, 25, 21, and 27 of the thirty comparisons against AOA, PARROT, SINHCOSH, DGSWOA, EWOA, and MHWOA at 30 dimensions, and the totals at 50 dimensions are 28, 27, 25, 27, 26, and 29. Every total is a clear majority, and this includes the two algorithms that the Nemenyi distance could not separate. The multimodal rows are perfect, with seven wins and nothing else in all twelve variant and dimension pairs. The composition rows lose at most one comparison anywhere. The hybrid rows carry almost every loss the framework takes, and the EWOA column is the visible pressure point there, with a 4/4/2 record at 30 dimensions that improves to 8/0/2 at 50. The unimodal rows need one honest comment. Against MHWOA at 50 dimensions, the table reports three wins, although the SAWHALE mean on F2 is higher than the MHWOA mean. The F2 standard deviation of SAWHALE is about five times its own mean at this dimension. Single runs dominate that average, and the signed-rank test works on the paired runs rather than on the means. So a significant win with a worse mean is a property of this unstable function and not an inconsistency of the table.

**Figure 11:**
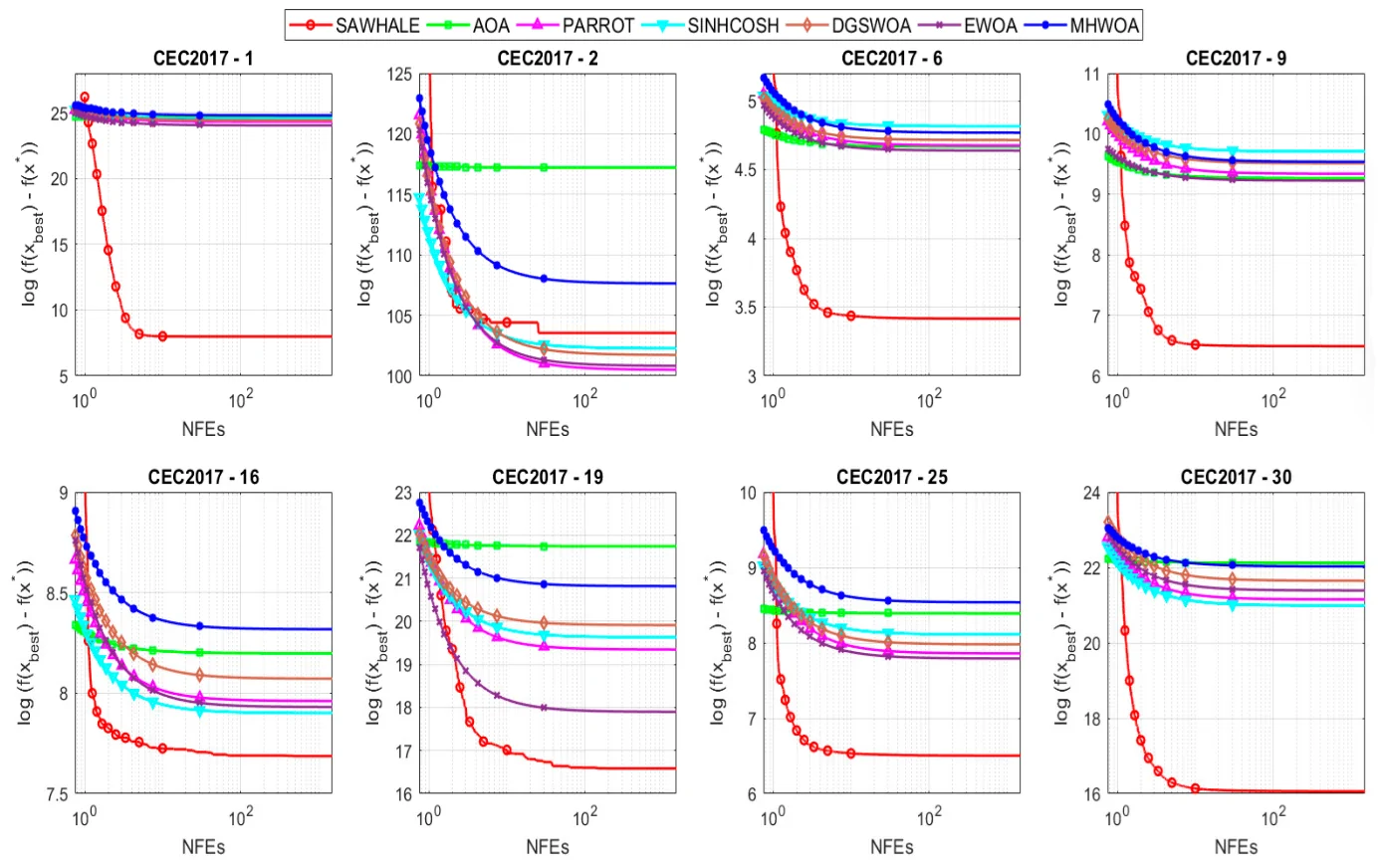
Convergence curves of the mean best-so-far objective error for the seven algorithms on eight representative CEC 2017 problems (F1, F2, F6, F9, F16, F19, F25, F30) at 30 dimensions (logarithmic axes; budget 50·D exact evaluations).

**Figure 12:**
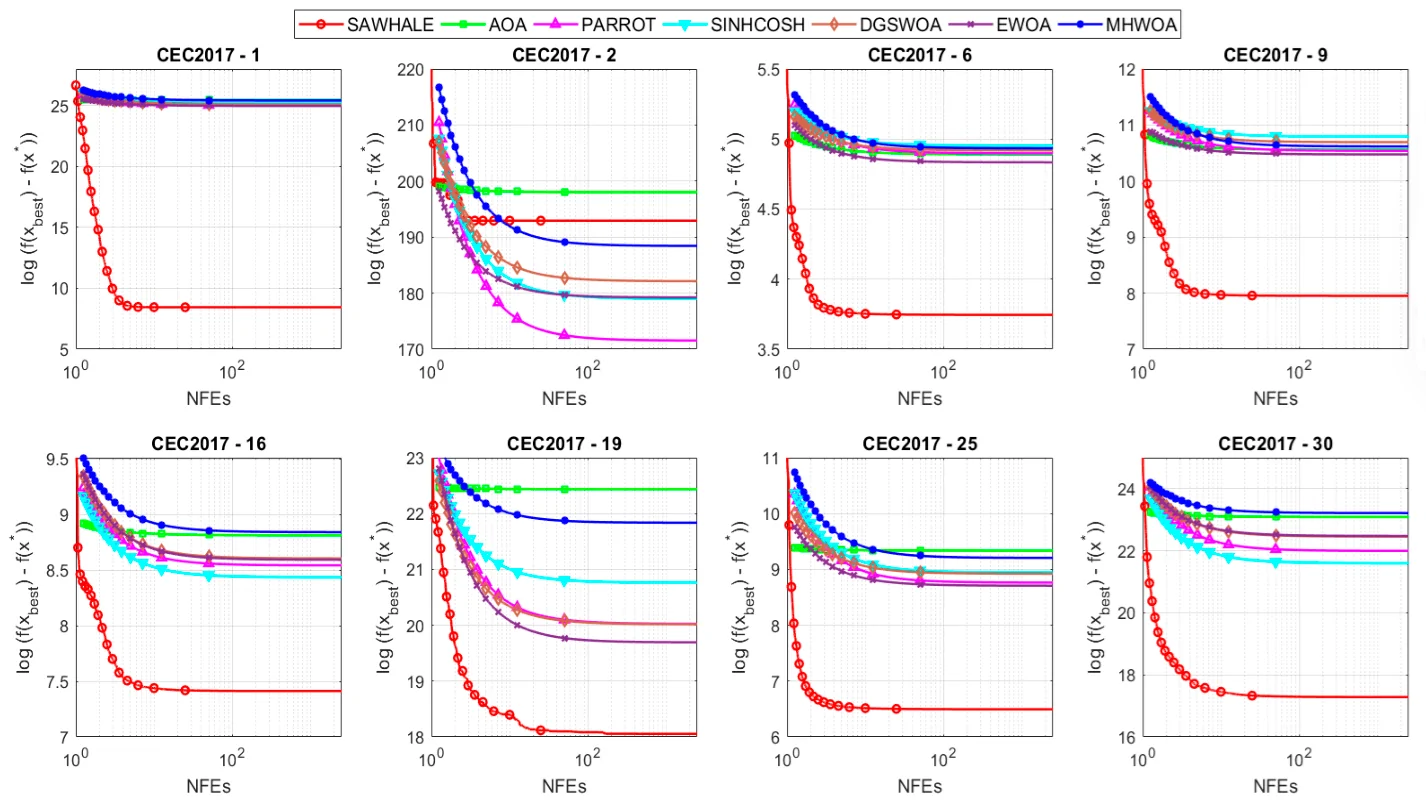
Convergence curves of the same seven algorithms on the same eight CEC 2017 problems at 50 dimensions.

Figure 11 and Figure 12 close the section with the convergence curves at 30 and at 50 dimensions on eight functions that cover the four families, namely F1 and F2 for the unimodal group, F6 and F9 for the multimodal group, F16 and F19 for the hybrids, and F25 and F30 for the compositions. Every curve counts exact evaluations only, and every curve terminates at the mean that Table 18 or Table 19 report. On the multimodal and composition panels, the SAWHALE curve leaves the six competitor curves early and keeps the lead to the end. On the F1 panels, the separation is the largest one in the whole study, which the seven orders of magnitude in the tables already promised. The F2 panels show the instability discussed above, with curves at astronomical error levels for every algorithm. On the hybrid panels, the EWOA curve runs closest to the SAWHALE curve and finishes below it on some functions at 30 dimensions, exactly where Table 20 places the losses. Taken as a whole, the CEC 2017 study confirms the CEC 2014 conclusions on an independent suite and against a different kind of opposition. The framework holds the first rank against recent metaheuristics and against newer variants of its own WHALE host. Its advantage lives on the multimodal and composition cores, and its one recurring soft spot stays on the hybrid functions.

### 6.8. Application of SAWHALE over Real-World Engineering Problems

**Figure 13:**
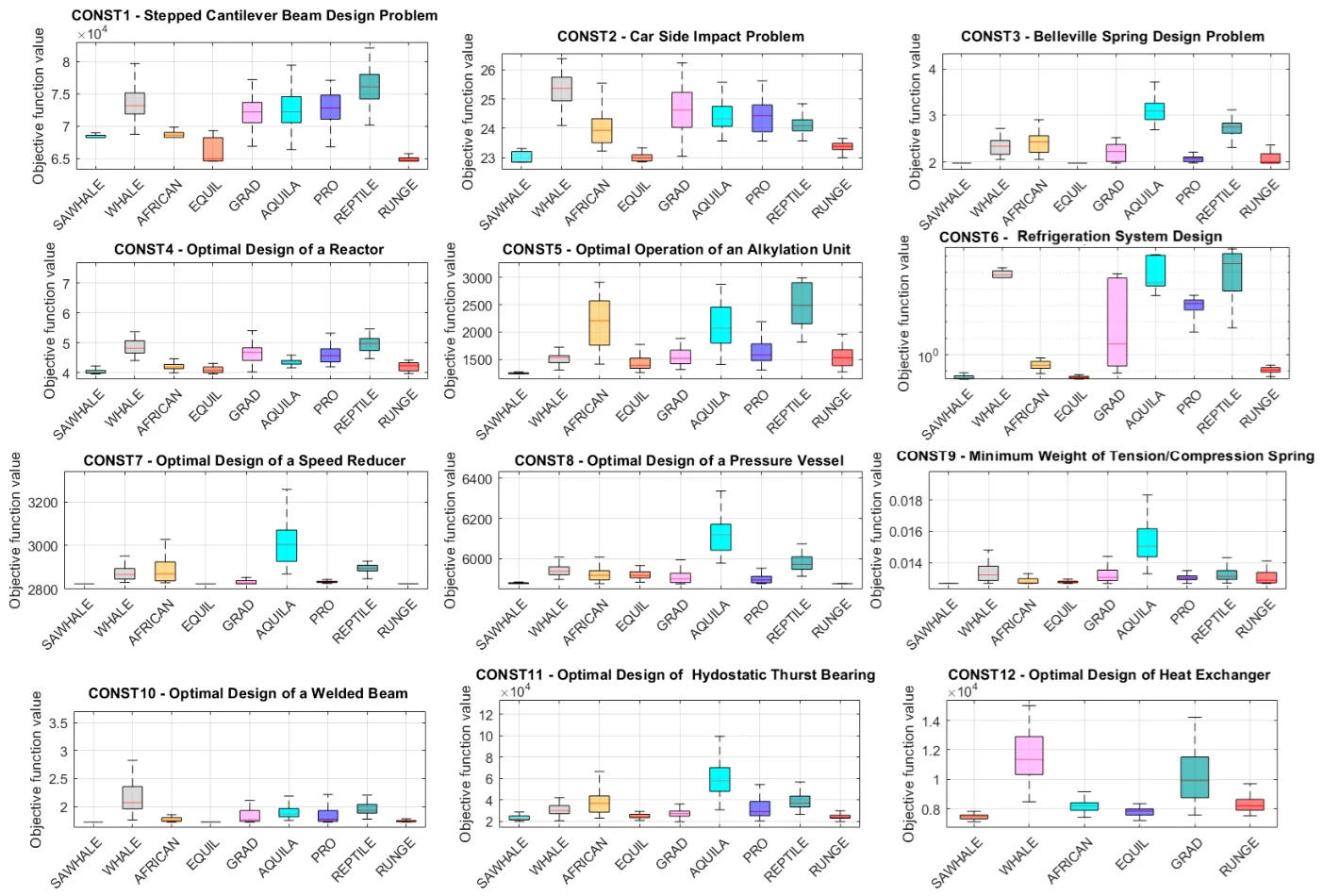
Representative boxplots visualizing the statistical analysis of employed algorithms for each engineering design problem.

This section analyzes the performance of the proposed SAWHALE algorithm on a test suite of twelve complex constrained engineering design cases. The suite covers stepped cantilever beam design (CONST1) [64], car side impact problem (CONST2) [65], Belleville spring design problem (CONST3) [64], optimal reactor design (CONST4) [66], optimal alkylation unit operation (CONST5) [66], optimal refrigeration system design (CONST6) [67], optimal speed reducer design (CONST7) [68], optimal pressure vessel design (CONST8) [69], minimum weight of tension/compression spring (CONST9) [70], optimal welded beam design (CONST10) [71], optimal hydrostatic thrust bearing design (CONST11) [72], and optimal heat exchanger design (CONST12) [66]. The problems vary in their functional characteristics, and their exact formulations are taken from Turgut and Turgut [66]. The detailed formulations are not repeated here for reasons of space. Interested readers can find more information about these test problems in the references provided. Table 21 reports the definitions of these design problems and their associated numbers of problem dimensions (D), inequality constraints (*g*(*x*)), equality constraints (*h*(*x*)), and the predetermined number of function evaluations (NFE) at which the run terminates. Predictions for these problems obtained by SAWHALE are compared with those of the standard WHALE and seven recent optimizers, namely the African Vultures Optimization (AFRICAN) [73], Equilibrium Optimizer (EQUIL) [74], Gradient-based Optimizer (GRAD) [75], Aquila Optimizer (AQUILA) [76], Poor and Rich Optimization (PRO) [77], Reptile Search (REPTILE) [78], and Runge–Kutta Optimizer (RUNGE) [79]. A statistical analysis is performed on 30 feasible solutions drawn from 50 independent runs of each method. Among alternative constraint-handling strategies, including the static penalty method, stochastic ranking, and feasibility and dominance rules, the problem’s design constraints are handled by a simple yet effective strategy developed by Kim et al. [80]. Table 22 and Table 23 list the algorithms that deliver the best feasible predictions for each engineering design problem. These two tables present the optimal values of the decision variables, the corresponding values of the defined problem constraints, and the minimum objective function values. The SAWHALE algorithm yields the most accurate predictions among the compared methods for most of the constrained test cases; it is outperformed only by EQUIL on a single case and by GRAD on the CONST8 and CONST11 cases. SAWHALE finds the same optimal solution as EQUIL and GRAD on the CONST7 problem, and the same solution as EQUIL on the CONST10 problem. Figure 13 presents a boxplot-based statistical analysis of the compared algorithms for each engineering design problem. The accuracy and robustness of the feasible solutions found by SAWHALE stand out for CONST1, CONST3, CONST5, CONST6, CONST7, CONST8, CONST9, and CONST10 problems since the collected feasible solutions accumulate within a very narrow deviation range, which produces a flat boxplot for SAWHALE on the associated problem. EQUIL is the second-best performer overall, while REPTILE yields the worst estimates for the mean. Integrating the local and global search schemes into the standard WHALE algorithm thus considerably enhances the probing capability of the search agents.

### 6.9. Binary SAWHALE Algorithm for Feature Selection

This section evaluates the proposed SAWHALE algorithm on feature selection. The continuous design variables are transformed into binary parameters, and the algorithm searches for the feature subset that minimizes the defined objective function. Feature selection datasets retrieved from publicly available sources are used to assess the binary algorithm’s predictive performance. Feature selection is a pre-processing step that identifies and removes unnecessary or redundant attributes from the data. Selecting the best possible feature subset reduces the problem’s dimensionality and the average computational run time, and it often improves the classification accuracy as well. The binary SAWHALE algorithm is a wrapper-based metaheuristic approach that scores each candidate subset with a kNN classifier. Across the literature, optimizing the feature subset involves two complementary tasks, which are maximizing prediction accuracy and minimizing the number of selected features. This study adopts the following objective function that minimizes the weighted summation of the error rate and the ratio of the number of selected features to the total number of features:

(52) $$f_{obj}=\alpha \cdot ERR+\beta \cdot \frac{\left\lceil SF\right\rceil }{\left|TF\right|}$$

where α∈(0,1) and β=1−α denote the weighting coefficients assigned to the two contributing objectives. *ERR* stands for the prediction error rate (ERR = 1 − Accuracy), which the kNN classifier evaluates. Each dataset is split independently into training and test sets through k-fold cross-validation. Here, $\left|SF\right|$ gives the number of selected features while $\left|TF\right|\;$is the total number of features in the employed dataset. Previous studies have assigned different weights to the two contributing objectives, α and β This study gives priority to minimizing the error rate over minimizing the number of selected features, since a higher classification accuracy is the primary goal, and the model parameters α and β are set to 0.90 and 0.10, respectively.

Feature selection is a discrete binary problem, so the continuous variables must be mapped into the binary space of zero and one. The literature on feature selection consistently applies two classes of transfer functions, namely S-shaped and V-shaped ones. This study adopts the sigmoid transfer function, a member of the S-shaped class, which converts the design variables’ actual values into probabilistic intervals between zero and one, as follows.

(53) $$C\left(X_{i}\right)=\frac{1}{1+e^{-X_{i}}}$$

where *X_i_* denotes the ith candidate solution of the population. Once the continuous variables are mapped to binary ones, the trial candidates take their binary form through the procedure below.

(54) $$X_{i}=\left\{\begin{array}{c} 0\;if\;{rand}_{1}\left(0,1\right)<C\left(X_{i}\right)\\ 1\;otherwise \end{array}\right.$$

where ${rand}_{1}\left(0,1\right)$ stands for a uniform random number between 0 and 1. All numerical experiments are run independently for a fair statistical evaluation. The k-NN classifier uses the five nearest neighbors (k = 5).

#### Numerical Experiments over Feature Selection Problems

This section evaluates the effectiveness of the proposed binary SAWHALE algorithm for feature selection on 18 datasets selected from the UCI machine learning repository [81], as shown in Table 24. The binary version of SAWHALE seeks the most relevant features among the available variables for the most accurate classification. The datasets differ in their numbers of instances and attributes, which makes them suitable cases for assessing the performance of the proposed surrogate-assisted WHALE optimization algorithm. Missing values and outliers in the considered cases have been amended, and the structural characteristics of the employed data sets have been re-evaluated and restructured, whether the features are continuous or categorical, and non-numeric or missing entries have been converted into usable numerical values. The 10-fold cross-validation technique is used to train and test the employed algorithms. The training folds serve to minimize the accumulated error rate through iterative adjustment of the decision variables, while the held-out folds measure the accuracy of the trained selector.

The classification performance of the compared optimizers is assessed with standard evaluation measures. The essential components of the confusion matrix, namely True-Positive (TP), True-Negative (TN), False-Negative (FN), and False-Positive (FP) predictions, have been used to develop accuracy evaluation metrics. These metrics quantify the correct classification rate, which is calculated as follows.

(55) $$Accuracy=\frac{TP+TN}{TP+FN+FP+TN}$$

In this context, the classification error rate is defined as follows.

(56) ERR=1−Accuracy

The average accuracy of each optimizer is computed over 30 independent runs and reads as follows.

(57) $${Accuracy}_{mean}=\frac{1}{N_{run}}\sum_{k=1}^{N_{run}}{Accuracy}_{best}^{k}$$

where *N_run_* = 30 counts the algorithm runs. The objective function is the weighted contribution of the error rate, and the selected number of features defined in Equation (52) is averaged over 30 algorithm runs and calculated by

(58) $${fitness}_{mean}=\frac{1}{N_{run}}\sum_{k=1}^{N_{run}}{fitness}_{best}^{k}$$

**Figure 14:**
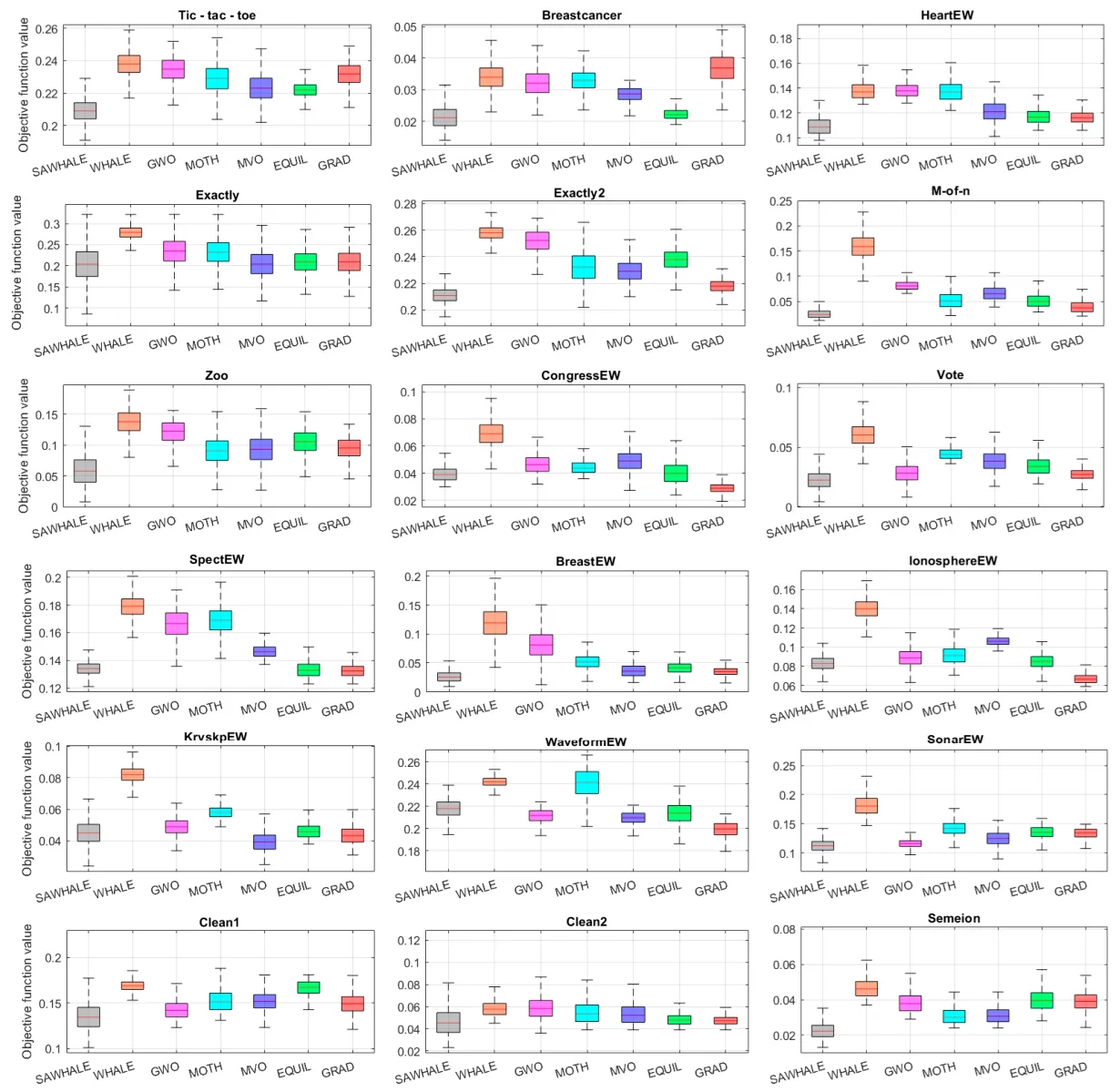
Statistical results obtained after 30 independent algorithms run for 18 feature selection problems.

**Figure 15:**
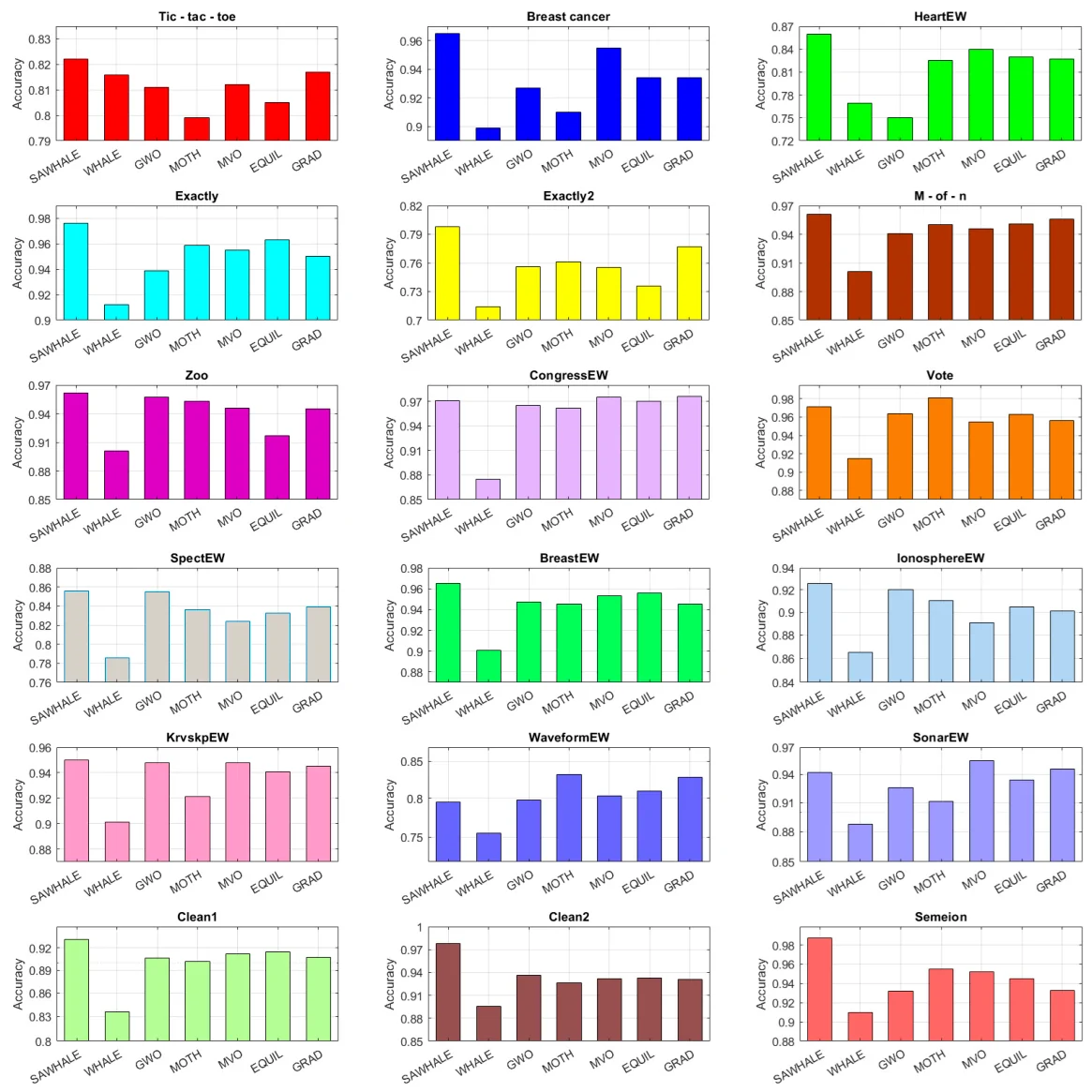
Average accuracy of the predictions performed by the compared optimizers for different feature selection problems.

**Figure 16:**
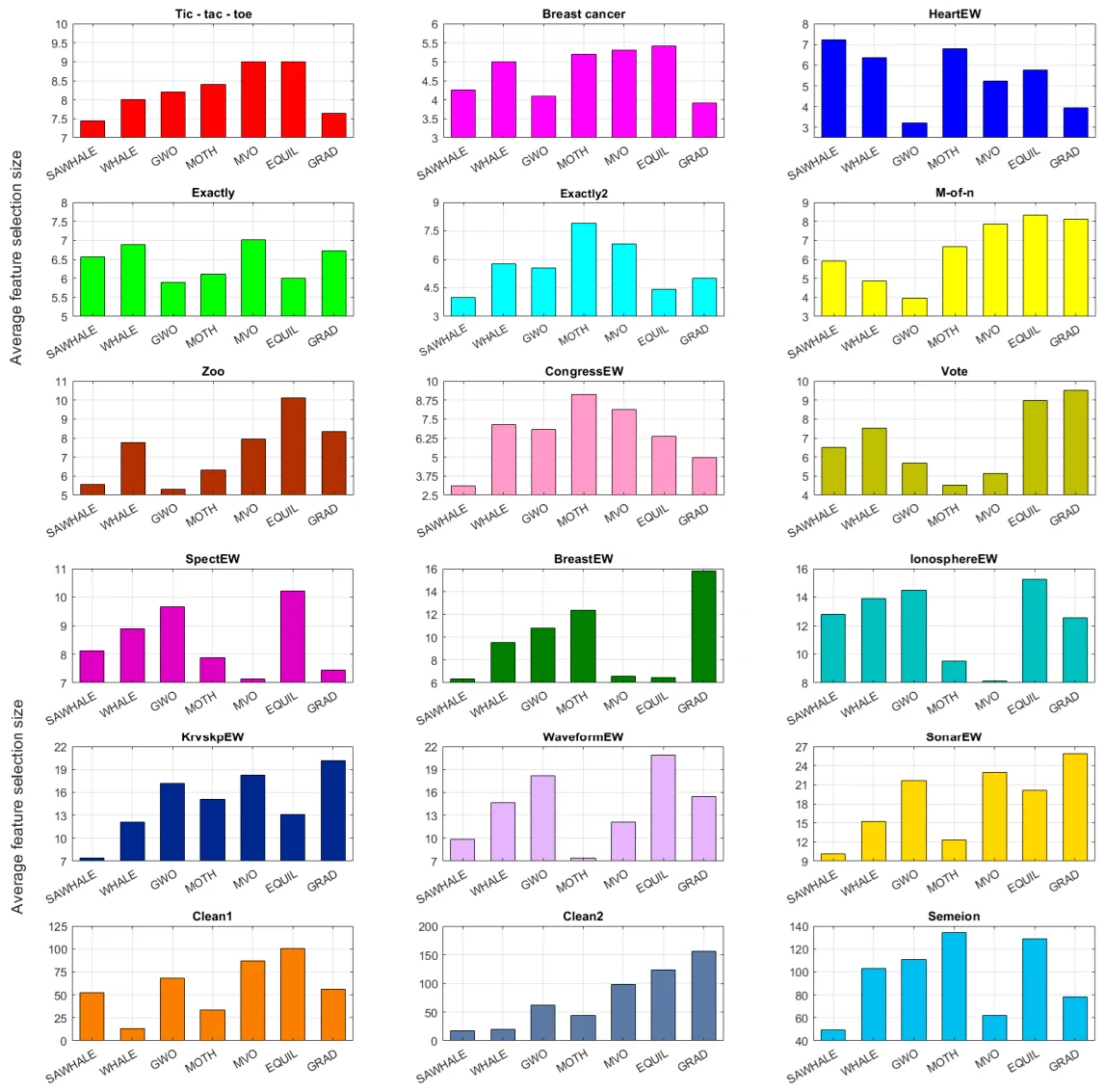
Average feature selection size obtained for competing algorithms.

Statistical analyses are also performed on the minimum, maximum, and standard deviation results of the accumulated objective function values across the datasets for the binary metaheuristic algorithms used to benchmark the proposed binary SAWHALE. The comparison set includes the original WHALE, Grey Wolf Optimization (GWO) [82], Moth-flame Optimizer (MOTH) [83], Multi-Verse Optimizer (MVO) [84], EQUIL, and GRAD. All algorithms are run 30 times, with 2000 function evaluations per run. Figure 14 presents the statistical results for the objective function values across data sets and test functions in boxplot form. On the best fitness values, the binary SAWHALE is the best-performing optimizer on 13 of the 18 datasets. It also leads the remaining methods on the mean results, providing the most robust predictions for 12 of 18 datasets. The superiority of the binary SAWHALE is also verified by the Friedman analysis, in which the algorithm attains the minimum mean rank of 1.55, followed by GRAD (3.16), EQUIL (3.50), MVO (3.55), MOTH (4.72), GWO (4.83), and WHALE (6.67). For the worst results, binary SAWHALE ranks third, behind GRAD in first place and EQUIL in second. The Wilcoxon rank-sum test is performed between the binary SAWHALE and the compared optimizers based on mean fitness values. The respective results are reported in Table 25, with a threshold value of *p* = 0.05. Values below this threshold indicate that the compared method and the binary SAWHALE differ significantly. The *p*-values reported in Table 25 support the conclusion that the proposed method is superior to the other remaining optimizers. Figure 15 compares the performance of the employed optimizers for the classification accuracy measure across 18 data sets. The comparative analysis of the bar plots reveals that the binary SAWHALE yields the most accurate predictions among the competing algorithms, with the fewest erroneous results across 14 datasets. In terms of mean prediction accuracy, SAWHALE is followed by the GRAD, MVO, EQUIL, GWO, MOTH, and standard WHALE algorithms. Figure 16 shows the average number of selected features per dataset and algorithm after 30 consecutive runs. The number of selected features is critical in designing the objective function, and binary SAWHALE selects the smallest subsets in seven cases, which is a competitive outcome.

## 7. Conclusions

This work sets out to make expensive optimization tractable for a Whale Optimization-based search without resting the burden on any single mechanism. The proposed SAWHALE framework combines two new asymmetric opposition operators, EDOFAS and OPADAMP, with surrogate-assisted candidate pricing in both search phases and with a logistic rule that switches between the phases under stagnation. Every design decision is tested, and the conclusions below repeat only what the experiments measured.

The component study isolates the two operators inside an otherwise unchanged host. EDOFAS holds the first Friedman rank among nine variants at both 500 and 1000 dimensions, at 1.650 and 1.575, and stays finite on the two overflow-prone objectives that eliminate six of the nine variants. OPADAMP is almost never harmful and wins exactly where progress depends on perturbing the worst coordinates. The two operators, therefore, play complementary roles, which is the property the full framework exploits.

Against five recent surrogate-assisted optimizers on the CEC 2014 suite, the complete framework obtains the best mean rank at both dimensions, 2.233 at 30 and 2.267 at 50, and records more Wilcoxon wins than losses against every competitor. The ablation then attaches a measured price to each module. The full design ranks first at 1.333 and 2.033 over seven configurations. Removing the surrogate guidance is the most expensive single cut, and conventional opposition buys almost nothing over no opposition at all, so the benefit comes from the proposed operators themselves. Against six recent metaheuristics on the CEC 2017 suite, including three newer whale optimization algorithm variants, the framework again ranks first at 1.767 and 1.600. The constrained engineering problems and the binary feature selection study extend the same behavior to real models of continuous and discrete character.

The limits are stated as plainly as the gains. The machinery has a real cost on smooth unimodal ground, where the leanest ablated variants win, and one hybrid competitor stays close on the hybrid functions of the CEC 2017 suite. Future work will follow three directions. The first is an adaptive choice among several surrogate families instead of a single fixed model. The second is an extension of the asymmetric opposition operators to constrained and multi-objective settings, where the geometry of the feasible region changes what a useful opposite is. The third is a sharper binary transfer rule, since the feature selection results suggest that the continuous advantages survive binarization only partially. Each of these directions can reuse the evaluation protocol of this paper, and each will be judged by the same standard.

## Figures and Tables

**Table 1:** Mechanism-level comparison of the proposed framework with the most closely related studies.

Study	Base Algorithm	Surrogate Model(s)	Opposition Mechanism	Phase-Specific Opposition	Adaptive Switching Signal	Application Scope
Wang et al. [17]	WHALE	Local RBF	none	-	none	fractional chaotic parameter identification
Chen et al. [13]	WHALE	none	Chaotic quasi opposition	no (uniform)	none	engineering design
Xing et al. [14]	WHALE	none	Quasi-opposition + Gaussian bare-bones	no (uniform)	none	feature selection, image segmentation
Xuan et al. [15]	WHALE	none	quasi-opposition + nonlinear factor	no (uniform)	none	cloud task scheduling
Long et al. [16]	WHALE	none	refraction-based OBL	no (uniform)	none	high-dimensional problems, PV parameters
Wang et al. [24] (GOPSO)	PSO	none	generalized opposition (random reflection point)	no (uniform)	none	rotated multimodal, shifted large-scale
Sun et al. [27] (TLSAPSO)	PSO	global + local surrogates	none	-	fixed hierarchy	expensive benchmarks
Chen et al. [28]	WCA	hierarchical surrogates	none	-	fixed hierarchy	high-dimensional expensive
Chen et al. [30]	DE	two-stage RBF	none	-	staged	high-dimensional expensive
Ling [26]	TLBO	hierarchical RBF	two OBL variants (mutual opposition)	no (global only)	fixed hierarchy	high-dimensional benchmarks
This work	WHALE	fitness inheritance (global) + adaptive GRNN (local)	EDOFAS (global) + OPADAMP (local), both asymmetric	yes	logistic stagnation gate + entropy/diversity scores	CEC suites, engineering design, feature selection

**Table 2:** Experimental settings and reported outcomes of the surrogate-assisted comparators and the closest opposition-based precedents.

Study	Base Algorithm	Opposition Mechanism	Surrogate Model	Test Problems	Dim	Budget (FEs)	Reported Outcome
Huang et al. [31] (ESPSO)	PSO with evolutionary sampling, local and trust-region search	none	adaptive Gaussian-kernel RBF using personal-best data	six functions (18 dimension cases)	30/50/100	1000	best Friedman rank (2.194); significant vs. 3/5 comparators; weaker on very complex multimodal functions
Cheng et al. [32] (MSASFS)	SFS with surrogate-assisted DE updating	none	cubic RBF + GP (Matérn 3/2; EI screening)	six standard; nine additional, mainly CEC2017; three chaotic systems	30/50/100/200; extra: 100; apps: 3/3/9	benchmarks: 1000; apps: 100 iterations, *n* = 40	2nd at 30D; joint 1st at 50D; 1st at 100/200D; best on 5/9 additional functions; superior on all three applications
Li et al. [33] (GPE-QI)	GPE with EGM (1,1) population prediction	none; TOGPE only as comparator	quadratic-interpolation surrogate (QISM)	CEC2019 (10); CEC2020 (10); six engineering designs	CEC2019: 9/10/16/18; CEC2020: 15/20; eng.: 2/3/4/7	CEC2019: 10,000·D; CEC2020: 3M/10M; eng.: iteration caps (actual 420–47,190)	significantly better than 6/7 comparators; no significant difference vs. LSHADE-cnEpSin; engineering best values equal or better than literature minima
Zhou et al. [34] (NRO)	(N+1)-ES-like population search	none	local linear regression predicting a descent direction	CEC2014 (24); CEC2015 (15)	CEC2014: 10/20/30; CEC2015: 10/30	50·D	competitive or superior in most cases; clear advantage on unimodal, smooth functions
Wang et al. [17] (ISAWOA)	WHALE with Lévy flight and quadratic interpolation	none; OBWOA only as comparator	local cubic RBF for candidate pre-screening	20 classical functions; four fractional chaotic systems	30/50/100; apps: 3 variables	no fixed FE cap; 500/100 iterations; exact-evaluation rate 77–97.91%	first benchmark rank; best accuracy on 3/4 applications; not fastest in every application
Wang et al. [24] (GOPSO/DP-GOPSO)	PSO with Cauchy mutation; DP variant adapts population size	GOBL at initialization and generation jumping (p_0_ = 0.3)	none	twelve classical functions; six shifted CEC2008 functions	30; large-scale: 100	200,000; large-scale: 5000·D = 500,000	GOPSO best rank at 30D; DP-GOPSO 2nd at 100D; GOBL performed poorly on shifted/large-scale problems

**Table 3:** Nomenclature of the proposed operators and framework.

Symbol	Description	Defined in
N, D	population size; problem dimension	—
$FES,\;FES_{max}$	consumed and maximum exact function evaluations	—
$\hat{t}=FES/FES_{max}$	budget progress	Section 5.1
$c\left(s\right)$	reflection center of the scaled opposition rule	Equation (13)
$S_{base}=\{{\bar{s}}_{1},{\bar{s}}_{2},{\bar{s}}_{3}\}$	base scale set {0.2,0.5,1.0}	Section 4.2.3
K	scales probed per parent (K=3)	Section 4.2.3
$f_{i}^{norm}$	normalized fitness of individual i	Equation (14)
$n_{bins}$	fitness histogram bins (10)	Equation (15)
$p_{b}$	probability of bin b	Equation (16)
$H,\;H_{n}$	population fitness entropy; normalized entropy	Equations (17) and (18)
$r_{imp}$	relative improvement rate	Equation (19)
$E_{expl},\;E_{expt}$	exploration/exploitation scores	Equations (20), (21), (42) and (43)
γ	mode-gate margin (0.5)	Equations (22) and (44)
λ	entropy-modulation gain (1)	Equation (23)
$s_{k}\left(t\right)$	k-th opposition scale at progress $\hat{t}$	Equation (23)
$I_{mid},\;\rho_{sel}$	middle-ranked opposed band; its width (0.5)	Equation (26)
$d_{i}^{min}$	nearest-subsample distance (density score)	Equation (27)
$\delta_{i},\;∥\delta {∥}_{max}$	last displacement of individual i; its largest magnitude	Equations (29) and (30)
$E,\;k_{elite}$	elite index set; elite count (N/2)	Equation (31)
$s_{t},\;\varphi$	OPADAMP amplitude; its base (0.5)	Equation (32)
$w_{i},\;u_{i},\;z_{i}$	fitness, displacement, combined weights	Equations (33)–(35)
${\overset{¯}{x}}_{E}$	elite centroid	Equation (36)
$n_{cand},\;\beta$	candidates per elite (8); perturbation fraction (0.05)	Equation (39)
$Div_{pop},\;Div_{arch}$	population/archive diversity	Equations (40) and (46)
$Div_{max}$	running maximum of archive diversity	Equation (47)
A, |A|	archive of exactly evaluated points; capacity (5N)	Section 5.3
σ	GRNN smoothing bandwidth	Equations (12) and (51)
$k_{pred},\;k_{SCI}$	k-NN sizes for prediction (20) and for the SCI (10)	Section 4.4, Equation (48)
$W_{i},\;SCI$	kernel mass of archive point i; separation coverage index	Equations (48) and (49)
$\tau_{t},\;\alpha$	adaptive SCI target; bandwidth adaptation rate (0.05)	Equations (50) and (51)
$s\left(t\right),\;\tau$	stagnation counter; switching threshold (3)	Equation (45)

— means not available for this entity.

**Table 4:** Internal control parameters of SAWHALE (fixed across all experiments).

Parameter	Symbol	Value	Role
Scales per parent	K	3	Equation (23)
Base scale set	$S_{base}$	{0.2,0.5,1.0}	Equation (23)
Entropy gain	λ	1	Equation (23)
Opposed bandwidth	$\rho_{sel}$	0.5	Equation (26)
Fitness bins	$n_{bins}$	10	Equations (15) and (16)
Mode-gate margin	γ	0.5	Equations (22) and (44)
Inheritance references	M	4	Equation (9)
Archive capacity	|A|	5N	Section 5.3
Stagnation threshold	τ	3	Equation (45)
OPADAMP amplitude base	φ	0.5	Equation (32)
Elite count	$k_{elite}$	N/2	Equation (31)
Candidates per elite	$n_{cand}$	8	Equation (39)
Perturbation fraction	β	0.05	Equation (39)
GRNN prediction neighbors	$k_{pred}$	20	Section 4.4
SCI neighbors	$k_{SCI}$	10	Equation (48)
Bandwidth adaptation rate	α	0.05	Equation (51)
SCI target range	$\left[\tau_{min},\tau_{max}\right]$	$\left[0.05,0.25\right]$	Equation (50)
Bandwidth bounds	—	$\left[10^{-3},10\right]$	Equation (51)
Initial bandwidth	$\sigma \left(0\right)$	1	Equation (51)

**Table 5:** Per-iteration cost of the framework components.

Component	Time	Memory	Exact FEs
WHALE sweep, Equations (1)–(6)	$O\left(ND\right)$	—	N
Entropy, Equations (14)–(18)	$O\left(N+n_{bins}\right)$	$O\left(n_{bins}\right)$	0
Opposite generation, Equations (24) and (25)	$O\left(MKD\right)$	$O\left(MKD\right)$	0
Exact-mode scoring	$O\left(MK\right)$ calls	—	MK≤1.5N
Inheritance pricing, Equations (9) and (10)	$O\left(4\;MKD\right)$	—	0
Survivor selection, Equations (27) and (28)	$O\left(n_{comb}^{2}D\right)\leq O\left(6.25\;N^{2}D\right)$	$O\left(n_{comb}D\right)$	≤N
OPADAMP, Equations (29)–(38)	$O\left(ND\right)$	$O\left(ND\right)$	0
GRNN refinement, Section 4.4	$O\left(k_{elite}\;n_{cand}\;|A|\;D\right)\leq O\left(20\;N^{2}D\right)$	$O\left(|A|D\right)$	0
Bandwidth update, Equations (46)–(51)	$O\left(|A{|}^{2}D\right)\leq O\left(25\;N^{2}D\right)$	$O\left(|A|D\right)$	0
Archive maintenance	$O\left(1\right)$ amortized append	$O\left(|A|D\right)=O\left(ND\right)$	—

— means not available for this entity.

**Table 6:** Statistical results of the nine WHALE variants on the 500-dimensional shifted test problems F1–F10 (mean ± standard deviation of the final objective error over 30 runs).

**Algorithm**	**F1—Rosenbrock**	**F2—Gen. Dixon–Price**	**F3—Sphere**	**F4—Schwefel 2.21**	**F5—Sum Diff. Powers**
WHALE-OPADAMP	1.8504E-01 ± 1.8504E-01	0.0000E+00 ± 0.0000E+00	7.1052E-03 ± 1.4022E-02	1.9310E-03 ± 5.6247E-03	2.5667E-06 ± 9.2464E-06
WHALE-EDOFAS	1.6325E-02 ± 1.6325E-02	0.0000E+00 ± 0.0000E+00	7.2627E-04 ± 1.1574E-03	4.1375E-03 ± 3.1275E-03	3.3333E-07 ± 8.0230E-07
WHALE-OBL	9.9160E+00 ± 9.9160E+00	0.0000E+00 ± 0.0000E+00	1.2550E-01 ± 1.7623E-01	4.3190E-02 ± 5.4125E-02	NaN
WHALE-QOBL	4.9590E+02 ± 4.9590E+02	1.8646E+02 ± 4.7223E+01	1.2155E+02 ± 5.6525E+01	7.9769E-01 ± 6.6643E-01	NaN
WHALE-COBL	2.9717E+02 ± 2.9717E+02	0.0000E+00 ± 0.0000E+00	6.2346E-02 ± 8.4257E-02	2.5802E-02 ± 1.7389E-02	NaN
WHALE-DOBL	4.9612E+02 ± 4.9612E+02	2.1704E+02 ± 3.1266E+01	3.1167E+02 ± 1.1203E+02	7.0296E-01 ± 8.1990E-01	NaN
WHALE-EOBL	3.3779E+02 ± 3.3779E+02	9.9003E-03 ± 5.0124E-02	4.1800E+00 ± 2.1480E+00	1.5023E-01 ± 1.5378E-01	NaN
WHALE-GOBL	4.9407E+02 ± 4.9407E+02	8.9922E+01 ± 2.9496E+01	1.6785E+02 ± 2.7925E+01	4.9944E+00 ± 1.6865E+00	7.9256E-01 ± 7.7893E-01
WHALE	7.8409E-01 ± 7.8409E-01	0.0000E+00 ± 0.0000E+00	9.8710E-02 ± 1.1036E-01	6.0208E-02 ± 5.3654E-02	NaN
**Algorithm**	**F6** **—Quadratic**	**F7—Bent Cigar**	**F8—Schwefel 2.22**	**F9—Zakharov**	**F10—Sum of Squares**
WHALE-OPADAMP	5.0181E+00 ± 1.4006E+01	1.5050E+04 ± 2.8143E+04	1.8426E+00 ± 2.1506E+00	2.9388E+05 ± 1.7117E+05	2.0914E+00 ± 5.7383E+00
WHALE-EDOFAS	1.0827E-01 ± 1.4296E-01	9.2656E+02 ± 1.1364E+03	2.7826E-01 ± 2.1140E-01	3.6925E+05 ± 1.1289E+05	1.8611E-01 ± 2.1491E-01
WHALE-OBL	5.9158E+01 ± 5.4802E+01	1.8395E+05 ± 2.3315E+05	8.1692E+00 ± 6.5748E+00	4.0912E+05 ± 9.2296E+04	4.9794E+01 ± 5.9748E+01
WHALE-QOBL	2.3055E+04 ± 1.5244E+04	1.6500E+08 ± 8.2934E+07	4.3499E+01 ± 1.9826E+01	1.3896E+05 ± 1.3971E+05	3.5047E+04 ± 1.7476E+04
WHALE-COBL	4.1252E+01 ± 5.7857E+01	8.8004E+04 ± 1.3378E+05	1.2562E+00 ± 1.3571E+00	2.1006E+05 ± 1.1730E+05	1.6543E+01 ± 2.2643E+01
WHALE-DOBL	9.2078E+04 ± 5.8876E+04	3.8469E+08 ± 1.9723E+08	7.8492E+01 ± 3.1507E+01	7.0912E+04 ± 3.5233E+04	8.9717E+04 ± 4.4784E+04
WHALE-EOBL	1.9984E+03 ± 1.2915E+03	6.5653E+06 ± 3.5912E+06	2.3698E+01 ± 1.2214E+01	1.8660E+05 ± 1.2687E+05	1.7243E+03 ± 9.0112E+02
WHALE-GOBL	1.7408E+05 ± 5.7808E+04	3.2983E+08 ± 7.6402E+07	2.0877E+02 ± 7.9369E+01	4.1274E+05 ± 1.6128E+04	7.2169E+04 ± 1.7840E+04
WHALE	8.0390E+01 ± 1.0794E+02	1.1838E+05 ± 1.3719E+05	1.2757E+01 ± 1.2820E+01	4.0781E+05 ± 5.6867E+04	4.8183E+01 ± 7.5252E+01

*NaN denotes a non-finite mean caused by floating-point overflow of the objective (the high-order power sum of F5 and, at D = 500, the coordinate product of F8); non-finite entries were assigned the worst rank in all statistical comparisons.*

**Table 7:** Statistical results of the nine WHALE variants on the 500-dimensional shifted test problems F11–F20 (mean ± standard deviation over 30 runs).

**Algorithm**	**F11—Trigonometric**	**F12—Rastrigin**	**F13—Griewank**	**F14—Schaffer F6**	**F15—HGBat**
WHALE-OPADAMP	1.0015E+00 ± 2.6023E-03	2.5036E+00 ± 5.4871E+00	6.9333E-05 ± 1.0680E-04	3.4521E+01 ± 3.3221E+01	5.8568E-01 ± 2.1961E-01
WHALE-EDOFAS	1.0004E+00 ± 4.9836E-04	2.3461E-01 ± 4.1275E-01	4.8667E-06 ± 5.4818E-06	2.1164E+01 ± 1.1339E+01	5.5580E-01 ± 3.6353E-01
WHALE-OBL	2.0378E+00 ± 4.2907E+00	7.9493E+01 ± 1.0408E+02	1.2024E-03 ± 1.8253E-03	8.9095E+01 ± 3.3893E+01	4.3313E-01 ± 3.2756E-01
WHALE-QOBL	1.1712E+03 ± 9.1370E+01	1.9930E+03 ± 8.4009E-01	4.4142E-01 ± 1.6169E-01	6.8520E+02 ± 1.4347E+02	1.7022E+01 ± 5.0004E+01
WHALE-COBL	6.2790E+00 ± 1.4594E+01	1.1120E+02 ± 1.3628E+02	9.7070E-04 ± 1.1824E-03	8.9272E+01 ± 3.4819E+01	2.7828E-01 ± 2.0385E-01
WHALE-DOBL	1.1966E+03 ± 9.0129E+01	1.9943E+03 ± 1.0350E+00	8.6084E-01 ± 2.4955E-01	9.2030E+02 ± 1.5382E+02	2.7843E+02 ± 2.3285E+02
WHALE-EOBL	1.1499E+02 ± 1.6682E+02	5.4024E+02 ± 4.5368E+02	4.2585E-02 ± 3.1904E-02	3.1886E+02 ± 8.0063E+01	3.3614E-01 ± 2.3138E-01
WHALE-GOBL	7.9768E+02 ± 1.7885E+02	1.9912E+03 ± 3.5636E-01	7.6968E-01 ± 1.0008E-01	7.9393E+02 ± 4.3432E+01	2.1715E+02 ± 1.1067E+02
WHALE	1.8084E+00 ± 3.6126E+00	5.5721E+01 ± 8.9666E+01	1.8988E-03 ± 2.3975E-03	1.0736E+02 ± 4.4108E+01	4.5899E-01 ± 2.8377E-01
**Algorithm**	**F16—Alpine**	**F17—Ackley**	**F18—Penalized P1**	**F19—Schaffer F7**	**F20—Defl. Corr. Spring**
WHALE-OPADAMP	8.7518E-02 ± 1.9656E-01	1.1935E-02 ± 1.9053E-02	2.6333E-06 ± 3.9522E-06	4.1633E+00 ± 6.6607E+00	7.4998E-02 ± 7.7859E-02
WHALE-EDOFAS	1.0296E-02 ± 8.3710E-03	8.2409E-03 ± 9.8769E-03	4.3333E-07 ± 8.9763E-07	1.8609E+00 ± 2.5200E+00	1.4133E-01 ± 4.6783E-02
WHALE-OBL	6.1690E-01 ± 1.8319E+00	1.4466E-01 ± 1.7549E-01	8.8600E-05 ± 8.6952E-05	2.5788E+01 ± 2.2957E+01	4.4433E-01 ± 3.4890E-01
WHALE-QOBL	1.7851E+02 ± 1.0463E+02	2.1134E+00 ± 6.4549E-01	1.1375E-01 ± 7.7993E-02	1.4409E+03 ± 5.9493E+02	2.0729E+01 ± 7.9114E+00
WHALE-COBL	3.9803E-01 ± 4.3550E-01	1.8953E-02 ± 1.7043E-02	6.3567E-05 ± 6.8255E-05	2.0593E+01 ± 1.4581E+01	3.4465E-01 ± 2.3418E-01
WHALE-DOBL	3.4094E+02 ± 1.1822E+02	3.6545E+00 ± 1.1538E+00	8.4193E-01 ± 5.7601E-01	2.5104E+03 ± 7.8354E+02	4.3987E+01 ± 1.9535E+01
WHALE-EOBL	1.6732E+01 ± 1.8418E+01	8.3758E-01 ± 4.9416E-01	2.0411E-03 ± 1.3560E-03	2.1837E+02 ± 1.6595E+02	3.9267E+00 ± 3.3111E+00
WHALE-GOBL	3.5772E+02 ± 5.4998E+01	7.2844E-01 ± 1.3321E+00	5.3387E-01 ± 1.9167E-01	1.7639E+03 ± 2.2108E+02	7.6171E+01 ± 1.3906E+01
WHALE	2.0955E+00 ± 3.7179E+00	1.5723E-01 ± 1.1153E-01	7.2033E-05 ± 8.3149E-05	3.5227E+01 ± 3.6310E+01	4.9610E-01 ± 4.2571E-01

**Table 8:** Statistical results of the nine WHALE variants on the 1000-dimensional shifted test problems F1–F10 (mean ± standard deviation over 30 runs).

**Algorithm**	**F1—Rosenbrock**	**F2—Gen. Dixon–Price**	**F3—Sphere**	**F4—Schwefel 2.21**	**F5—Sum Diff. Powers**
WHALE-OPADAMP	3.5756E-01 ± 6.3185E-01	0.0000E+00 ± 0.0000E+00	9.1382E-03 ± 2.0195E-02	3.4236E-03 ± 5.8339E-03	4.4333E-06 ± 9.0084E-06
WHALE-EDOFAS	4.1644E-02 ± 5.0629E-02	0.0000E+00 ± 0.0000E+00	1.6293E-03 ± 2.4236E-03	4.8616E-03 ± 5.0781E-03	1.3000E-06 ± 2.2766E-06
WHALE-OBL	2.8210E+00 ± 4.8304E+00	0.0000E+00 ± 0.0000E+00	1.8697E-01 ± 2.0265E-01	6.9548E-02 ± 1.0519E-01	NaN
WHALE-QOBL	9.9377E+02 ± 8.4560E-01	4.4448E+02 ± 8.5300E+01	2.5480E+02 ± 1.1678E+02	5.1969E-01 ± 4.6736E-01	NaN
WHALE-COBL	3.9989E+02 ± 4.8930E+02	1.3333E-07 ± 7.3030E-07	1.9464E-01 ± 3.2530E-01	3.5500E-02 ± 2.4060E-02	NaN
WHALE-DOBL	9.9454E+02 ± 5.7441E-01	5.2105E+02 ± 7.5965E+01	6.5971E+02 ± 2.7321E+02	7.2955E-01 ± 8.1252E-01	NaN
WHALE-EOBL	6.6493E+02 ± 4.1540E+02	3.4530E-02 ± 1.6971E-01	1.1681E+01 ± 4.4395E+00	2.3935E-01 ± 2.0907E-01	NaN
WHALE-GOBL	9.9078E+02 ± 5.5894E-01	2.3048E+02 ± 4.4662E+01	4.9325E+02 ± 8.4463E+01	5.0045E+00 ± 1.4605E+00	1.4877E+00 ± 1.1739E+00
WHALE	2.3825E+00 ± 4.2352E+00	0.0000E+00 ± 0.0000E+00	2.0442E-01 ± 2.4544E-01	5.5988E-02 ± 8.5963E-02	NaN
**Algorithm**	**F6—Quadratic**	**F7—Bent Cigar**	**F8—Schwefel 2.22**	**F9—Zakharov**	**F10—Sum of Squares**
WHALE-OPADAMP	2.0656E+01 ± 6.0795E+01	2.2853E+04 ± 3.4893E+04	2.6886E+00 ± 5.3890E+00	7.5249E+05 ± 3.1682E+05	1.0737E+01 ± 1.7945E+01
WHALE-EDOFAS	9.8042E-01 ± 1.7648E+00	1.8504E+03 ± 5.7059E+03	6.8612E-01 ± 7.4064E-01	7.3201E+05 ± 2.2466E+05	8.1684E-01 ± 1.5411E+00
WHALE-OBL	2.1024E+02 ± 2.2135E+02	3.1518E+05 ± 3.1907E+05	NaN	8.3007E+05 ± 4.8300E+04	1.4401E+02 ± 1.9077E+02
WHALE-QOBL	1.5014E+05 ± 7.3184E+04	2.5887E+08 ± 1.3408E+08	NaN	4.9654E+05 ± 3.4208E+05	1.1105E+05 ± 6.1100E+04
WHALE-COBL	1.4402E+02 ± 2.0481E+02	2.5304E+05 ± 4.3943E+05	NaN	4.6137E+05 ± 2.3081E+05	1.1193E+02 ± 1.6100E+02
WHALE-DOBL	5.6366E+05 ± 3.5226E+05	9.0960E+08 ± 4.3493E+08	NaN	1.7128E+05 ± 1.3683E+05	4.2506E+05 ± 2.5649E+05
WHALE-EOBL	1.0210E+04 ± 6.4902E+03	1.5103E+07 ± 9.0893E+06	NaN	6.4754E+05 ± 3.1747E+05	7.6189E+03 ± 4.2808E+03
WHALE-GOBL	1.0488E+06 ± 2.3512E+05	9.8331E+08 ± 1.6724E+08	4.0553E+02 ± 1.8168E+02	8.1937E+05 ± 1.9963E+04	4.3044E+05 ± 1.1575E+05
WHALE	4.7665E+02 ± 4.8047E+02	6.0992E+05 ± 7.6128E+05	NaN	8.5498E+05 ± 1.2189E+05	2.1494E+02 ± 3.7689E+02

*NaN denotes a non-finite mean caused by floating-point overflow of the objective (the high-order power sum of F5 and, at D = 1000, the coordinate product of F8); non-finite entries were assigned the worst rank in all statistical comparisons.*

**Table 9:** Statistical results of the nine WHALE variants on the 1000-dimensional shifted test problems F11–F20 (mean ± standard deviation over 30 runs).

**Algorithm**	**F11—Trigonometric**	**F12—Rastrigin**	**F13—Griewank**	**F14—Schaffer F6**	**F15—HGBat**
WHALE-OPADAMP	1.0504E+00 ± 1.2075E-01	4.3163E+00 ± 8.9733E+00	1.1430E-04 ± 1.8367E-04	8.5682E+01 ± 6.1834E+01	6.5082E-01 ± 2.3083E-01
WHALE-EDOFAS	1.0011E+00 ± 1.7358E-03	6.2749E-01 ± 1.6393E+00	8.2667E-06 ± 9.4356E-06	5.1896E+01 ± 2.8219E+01	5.6538E-01 ± 3.5804E-01
WHALE-OBL	4.3525E+00 ± 1.1759E+01	1.2989E+02 ± 2.1333E+02	1.4260E-03 ± 2.7605E-03	1.5934E+02 ± 7.9575E+01	5.6522E-01 ± 3.8671E-01
WHALE-QOBL	2.3332E+03 ± 3.1365E+02	3.9879E+03 ± 1.7384E+00	6.0450E-01 ± 2.2043E-01	1.2485E+03 ± 2.6356E+02	7.4277E+01 ± 1.4363E+02
WHALE-COBL	7.4425E+00 ± 1.5044E+01	1.2087E+02 ± 2.1309E+02	7.3680E-04 ± 9.5708E-04	1.6866E+02 ± 5.9672E+01	3.5242E-01 ± 2.9481E-01
WHALE-DOBL	2.5374E+03 ± 1.7649E+02	3.9909E+03 ± 1.0502E+00	1.1371E+00 ± 2.2203E-01	1.7781E+03 ± 2.1709E+02	6.5472E+02 ± 4.3329E+02
WHALE-EOBL	2.3375E+02 ± 2.9165E+02	9.1494E+02 ± 5.5819E+02	4.6452E-02 ± 2.5634E-02	6.4211E+02 ± 1.3075E+02	3.9282E-01 ± 2.9511E-01
WHALE-GOBL	1.6685E+03 ± 3.4928E+02	3.9841E+03 ± 1.1801E+00	1.0858E+00 ± 1.2020E-01	1.6294E+03 ± 9.5895E+01	8.3585E+02 ± 2.0151E+02
WHALE	2.5585E+00 ± 5.8393E+00	7.4379E+01 ± 1.0181E+02	1.7568E-03 ± 2.4684E-03	1.8740E+02 ± 8.9395E+01	4.7995E-01 ± 3.1241E-01
**Algorithm**	**F16—Alpine**	**F17—Ackley**	**F18—Penalized P1**	**F19—Schaffer F7**	**F20—Defl. Corr. Spring**
WHALE-OPADAMP	1.2037E-01 ± 2.1823E-01	1.9847E-02 ± 2.8855E-02	1.8333E-06 ± 4.8855E-06	8.0799E+00 ± 1.7972E+01	6.3399E-02 ± 7.2788E-02
WHALE-EDOFAS	2.4387E-02 ± 2.4559E-02	9.1539E-03 ± 1.0630E-02	2.3333E-07 ± 6.2606E-07	2.3463E+00 ± 1.8344E+00	1.1672E-01 ± 6.7426E-02
WHALE-OBL	4.0408E-01 ± 3.2485E-01	1.5510E-01 ± 1.3758E-01	8.9967E-05 ± 1.3869E-04	4.2083E+01 ± 2.8908E+01	7.2062E-01 ± 8.8196E-01
WHALE-QOBL	3.1741E+02 ± 1.6750E+02	2.1490E+00 ± 8.3302E-01	8.0969E-02 ± 5.5906E-02	2.4521E+03 ± 8.9207E+02	3.2079E+01 ± 1.2710E+01
WHALE-COBL	8.5316E-01 ± 8.3256E-01	1.4084E-02 ± 8.6888E-03	4.2133E-05 ± 4.5856E-05	4.2609E+01 ± 2.3017E+01	3.9132E-01 ± 2.3935E-01
WHALE-DOBL	6.6704E+02 ± 1.9899E+02	3.5886E+00 ± 1.2018E+00	1.0199E+00 ± 8.4767E-01	4.1164E+03 ± 1.2549E+03	7.9287E+01 ± 5.1040E+01
WHALE-EOBL	3.0024E+01 ± 2.2869E+01	9.2357E-01 ± 5.6998E-01	1.5035E-03 ± 1.0460E-03	4.7053E+02 ± 2.1685E+02	5.8585E+00 ± 4.0674E+00
WHALE-GOBL	7.2898E+02 ± 1.0547E+02	5.8032E-01 ± 1.0355E+00	8.8091E-01 ± 3.5827E-01	3.7622E+03 ± 5.1059E+02	2.3286E+02 ± 4.2220E+01
WHALE	2.7274E+00 ± 5.0515E+00	2.0419E-01 ± 2.1732E-01	5.7633E-05 ± 7.0047E-05	9.2879E+01 ± 8.1761E+01	6.2663E-01 ± 5.9618E-01

**Table 10:** Friedman ranking of the nine WHALE variants over the 20 benchmark functions at D = 500, with fractional best-function credits (ties split equally).

Algorithm	Average Rank	Rank Sum	Overall Rank	Best-Function Credit
WHALE-EDOFAS	1.65	33	1	15.2
WHALE-OPADAMP	2.35	47	2	2.2
WHALE-COBL	3.35	67	3	1.2
WHALE-OBL	4.35	87	4.5	0.2
WHALE	4.35	87	4.5	0.2
WHALE-EOBL	5.60	112	6	0.0
WHALE-QOBL	7.05	141	7	0.0
WHALE-DOBL	8.00	160	8	1.0
WHALE-GOBL	8.30	166	9	0.0

**Table 11:** Friedman ranking of the nine WHALE variants over the 20 benchmark problems at D = 1000, with fractional best-function credits.

Algorithm	Average Rank	Rank Sum	Overall Rank	Best-Function Credit
WHALE-EDOFAS	1.575	31.5	1	15.25
WHALE-OPADAMP	2.375	47.5	2	2.25
WHALE-COBL	3.450	69.0	3	1.00
WHALE-OBL	4.125	82.5	4	0.25
WHALE	4.725	94.5	5	0.25
WHALE-EOBL	5.750	115.0	6	0.00
WHALE-QOBL	6.850	137.0	7	0.00
WHALE-DOBL	8.050	161.0	8	1.00
WHALE-GOBL	8.100	162.0	9	0.00

**Table 12:** Statistical results of SAWHALE and the five surrogate-assisted comparators on the 30-dimensional CEC 2014 test problems (mean ± standard deviation of the final objective error over 30 independent runs; budget 50·D exact evaluations).

Function	SAWHALE	NRO	TLSAPSO	GPEQI	MSASFS	ESPSO
F1	3.0200E+07 ± 2.1255E+07	3.9811E+07 ± 2.2760E+07	6.6351E+07 ± 3.6692E+07	5.5188E+00 ± 3.6860E+00	5.9242E+07 ± 1.4882E+08	1.5464E+09 ± 4.6337E+08
F2	1.1068E+03 ± 1.5946E+03	1.0372E+09 ± 3.1544E+09	7.2110E+08 ± 2.7554E+08	1.9968E+02 ± 1.5521E+02	3.8970E+07 ± 5.1196E+07	9.8939E+10 ± 1.1591E+10
F3	7.8354E+04 ± 1.8313E+04	1.6884E+05 ± 4.5856E+04	8.6957E+04 ± 2.4353E+04	9.2029E-03 ± 9.2380E-03	2.4547E+04 ± 4.9237E+04	2.0024E+05 ± 2.3072E+04
F4	1.9738E+02 ± 4.0276E+01	1.8617E+02 ± 9.4315E+01	4.5102E+02 ± 1.6854E+02	3.3556E+03 ± 9.9971E+02	4.7029E+03 ± 1.9202E+03	1.4409E+04 ± 4.6058E+03
F5	2.0559E+01 ± 5.5234E-01	2.1209E+01 ± 6.0392E-02	2.1181E+01 ± 5.4312E-02	2.1178E+01 ± 6.2128E-02	2.1150E+01 ± 8.5264E-02	2.1168E+01 ± 6.4853E-02
F6	2.5439E+01 ± 4.7148E+00	2.5919E+01 ± 3.7464E+00	2.7433E+01 ± 2.6962E+00	3.7253E+01 ± 2.0528E+00	3.9728E+01 ± 3.4622E+00	4.5325E+01 ± 1.6669E+00
F7	1.6947E-02 ± 1.1659E-02	7.1023E+00 ± 9.1375E+00	4.2820E+01 ± 2.5171E+01	3.3971E+02 ± 4.2294E+01	2.9801E+02 ± 9.3431E+01	6.7692E+02 ± 9.2255E+01
F8	6.3644E+01 ± 1.8978E+01	3.2785E+02 ± 9.8983E+01	2.1517E+02 ± 2.5813E+01	3.2816E+02 ± 2.2801E+01	2.9938E+02 ± 2.6571E+01	4.2493E+02 ± 2.9539E+01
F9	8.0411E+01 ± 3.0112E+01	2.4512E+02 ± 9.3661E+01	2.8105E+02 ± 3.5736E+01	3.2394E+02 ± 1.9791E+01	3.7080E+02 ± 4.7447E+01	5.2039E+02 ± 4.3925E+01
F10	3.5519E+03 ± 1.0928E+03	8.8549E+03 ± 5.9166E+02	5.9027E+03 ± 6.5149E+02	7.6475E+03 ± 4.9717E+02	6.3254E+03 ± 5.5429E+02	7.8036E+03 ± 4.3538E+02
F11	4.1708E+03 ± 7.9001E+02	7.8810E+03 ± 1.7717E+03	7.5380E+03 ± 6.8935E+02	8.3420E+03 ± 4.2394E+02	8.3297E+03 ± 5.1620E+02	8.5296E+03 ± 3.0672E+02
F12	1.9166E+00 ± 1.8098E+00	4.6641E+00 ± 5.4531E-01	4.2078E+00 ± 5.2961E-01	4.1867E+00 ± 5.3506E-01	4.2019E+00 ± 6.7613E-01	4.2148E+00 ± 6.9261E-01
F13	5.4993E-01 ± 1.0951E-01	8.5004E-01 ± 1.4935E-01	1.0793E+00 ± 4.3639E-01	4.8255E+00 ± 3.7783E-01	4.8691E+00 ± 5.7193E-01	6.8744E+00 ± 6.0955E-01
F14	3.8749E-01 ± 5.5453E-02	1.8438E+00 ± 3.6747E+00	1.5635E+01 ± 8.9875E+00	1.2088E+02 ± 2.2465E+01	6.8109E+01 ± 1.7892E+01	1.8697E+02 ± 3.1896E+01
F15	2.6170E+04 ± 1.8110E+04	1.1280E+03 ± 4.2688E+03	5.0911E+03 ± 9.7895E+03	3.4733E+04 ± 1.9842E+04	2.5381E+04 ± 3.4401E+04	1.4885E+07 ± 7.0153E+06
F16	1.3152E+01 ± 5.2123E-01	1.3543E+01 ± 3.2289E-01	1.3464E+01 ± 2.5534E-01	1.3639E+01 ± 1.7496E-01	1.3830E+01 ± 2.0365E-01	1.3913E+01 ± 2.0467E-01
F17	2.8997E+07 ± 1.9117E+07	4.3509E+06 ± 3.0527E+06	8.8324E+06 ± 1.0949E+07	6.9695E+07 ± 5.1065E+07	4.4668E+07 ± 2.6735E+07	7.2729E+07 ± 3.7305E+07
F18	5.4120E+06 ± 1.1058E+07	1.8946E+04 ± 5.8586E+04	2.1789E+06 ± 9.3271E+06	1.5840E+08 ± 6.4861E+07	4.1226E+08 ± 2.2326E+08	9.7740E+08 ± 7.5096E+08
F19	1.1697E+02 ± 2.1567E+01	4.5612E+01 ± 3.9693E+01	7.2285E+01 ± 4.5866E+01	1.9172E+02 ± 5.0280E+01	1.8727E+02 ± 5.9336E+01	4.0139E+02 ± 1.1156E+02
F20	4.3264E+05 ± 3.9308E+05	1.3002E+06 ± 1.5704E+06	1.0793E+05 ± 8.2453E+04	1.6152E+05 ± 8.2399E+04	3.9688E+05 ± 2.6751E+05	2.0150E+06 ± 1.4569E+06
F21	1.7019E+07 ± 1.1707E+07	1.4618E+06 ± 2.2897E+06	1.1273E+06 ± 9.1428E+05	1.1185E+07 ± 6.3543E+06	1.7197E+07 ± 1.0324E+07	3.6611E+07 ± 1.7903E+07
F22	1.3536E+03 ± 2.3989E+02	8.6230E+02 ± 3.2027E+02	8.7849E+02 ± 2.2230E+02	1.3541E+03 ± 1.3793E+02	1.4101E+03 ± 2.1705E+02	1.3800E+03 ± 1.8174E+02
F23	3.9377E+02 ± 1.6851E+01	3.8092E+02 ± 3.4553E+01	4.0974E+02 ± 2.1040E+01	6.2241E+02 ± 8.0887E+01	5.7740E+02 ± 8.6818E+01	1.1174E+03 ± 2.0400E+02
F24	2.0191E+02 ± 5.2846E-01	2.0187E+02 ± 8.4298E-01	2.1316E+02 ± 6.9583E+00	2.6207E+02 ± 1.3109E+01	2.8174E+02 ± 6.2727E+01	3.1290E+02 ± 1.5698E+01
F25	2.3303E+02 ± 1.0842E+01	2.2600E+02 ± 1.5768E+01	2.4173E+02 ± 1.6086E+01	2.9608E+02 ± 1.0027E+01	2.5839E+02 ± 2.4282E+01	3.3677E+02 ± 3.7123E+01
F26	1.0411E+02 ± 4.0531E-01	1.0066E+02 ± 1.5175E-01	1.0080E+02 ± 3.0991E-01	1.0301E+02 ± 2.7609E-01	1.0419E+02 ± 6.3961E-01	1.0556E+02 ± 4.4835E-01
F27	7.5064E+02 ± 6.0030E+01	8.1627E+02 ± 2.8947E+02	7.9741E+02 ± 2.6297E+02	6.0216E+02 ± 6.6183E+01	1.2171E+03 ± 1.3596E+02	1.3274E+03 ± 7.8610E+01
F28	5.3289E+02 ± 6.9273E+01	5.6155E+02 ± 1.1764E+02	5.4007E+02 ± 7.8164E+01	1.3933E+03 ± 1.4782E+02	8.0640E+02 ± 1.7224E+02	1.2959E+03 ± 2.7735E+02
F29	9.2797E+06 ± 3.7557E+06	3.4540E+05 ± 1.7501E+06	4.9146E+06 ± 7.3977E+06	1.9914E+08 ± 7.3442E+07	4.2819E+06 ± 2.5047E+06	6.1467E+06 ± 2.0619E+06
F30	3.0324E+06 ± 1.2268E+06	4.8998E+05 ± 9.6304E+05	4.0969E+05 ± 4.2805E+05	1.4836E+07 ± 4.0049E+06	7.6008E+05 ± 9.1650E+05	9.3057E+05 ± 1.3152E+06

**Table 13:** Statistical results of SAWHALE and the five surrogate-assisted comparators on the 50-dimensional CEC 2014 test problems (mean ± standard deviation of the final objective error over 30 independent runs; budget 50·D exact evaluations).

Function	SAWHALE	NRO	TLSAPSO	GPEQI	MSASFS	ESPSO
F1	2.4112E+07 ± 7.7816E+06	1.9099E+08 ± 1.3783E+08	1.1036E+08 ± 5.7153E+07	2.2767E+01 ± 1.6894E+01	1.5542E+07 ± 3.9227E+07	3.3163E+09 ± 7.0013E+08
F2	5.7151E+03 ± 6.0738E+03	1.4120E+10 ± 1.7178E+10	4.6277E+09 ± 3.6582E+09	7.2955E+02 ± 4.0259E+02	4.8078E+07 ± 1.4333E+07	1.7812E+11 ± 1.6112E+10
F3	9.7077E+04 ± 1.6030E+04	4.9436E+05 ± 3.6806E+05	1.4837E+05 ± 2.5067E+04	4.6386E-02 ± 5.2173E-02	1.7253E+04 ± 6.0556E+04	3.3618E+05 ± 4.6444E+04
F4	2.5341E+02 ± 9.8664E+01	5.1459E+02 ± 4.2404E+02	2.1418E+03 ± 1.1902E+03	1.4512E+04 ± 2.8582E+03	8.3061E+03 ± 2.3054E+03	2.4186E+04 ± 7.6173E+03
F5	2.0394E+01 ± 5.7615E-01	2.1358E+01 ± 4.0079E-02	2.1300E+01 ± 4.7957E-02	2.1321E+01 ± 3.2434E-02	2.1291E+01 ± 4.6295E-02	2.1309E+01 ± 3.8381E-02
F6	4.4890E+01 ± 6.3339E+00	5.6699E+01 ± 1.3797E+01	5.0168E+01 ± 4.1057E+00	6.5555E+01 ± 2.7344E+00	7.3916E+01 ± 4.3998E+00	7.9330E+01 ± 1.9085E+00
F7	3.8097E-02 ± 1.9767E-02	6.8127E+01 ± 5.6129E+01	1.4062E+02 ± 6.3227E+01	7.2598E+02 ± 7.3637E+01	4.7927E+02 ± 1.0903E+02	1.2631E+03 ± 1.5010E+02
F8	1.1498E+02 ± 2.7103E+01	6.2260E+02 ± 7.0640E+01	3.7121E+02 ± 5.1168E+01	6.0143E+02 ± 3.8795E+01	5.4178E+02 ± 4.9205E+01	6.8850E+02 ± 3.6498E+01
F9	1.6729E+02 ± 3.5586E+01	7.6724E+02 ± 1.3623E+02	4.9913E+02 ± 6.2782E+01	6.1569E+02 ± 2.9569E+01	6.7505E+02 ± 5.8577E+01	8.8982E+02 ± 5.1372E+01
F10	6.0952E+03 ± 1.0065E+03	1.6129E+04 ± 6.8649E+02	9.9833E+03 ± 1.2648E+03	1.4103E+04 ± 7.0793E+02	1.1343E+04 ± 8.5120E+02	1.4561E+04 ± 4.6188E+02
F11	7.2085E+03 ± 1.6212E+03	1.5827E+04 ± 5.6513E+02	1.3024E+04 ± 1.4902E+03	1.5101E+04 ± 4.2255E+02	1.4958E+04 ± 5.5397E+02	1.5146E+04 ± 5.5863E+02
F12	1.5824E+00 ± 1.6968E+00	5.7016E+00 ± 6.3430E-01	4.8288E+00 ± 7.2850E-01	5.1623E+00 ± 5.0449E-01	4.8178E+00 ± 6.3167E-01	5.1102E+00 ± 4.7714E-01
F13	5.0088E-01 ± 1.1434E-01	1.0919E+00 ± 3.7161E-01	1.5272E+00 ± 8.2748E-01	5.8375E+00 ± 3.0665E-01	4.8762E+00 ± 5.1128E-01	7.3223E+00 ± 5.5355E-01
F14	3.9802E-01 ± 5.5420E-02	1.1563E+01 ± 1.7173E+01	4.2082E+01 ± 1.7667E+01	1.7365E+02 ± 1.8032E+01	1.0572E+02 ± 1.6369E+01	3.5452E+02 ± 3.6933E+01
F15	4.6538E+05 ± 2.4844E+05	2.3569E+04 ± 6.5556E+04	3.1886E+04 ± 5.1399E+04	4.0263E+05 ± 1.5587E+05	1.0613E+06 ± 1.6098E+06	2.9364E+07 ± 1.2719E+07
F16	2.2417E+01 ± 7.2041E-01	2.3594E+01 ± 3.3280E-01	2.3202E+01 ± 2.2312E-01	2.3179E+01 ± 2.1874E-01	2.3613E+01 ± 2.3268E-01	2.3676E+01 ± 1.7417E-01
F17	4.2543E+07 ± 2.3266E+07	5.6363E+07 ± 7.8365E+07	2.9105E+07 ± 2.2569E+07	2.3024E+08 ± 7.4075E+07	1.7051E+08 ± 7.6243E+07	2.9396E+08 ± 9.5375E+07
F18	4.5346E+06 ± 1.1503E+07	5.0617E+04 ± 2.5362E+05	1.2100E+08 ± 2.0052E+08	3.1317E+09 ± 1.1563E+09	7.5677E+08 ± 5.7138E+08	1.8397E+09 ± 7.4766E+08
F19	2.0167E+02 ± 5.4866E+01	7.6582E+01 ± 3.9502E+01	1.5985E+02 ± 1.0677E+02	4.5147E+02 ± 9.0116E+01	3.7333E+02 ± 1.2119E+02	5.1799E+02 ± 1.6331E+02
F20	5.3136E+05 ± 3.2295E+05	8.6763E+06 ± 1.0898E+07	1.5549E+05 ± 1.0278E+05	1.1796E+05 ± 4.8558E+04	2.3356E+06 ± 1.3466E+06	3.1374E+06 ± 2.0690E+06
F21	2.8590E+07 ± 1.1106E+07	7.7059E+07 ± 6.7740E+07	1.2593E+07 ± 1.0563E+07	2.2533E+07 ± 8.8335E+06	4.7809E+07 ± 2.8188E+07	1.3205E+08 ± 4.6134E+07
F22	3.1818E+03 ± 3.5860E+02	2.5975E+03 ± 4.3603E+02	1.6175E+03 ± 3.5900E+02	3.3580E+03 ± 6.8690E+02	2.7267E+03 ± 3.0912E+02	2.8252E+03 ± 2.2649E+02
F23	3.7375E+02 ± 2.1003E+01	3.9348E+02 ± 5.6514E+01	4.1582E+02 ± 4.5648E+01	4.0084E+02 ± 1.1016E+01	4.0895E+02 ± 1.7859E+01	1.4925E+03 ± 2.4533E+02
F24	2.0789E+02 ± 1.1333E+00	2.1820E+02 ± 1.3979E+01	2.7335E+02 ± 8.1912E+01	3.5268E+02 ± 3.2288E+01	3.4276E+02 ± 3.3197E+01	4.4887E+02 ± 3.6875E+01
F25	2.5988E+02 ± 2.2263E+01	2.3311E+02 ± 1.5871E+01	2.5048E+02 ± 1.6881E+01	4.4219E+02 ± 3.9846E+01	3.4071E+02 ± 4.8001E+01	4.5088E+02 ± 5.1529E+01
F26	5.1973E+02 ± 5.7004E+02	5.1561E+02 ± 4.1623E+02	8.1160E+02 ± 6.3152E+02	4.0001E+02 ± 4.4289E-03	1.7043E+02 ± 1.3029E+02	2.1248E+02 ± 1.6517E+02
F27	4.6666E+02 ± 1.5325E+01	5.2248E+02 ± 5.6082E+01	5.7956E+02 ± 7.7318E+01	4.0001E+02 ± 4.2371E-03	4.5455E+02 ± 1.4785E+02	2.5354E+03 ± 3.7354E+02
F28	1.2673E+03 ± 5.8146E+02	1.1538E+03 ± 5.0451E+02	1.1007E+03 ± 1.6129E+02	4.0105E+02 ± 3.1760E-01	2.1488E+03 ± 7.8147E+02	4.5082E+03 ± 7.0091E+02
F29	4.8916E+07 ± 2.4150E+07	5.6744E+05 ± 2.0415E+06	1.8108E+07 ± 2.6661E+07	3.6351E+08 ± 1.6939E+08	2.0983E+07 ± 1.9028E+07	2.2040E+07 ± 9.0769E+06
F30	5.6964E+06 ± 2.9322E+06	5.3544E+06 ± 7.5994E+06	6.4776E+05 ± 5.2231E+05	1.2085E+07 ± 6.7489E+06	3.3296E+06 ± 2.4829E+06	7.5155E+06 ± 6.9951E+06

**Table 14:** Two-sided Wilcoxon signed-rank test results (α = 0.05) of SAWHALE against each surrogate-assisted comparator on the CEC 2014 problems, by function family. Each cell reports wins/ties/losses from SAWHALE’s perspective.

D	Family	NRO	TLSAPSO	GPEQI	MSASFS	ESPSO
	Unimodal (F1–F3)	2/1/0	2/1/0	0/0/3	1/1/1	3/0/0
30	Multimodal (F4–F16)	11/1/1	12/0/1	11/1/1	12/1/0	13/0/0
	Hybrid (F17–F22)	1/0/5	0/0/6	3/1/2	3/3/0	5/1/0
	Composition (F23–F30)	0/3/5	3/2/3	6/0/2	5/1/2	6/0/2
	Unimodal (F1–F3)	3/0/0	3/0/0	0/0/3	1/0/2	3/0/0
50	Multimodal (F4–F16)	12/0/1	12/0/1	12/1/0	13/0/0	13/0/0
	Hybrid (F17–F22)	3/1/2	1/0/5	2/3/1	5/0/1	5/0/1
	Composition (F23–F30)	3/3/2	4/2/2	5/0/3	4/0/4	5/1/2

**Table 15:** Statistical results of the full SAWHALE and its six ablated variants on the 30-dimensional CEC 2014 test problems (mean ± standard deviation of the final objective error over 30 independent runs; budget 50·D exact evaluations).

Function	SAWHALE	RNDSWTCH	SGLOBAL	SLOCAL	STDOBL	SREMOVED	OBLREM
F1	3.0200E+07 ± 2.1255E+07	2.9610E+05 ± 1.4139E+06	8.9461E+05 ± 1.3762E+06	2.6547E+03 ± 1.4207E+04	1.8603E+07 ± 3.3929E+07	1.8676E+03 ± 8.5778E+03	3.1170E+07 ± 5.2758E+07
F2	1.1068E+03 ± 1.5946E+03	5.2717E+04 ± 2.5066E+05	2.1026E+07 ± 2.1747E+07	4.2442E+04 ± 2.1785E+05	1.2790E+08 ± 2.1853E+08	4.4444E-01 ± 2.4292E+00	1.3935E+06 ± 2.1213E+06
F3	7.8354E+04 ± 1.8313E+04	4.8895E+04 ± 6.7584E+04	7.5814E+04 ± 6.1340E+04	3.3990E+04 ± 6.8756E+04	1.3778E+05 ± 9.6810E+04	2.9754E+04 ± 5.3929E+04	8.8368E+04 ± 9.2167E+04
F4	1.9738E+02 ± 4.0276E+01	7.6971E+03 ± 1.8241E+03	1.2670E+04 ± 3.2990E+03	7.2610E+03 ± 2.1137E+03	7.8137E+03 ± 1.8454E+03	1.4964E+04 ± 3.4736E+03	9.4034E+03 ± 2.5304E+03
F5	2.0559E+01 ± 5.5234E-01	2.1182E+01 ± 5.4305E-02	2.1195E+01 ± 8.6329E-02	2.1145E+01 ± 9.5495E-02	2.1177E+01 ± 7.0871E-02	2.1184E+01 ± 6.8639E-02	2.1095E+01 ± 7.9474E-02
F6	2.5439E+01 ± 4.7148E+00	4.1169E+01 ± 2.7214E+00	4.2964E+01 ± 2.0437E+00	4.1448E+01 ± 3.0748E+00	4.0949E+01 ± 3.0384E+00	4.1454E+01 ± 2.6778E+00	4.0871E+01 ± 2.0695E+00
F7	1.6947E-02 ± 1.1659E-02	3.1179E+02 ± 7.2991E+01	4.8216E+02 ± 9.3630E+01	2.9479E+02 ± 7.6371E+01	3.3565E+02 ± 6.5395E+01	5.5458E+02 ± 1.1158E+02	3.7683E+02 ± 8.5976E+01
F8	6.3644E+01 ± 1.8978E+01	3.0233E+02 ± 3.3018E+01	3.5093E+02 ± 2.7059E+01	2.9085E+02 ± 2.5670E+01	3.2089E+02 ± 3.7596E+01	3.5185E+02 ± 3.0180E+01	3.2234E+02 ± 4.3183E+01
F9	8.0411E+01 ± 3.0112E+01	3.3199E+02 ± 3.5920E+01	3.8671E+02 ± 3.2653E+01	3.2389E+02 ± 3.3719E+01	3.6236E+02 ± 4.1000E+01	3.7884E+02 ± 2.2376E+01	3.6741E+02 ± 4.8504E+01
F10	3.5519E+03 ± 1.0928E+03	7.1919E+03 ± 7.1061E+02	7.6962E+03 ± 4.2072E+02	6.9953E+03 ± 6.9037E+02	7.2180E+03 ± 5.8131E+02	7.6016E+03 ± 4.8456E+02	7.2960E+03 ± 5.3574E+02
F11	4.1708E+03 ± 7.9001E+02	7.8658E+03 ± 6.8098E+02	8.0130E+03 ± 5.6481E+02	7.4906E+03 ± 6.5001E+02	7.9379E+03 ± 5.6080E+02	8.0628E+03 ± 6.1188E+02	7.8966E+03 ± 5.7109E+02
F12	1.9166E+00 ± 1.8098E+00	4.1086E+00 ± 6.7254E-01	4.1401E+00 ± 6.1322E-01	3.4033E+00 ± 7.6000E-01	4.2024E+00 ± 5.6182E-01	4.1059E+00 ± 6.6364E-01	3.3835E+00 ± 6.0036E-01
F13	5.4993E-01 ± 1.0951E-01	5.9618E+00 ± 6.1163E-01	7.4725E+00 ± 8.5007E-01	5.8327E+00 ± 5.6612E-01	5.7762E+00 ± 6.7848E-01	7.9635E+00 ± 9.2071E-01	6.7360E+00 ± 8.8478E-01
F14	3.8749E-01 ± 5.5453E-02	1.3572E+02 ± 2.7167E+01	1.9960E+02 ± 3.3950E+01	1.4390E+02 ± 2.1616E+01	1.4618E+02 ± 2.7494E+01	2.2536E+02 ± 2.9194E+01	1.8799E+02 ± 3.2514E+01
F15	2.6170E+04 ± 1.8110E+04	4.4118E+04 ± 1.9229E+04	1.2873E+05 ± 4.9814E+04	3.5940E+04 ± 1.7946E+04	4.3527E+04 ± 2.5546E+04	2.0000E+05 ± 5.3794E+04	9.4245E+04 ± 5.6651E+04
F16	1.3152E+01 ± 5.2123E-01	1.3353E+01 ± 4.2532E-01	1.3462E+01 ± 2.3852E-01	1.3271E+01 ± 3.4903E-01	1.3544E+01 ± 3.0533E-01	1.3524E+01 ± 3.5310E-01	1.3468E+01 ± 4.2010E-01
F17	2.8997E+07 ± 1.9117E+07	1.1679E+08 ± 7.6245E+07	1.7318E+08 ± 1.4183E+08	1.4255E+08 ± 1.1077E+08	1.0549E+08 ± 8.4174E+07	2.7514E+08 ± 1.6046E+08	1.6907E+08 ± 1.8278E+08
F18	5.4120E+06 ± 1.1058E+07	4.8391E+08 ± 4.5510E+08	3.1588E+09 ± 1.6159E+09	5.0810E+08 ± 3.6321E+08	8.6412E+08 ± 8.4859E+08	3.2506E+09 ± 1.7813E+09	9.9133E+08 ± 7.8518E+08
F19	1.1697E+02 ± 2.1567E+01	3.0255E+02 ± 7.9578E+01	4.1248E+02 ± 1.2773E+02	2.8255E+02 ± 7.7273E+01	3.4459E+02 ± 1.1331E+02	4.5714E+02 ± 1.5008E+02	3.4297E+02 ± 1.0361E+02
F20	4.3264E+05 ± 3.9308E+05	8.7767E+05 ± 9.7483E+05	1.0913E+06 ± 1.9075E+06	8.0988E+05 ± 9.9673E+05	7.0185E+05 ± 7.3932E+05	1.6073E+06 ± 2.0573E+06	1.2430E+06 ± 2.0408E+06
F21	1.7019E+07 ± 1.1707E+07	4.8388E+07 ± 3.2291E+07	5.4754E+07 ± 4.6025E+07	4.8508E+07 ± 4.6973E+07	4.3666E+07 ± 3.0945E+07	5.9754E+07 ± 4.4304E+07	5.1126E+07 ± 4.1919E+07
F22	1.3536E+03 ± 2.3989E+02	1.5447E+03 ± 3.9679E+02	1.8762E+03 ± 6.8047E+02	1.4861E+03 ± 4.3187E+02	1.4568E+03 ± 4.5176E+02	6.3318E+03 ± 2.3637E+04	1.5739E+03 ± 5.1782E+02
F23	3.9377E+02 ± 1.6851E+01	7.0403E+02 ± 8.8081E+01	9.5364E+02 ± 1.7333E+02	7.2222E+02 ± 1.5291E+02	7.3788E+02 ± 1.5791E+02	1.1865E+03 ± 4.5941E+02	7.9090E+02 ± 1.8140E+02
F24	2.0191E+02 ± 5.2846E-01	2.8690E+02 ± 2.8970E+01	3.5186E+02 ± 3.5761E+01	2.7878E+02 ± 2.5001E+01	2.8580E+02 ± 2.4756E+01	3.6207E+02 ± 4.5479E+01	3.1123E+02 ± 4.1843E+01
F25	2.3303E+02 ± 1.0842E+01	3.0794E+02 ± 3.2439E+01	3.4569E+02 ± 5.6233E+01	2.9433E+02 ± 2.9602E+01	3.2588E+02 ± 6.2802E+01	3.6505E+02 ± 6.5814E+01	3.2836E+02 ± 6.6885E+01
F26	1.0411E+02 ± 4.0531E-01	1.0434E+02 ± 6.6881E-01	1.0591E+02 ± 1.4215E+00	1.0450E+02 ± 8.6934E-01	1.0477E+02 ± 1.0806E+00	1.0681E+02 ± 1.8278E+00	1.0504E+02 ± 1.2340E+00
F27	7.5064E+02 ± 6.0030E+01	1.0090E+03 ± 3.0314E+02	1.1366E+03 ± 2.7111E+02	1.0168E+03 ± 2.6253E+02	1.1855E+03 ± 2.9058E+02	1.2843E+03 ± 2.4474E+02	1.2614E+03 ± 2.7775E+02
F28	5.3289E+02 ± 6.9273E+01	1.6102E+03 ± 6.4323E+02	1.7949E+03 ± 3.9215E+02	1.5546E+03 ± 3.8021E+02	1.6652E+03 ± 4.9830E+02	2.1149E+03 ± 5.0207E+02	1.6413E+03 ± 5.0122E+02
F29	9.2797E+06 ± 3.7557E+06	6.0486E+07 ± 4.9809E+07	5.0223E+07 ± 4.9606E+07	6.0887E+07 ± 4.8178E+07	7.0294E+07 ± 6.1822E+07	8.9817E+07 ± 7.4577E+07	4.1788E+07 ± 2.1866E+07
F30	3.0324E+06 ± 1.2268E+06	2.8574E+07 ± 2.6306E+07	3.5187E+07 ± 2.5889E+07	2.0406E+07 ± 1.1537E+07	2.3039E+07 ± 1.9038E+07	3.5705E+07 ± 1.9668E+07	1.7640E+07 ± 1.3795E+07

**Table 16:** Statistical results of the full SAWHALE and its six ablated variants on the 50-dimensional CEC 2014 test problems (mean ± standard deviation of the final objective error over 30 independent runs; budget 50·D exact evaluations).

Function	SAWHALE	RNDSWTCH	SGLOBAL	SLOCAL	STDOBL	SREMOVED	OBLREM
F1	2.4112E+07 ± 7.7816E+06	2.1099E-02 ± 1.0313E-01	9.9566E+03 ± 1.8762E+04	2.5704E-03 ± 1.0996E-02	5.2662E+05 ± 1.2243E+06	1.2498E-09 ± 4.3418E-09	1.4347E+05 ± 5.0278E+05
F2	5.7151E+03 ± 6.0738E+03	4.9792E-02 ± 1.6098E-01	1.2941E+06 ± 2.3583E+06	2.2743E-02 ± 7.5021E-02	6.7207E+06 ± 9.3950E+06	2.0496E-09 ± 1.1226E-08	1.7483E+03 ± 1.9942E+03
F3	9.7077E+04 ± 1.6030E+04	4.5268E+03 ± 2.2129E+04	8.4705E+03 ± 2.6048E+04	1.9230E+04 ± 5.4778E+04	4.3550E+04 ± 5.8544E+04	3.5475E-03 ± 1.2237E-02	7.2010E+04 ± 8.5279E+04
F4	2.5341E+02 ± 9.8664E+01	1.7519E+04 ± 3.6023E+03	3.7875E+04 ± 5.4860E+03	1.6115E+04 ± 3.3019E+03	1.8024E+04 ± 4.1849E+03	4.3395E+04 ± 7.9643E+03	3.3019E+04 ± 6.2420E+03
F5	2.0394E+01 ± 5.7615E-01	2.1319E+01 ± 3.6201E-02	2.1309E+01 ± 4.2989E-02	2.1263E+01 ± 5.5128E-02	2.1316E+01 ± 4.8908E-02	2.1336E+01 ± 3.5634E-02	2.1249E+01 ± 5.7262E-02
F6	4.4890E+01 ± 6.3339E+00	7.2609E+01 ± 3.4609E+00	7.5740E+01 ± 2.8232E+00	7.1203E+01 ± 4.4440E+00	7.3027E+01 ± 3.9478E+00	7.4674E+01 ± 2.5083E+00	7.6147E+01 ± 2.4576E+00
F7	3.8097E-02 ± 1.9767E-02	7.9106E+02 ± 1.0453E+02	1.2745E+03 ± 1.4988E+02	7.4004E+02 ± 8.3441E+01	8.3631E+02 ± 1.3655E+02	1.3857E+03 ± 1.6861E+02	1.1546E+03 ± 1.3301E+02
F8	1.1498E+02 ± 2.7103E+01	5.5046E+02 ± 3.2151E+01	6.2131E+02 ± 3.1404E+01	5.3465E+02 ± 2.9392E+01	5.6334E+02 ± 4.5055E+01	6.3441E+02 ± 3.3170E+01	6.2620E+02 ± 4.3436E+01
F9	1.6729E+02 ± 3.5586E+01	6.6971E+02 ± 4.9358E+01	7.4412E+02 ± 3.7111E+01	6.4444E+02 ± 3.8127E+01	6.7742E+02 ± 6.0029E+01	7.3835E+02 ± 4.6174E+01	7.4343E+02 ± 6.3687E+01
F10	6.0952E+03 ± 1.0065E+03	1.3076E+04 ± 1.0400E+03	1.4220E+04 ± 7.5870E+02	1.2477E+04 ± 7.9406E+02	1.3676E+04 ± 8.9942E+02	1.4167E+04 ± 6.8480E+02	1.3660E+04 ± 8.2441E+02
F11	7.2085E+03 ± 1.6212E+03	1.4394E+04 ± 5.8345E+02	1.4908E+04 ± 4.4989E+02	1.3930E+04 ± 8.6517E+02	1.4584E+04 ± 5.7429E+02	1.4808E+04 ± 6.0780E+02	1.4370E+04 ± 7.2479E+02
F12	1.5824E+00 ± 1.6968E+00	4.6087E+00 ± 6.9307E-01	5.0360E+00 ± 5.6638E-01	4.2755E+00 ± 7.5520E-01	4.5368E+00 ± 6.6793E-01	5.0232E+00 ± 6.7661E-01	3.9844E+00 ± 7.3603E-01
F13	5.0088E-01 ± 1.1434E-01	6.1624E+00 ± 4.8640E-01	8.0394E+00 ± 5.9589E-01	5.9424E+00 ± 3.6765E-01	6.1395E+00 ± 4.8417E-01	8.5492E+00 ± 4.9343E-01	7.6491E+00 ± 5.9159E-01
F14	3.9802E-01 ± 5.5420E-02	1.7144E+02 ± 1.9786E+01	2.8855E+02 ± 3.7519E+01	1.6127E+02 ± 2.5067E+01	1.6570E+02 ± 2.2941E+01	3.0983E+02 ± 2.9093E+01	2.5515E+02 ± 3.3027E+01
F15	4.6538E+05 ± 2.4844E+05	1.3941E+06 ± 7.0905E+05	4.4836E+06 ± 2.4638E+06	1.1432E+06 ± 4.0970E+05	1.2679E+06 ± 6.8520E+05	6.5627E+06 ± 3.4778E+06	4.4105E+06 ± 2.5108E+06
F16	2.2417E+01 ± 7.2041E-01	2.3143E+01 ± 3.3646E-01	2.3306E+01 ± 2.4872E-01	2.2985E+01 ± 3.9755E-01	2.3296E+01 ± 3.0862E-01	2.3190E+01 ± 3.2469E-01	2.3067E+01 ± 4.0500E-01
F17	4.2543E+07 ± 2.3266E+07	7.2197E+08 ± 2.6201E+08	1.0122E+09 ± 3.5761E+08	6.1252E+08 ± 2.3868E+08	7.3711E+08 ± 2.5883E+08	9.7314E+08 ± 3.3079E+08	7.6772E+08 ± 2.7709E+08
F18	4.5346E+06 ± 1.1503E+07	2.3705E+09 ± 8.2629E+08	1.0742E+10 ± 2.5728E+09	1.7283E+09 ± 8.7561E+08	2.4890E+09 ± 1.3187E+09	1.4293E+10 ± 3.9598E+09	6.7040E+09 ± 3.0160E+09
F19	2.0167E+02 ± 5.4866E+01	7.9565E+02 ± 2.6265E+02	1.6630E+03 ± 6.1110E+02	7.7403E+02 ± 2.0534E+02	8.5350E+02 ± 2.2133E+02	2.3417E+03 ± 9.1509E+02	1.2927E+03 ± 5.0092E+02
F20	5.3136E+05 ± 3.2295E+05	2.3041E+06 ± 2.7184E+06	5.7182E+06 ± 6.1753E+06	2.0145E+06 ± 2.5049E+06	4.7942E+06 ± 6.0664E+06	2.5138E+06 ± 2.5846E+06	5.4678E+06 ± 6.5063E+06
F21	2.8590E+07 ± 1.1106E+07	6.2147E+07 ± 4.3883E+07	1.2217E+08 ± 7.7617E+07	6.1743E+07 ± 3.7162E+07	6.8920E+07 ± 4.0267E+07	1.5233E+08 ± 9.7212E+07	7.7274E+07 ± 5.0200E+07
F22	3.1818E+03 ± 3.5860E+02	2.0873E+04 ± 4.2774E+04	4.1856E+04 ± 6.9653E+04	5.4629E+03 ± 4.5790E+03	1.3540E+04 ± 1.4041E+04	1.0022E+05 ± 1.2154E+05	2.1400E+04 ± 3.5957E+04
F23	3.7375E+02 ± 2.1003E+01	4.0000E+02 ± 9.2229E-04	4.0245E+02 ± 2.1687E+00	4.0000E+02 ± 9.2248E-04	4.1211E+02 ± 1.9161E+01	4.0000E+02 ± 1.1205E-08	4.0032E+02 ± 8.1785E-01
F24	2.0789E+02 ± 1.1333E+00	4.6481E+02 ± 1.5593E+02	6.9141E+02 ± 1.5382E+02	4.2946E+02 ± 4.8940E+01	4.7549E+02 ± 1.3829E+02	7.7439E+02 ± 1.3604E+02	6.1520E+02 ± 1.0900E+02
F25	2.5988E+02 ± 2.2263E+01	4.8857E+02 ± 1.1677E+02	5.7346E+02 ± 8.2186E+01	4.7690E+02 ± 6.5103E+01	4.7362E+02 ± 8.8656E+01	6.7194E+02 ± 9.5994E+01	5.9209E+02 ± 1.2375E+02
F26	5.1973E+02 ± 5.7004E+02	3.9096E+02 ± 8.3104E+01	4.0226E+02 ± 3.5357E+00	3.7438E+02 ± 9.2447E+01	4.6239E+02 ± 6.3460E+02	3.6248E+02 ± 9.7288E+01	6.7723E+02 ± 9.4013E+02
F27	4.6666E+02 ± 1.5325E+01	4.0000E+02 ± 1.2070E-04	4.0033E+02 ± 2.7177E-01	4.0000E+02 ± 2.8792E-04	4.0977E+02 ± 2.0108E+01	4.0000E+02 ± 8.0151E-09	4.0008E+02 ± 2.0017E-01
F28	1.2673E+03 ± 5.8146E+02	4.0003E+02 ± 1.2832E-01	6.1498E+02 ± 7.0791E+02	4.0000E+02 ± 1.9470E-02	2.2783E+03 ± 1.4628E+03	4.0000E+02 ± 1.8729E-06	3.4227E+03 ± 1.6273E+03
F29	4.8916E+07 ± 2.4150E+07	8.2331E+08 ± 3.6292E+08	4.4969E+08 ± 2.7966E+08	8.4627E+08 ± 4.8025E+08	8.5617E+08 ± 3.6168E+08	8.6161E+08 ± 4.5480E+08	6.5003E+08 ± 5.2604E+08
F30	5.6964E+06 ± 2.9322E+06	2.2671E+07 ± 1.4448E+07	3.3029E+07 ± 2.0730E+07	1.5598E+07 ± 9.2158E+06	2.0272E+07 ± 1.2186E+07	3.3856E+07 ± 2.5046E+07	2.7406E+07 ± 1.6407E+07

**Table 17:** Two-sided Wilcoxon signed-rank test results (α = 0.05) of the full SAWHALE against each ablated variant on the CEC 2014 problems, by function family. Each cell reports wins/ties/losses from the full framework’s perspective.

D	Family	RNDSWTCH	SGLOBAL	SLOCAL	STDOBL	SREMOVED	OBLREM
	Unimodal (F1–F3)	1/0/2	1/1/1	1/0/2	2/0/1	0/0/3	1/2/0
30	Multimodal (F4–F16)	13/0/0	12/1/0	13/0/0	13/0/0	13/0/0	13/0/0
	Hybrid (F17–F22)	5/1/0	5/1/0	4/2/0	4/2/0	6/0/0	6/0/0
	Composition (F23–F30)	7/1/0	8/0/0	8/0/0	8/0/0	8/0/0	8/0/0
	Unimodal (F1–F3)	0/0/3	1/0/2	0/0/3	1/0/2	0/0/3	0/0/3
50	Multimodal (F4–F16)	13/0/0	13/0/0	13/0/0	13/0/0	13/0/0	13/0/0
	Hybrid (F17–F22)	6/0/0	6/0/0	6/0/0	6/0/0	6/0/0	6/0/0
	Composition (F23–F30)	5/0/3	5/0/3	5/0/3	6/0/2	5/0/3	7/0/1

**Table 18:** Statistical results of SAWHALE and six recent metaheuristic competitors on the 30-dimensional CEC 2017 test problems (mean ± standard deviation of the final objective error over 30 independent runs; budget 50·D exact evaluations).

Function	SAWHALE	AOA	PARROT	SINHCOSH	DGSWOA	EWOA	MHWOA
F1	2.9569E+03 ± 2.9917E+03	5.0957E+10 ± 8.8059E+09	3.6419E+10 ± 6.2557E+09	4.5904E+10 ± 1.0888E+10	4.0260E+10 ± 8.4711E+09	2.7559E+10 ± 8.0301E+09	5.8891E+10 ± 5.6823E+09
F2	9.3062E+44 ± 3.0984E+45	7.9213E+50 ± 3.7463E+51	4.4567E+43 ± 1.6965E+44	2.6220E+44 ± 1.2713E+45	1.5133E+44 ± 6.6425E+44	6.1667E+43 ± 3.3080E+44	5.5676E+46 ± 1.2568E+47
F3	1.4923E+05 ± 3.4719E+04	1.2594E+05 ± 4.8909E+04	1.4864E+05 ± 6.3175E+04	1.2098E+05 ± 4.1951E+04	1.5976E+05 ± 4.0306E+04	8.7092E+04 ± 7.8761E+03	1.5758E+05 ± 6.3596E+04
F4	1.5120E+02 ± 3.6480E+01	1.3966E+04 ± 4.6347E+03	7.8508E+03 ± 2.4565E+03	1.3548E+04 ± 4.0550E+03	9.5204E+03 ± 1.8211E+03	6.7421E+03 ± 2.3964E+03	1.3791E+04 ± 2.9182E+03
F5	7.4917E+01 ± 2.2355E+01	4.1430E+02 ± 2.8520E+01	3.9466E+02 ± 4.1723E+01	4.3427E+02 ± 4.6506E+01	4.3011E+02 ± 4.2708E+01	3.7590E+02 ± 2.9808E+01	4.5300E+02 ± 2.6744E+01
F6	3.0435E+01 ± 1.1202E+01	1.0630E+02 ± 9.4218E+00	1.0722E+02 ± 1.3617E+01	1.2330E+02 ± 1.5897E+01	1.1153E+02 ± 1.2598E+01	1.0328E+02 ± 1.1000E+01	1.1768E+02 ± 9.1432E+00
F7	1.1713E+02 ± 3.2439E+01	7.6330E+02 ± 5.3860E+01	7.3020E+02 ± 6.3734E+01	8.5782E+02 ± 4.2895E+01	7.1594E+02 ± 9.5148E+01	6.2406E+02 ± 6.8536E+01	7.9616E+02 ± 4.6212E+01
F8	8.3277E+01 ± 2.5797E+01	3.5461E+02 ± 2.7114E+01	3.4445E+02 ± 3.2522E+01	3.6117E+02 ± 4.7118E+01	3.6254E+02 ± 3.9744E+01	3.0325E+02 ± 2.3565E+01	3.8258E+02 ± 2.5332E+01
F9	6.5899E+02 ± 4.6967E+02	1.0569E+04 ± 2.3574E+03	1.1371E+04 ± 2.0902E+03	1.6443E+04 ± 2.1025E+03	1.3518E+04 ± 1.8037E+03	1.0162E+04 ± 1.6869E+03	1.3871E+04 ± 1.5032E+03
F10	4.1019E+03 ± 1.0300E+03	7.4958E+03 ± 4.2609E+02	7.2377E+03 ± 5.9952E+02	7.1489E+03 ± 4.3748E+02	7.6069E+03 ± 5.0920E+02	6.6223E+03 ± 4.6611E+02	7.8885E+03 ± 3.2109E+02
F11	1.3515E+04 ± 4.8696E+03	9.3662E+03 ± 2.2266E+03	9.5257E+03 ± 3.2170E+03	1.0608E+04 ± 3.4992E+03	1.2554E+04 ± 5.6893E+03	4.5616E+03 ± 1.9390E+03	1.2593E+04 ± 4.7037E+03
F12	1.1620E+07 ± 1.7935E+07	1.3174E+10 ± 3.4085E+09	5.7370E+09 ± 1.8749E+09	8.0891E+09 ± 2.5466E+09	5.7278E+09 ± 1.6435E+09	5.3785E+09 ± 2.7797E+09	1.1687E+10 ± 2.1284E+09
F13	3.0449E+07 ± 7.4415E+07	1.6898E+10 ± 6.0641E+09	3.1238E+09 ± 2.0324E+09	4.0429E+09 ± 2.8201E+09	1.7680E+09 ± 1.3215E+09	1.6206E+09 ± 1.4884E+09	7.9863E+09 ± 3.3508E+09
F14	3.6556E+06 ± 3.6310E+06	2.0177E+07 ± 2.6700E+07	3.9297E+06 ± 4.5177E+06	4.6003E+06 ± 3.0931E+06	3.4393E+06 ± 2.2108E+06	1.7223E+06 ± 1.2048E+06	9.0072E+06 ± 5.9943E+06
F15	3.5598E+07 ± 5.0434E+07	1.5318E+09 ± 1.3510E+09	9.4109E+07 ± 8.2273E+07	2.3346E+08 ± 2.4727E+08	4.6393E+08 ± 3.0007E+08	2.3450E+07 ± 2.2854E+07	1.0984E+09 ± 5.0356E+08
F16	2.1797E+03 ± 3.2873E+02	3.6332E+03 ± 1.2709E+03	2.8659E+03 ± 5.6942E+02	2.6991E+03 ± 4.3609E+02	3.2030E+03 ± 7.2496E+02	2.7816E+03 ± 6.3212E+02	4.0960E+03 ± 4.8336E+02
F17	1.2969E+03 ± 1.9210E+02	4.5082E+03 ± 5.9605E+03	1.3850E+03 ± 3.1556E+02	1.3345E+03 ± 6.2438E+02	1.5793E+03 ± 3.3432E+02	1.1673E+03 ± 2.8099E+02	2.6875E+03 ± 6.6797E+02
F18	1.5319E+07 ± 1.2228E+07	6.3178E+07 ± 3.3171E+07	3.2393E+07 ± 3.1055E+07	2.8255E+07 ± 2.3853E+07	4.6440E+07 ± 3.8888E+07	1.3306E+07 ± 1.0901E+07	9.4978E+07 ± 6.2360E+07
F19	1.5971E+07 ± 1.8211E+07	2.7737E+09 ± 1.4071E+09	2.5186E+08 ± 3.1693E+08	3.3492E+08 ± 3.4971E+08	4.4400E+08 ± 2.9979E+08	5.9290E+07 ± 8.6287E+07	1.0974E+09 ± 6.2895E+08
F20	6.7433E+02 ± 2.7907E+02	1.0664E+03 ± 2.1148E+02	9.7802E+02 ± 1.9156E+02	9.5473E+02 ± 2.2644E+02	1.0845E+03 ± 1.7429E+02	8.8043E+02 ± 1.7678E+02	1.0644E+03 ± 2.1193E+02
F21	3.2678E+02 ± 5.3929E+01	6.4545E+02 ± 4.6696E+01	5.9328E+02 ± 5.3933E+01	5.9192E+02 ± 4.5878E+01	6.1504E+02 ± 5.6561E+01	5.7156E+02 ± 5.6824E+01	6.8257E+02 ± 3.3584E+01
F22	3.7402E+03 ± 2.3925E+03	7.5257E+03 ± 8.5970E+02	6.5379E+03 ± 1.8881E+03	6.6966E+03 ± 1.5087E+03	7.5921E+03 ± 1.0831E+03	5.8484E+03 ± 2.0294E+03	7.5971E+03 ± 1.1462E+03
F23	5.1488E+02 ± 5.7195E+01	1.1824E+03 ± 1.2859E+02	9.2756E+02 ± 1.1974E+02	1.0243E+03 ± 1.0492E+02	1.0346E+03 ± 9.4183E+01	1.0205E+03 ± 1.4230E+02	1.1609E+03 ± 9.5553E+01
F24	7.3224E+02 ± 6.0446E+01	1.6559E+03 ± 1.8797E+02	1.2226E+03 ± 1.3816E+02	1.5021E+03 ± 1.2206E+02	1.5569E+03 ± 1.6953E+02	1.4715E+03 ± 1.6431E+02	1.6645E+03 ± 1.6998E+02
F25	6.6754E+02 ± 8.2109E+01	4.4069E+03 ± 1.6880E+03	2.5890E+03 ± 5.6234E+02	3.3298E+03 ± 1.0686E+03	2.9206E+03 ± 6.0372E+02	2.4217E+03 ± 6.1566E+02	5.1047E+03 ± 1.1702E+03
F26	2.3741E+03 ± 3.0368E+02	7.3717E+03 ± 1.0862E+03	6.5589E+03 ± 1.0562E+03	6.6412E+03 ± 7.8578E+02	6.9236E+03 ± 1.0163E+03	5.9174E+03 ± 7.7995E+02	8.1845E+03 ± 8.3933E+02
F27	6.6697E+02 ± 3.5223E+01	1.3501E+03 ± 1.5928E+02	9.4212E+02 ± 1.0368E+02	1.0239E+03 ± 1.2943E+02	1.2346E+03 ± 1.4813E+02	1.2303E+03 ± 1.2784E+02	1.2681E+03 ± 1.2023E+02
F28	6.3641E+02 ± 4.7090E+01	3.4562E+03 ± 1.1220E+03	2.3936E+03 ± 6.9230E+02	2.4994E+03 ± 8.6492E+02	2.9473E+03 ± 7.5803E+02	2.6036E+03 ± 9.3513E+02	4.4614E+03 ± 8.0476E+02
F29	3.2361E+03 ± 5.0254E+02	3.3174E+03 ± 6.6589E+02	2.5605E+03 ± 5.7141E+02	2.5992E+03 ± 4.6662E+02	2.8736E+03 ± 3.4200E+02	2.9517E+03 ± 4.9636E+02	3.2779E+03 ± 3.5895E+02
F30	9.4492E+06 ± 1.8681E+07	4.0507E+09 ± 2.1045E+09	1.5345E+09 ± 8.5375E+08	1.3039E+09 ± 1.0677E+09	2.5181E+09 ± 1.1606E+09	1.9467E+09 ± 1.2886E+09	3.6906E+09 ± 1.4085E+09

**Table 19:** Statistical results of SAWHALE and six recent metaheuristic competitors on the 50-dimensional CEC 2017 test problems (mean ± standard deviation of the final objective error over 30 independent runs; budget 50·D exact evaluations).

Function	SAWHALE	AOA	PARROT	SINHCOSH	DGSWOA	EWOA	MHWOA
F1	4.6493E+03 ± 6.2943E+03	1.1288E+11 ± 9.8477E+09	7.3548E+10 ± 7.7596E+09	8.8805E+10 ± 1.2859E+10	7.9232E+10 ± 9.9243E+09	6.9733E+10 ± 1.1848E+10	1.0908E+11 ± 6.5523E+09
F2	6.1824E+83 ± 3.3529E+84	1.0017E+86 ± 5.4678E+86	3.1751E+74 ± 1.4386E+75	5.4691E+77 ± 2.9937E+78	1.2533E+79 ± 6.7584E+79	6.8843E+77 ± 3.7427E+78	6.7869E+81 ± 3.2313E+78
F3	2.6784E+05 ± 4.3755E+04	2.6560E+05 ± 4.5380E+04	3.3641E+05 ± 8.4330E+04	3.0308E+05 ± 8.9457E+04	3.3620E+05 ± 6.5014E+04	1.9283E+05 ± 3.2193E+04	3.3218E+05 ± 9.1430E+04
F4	3.4031E+02 ± 9.5639E+01	3.3306E+04 ± 6.3707E+03	1.7875E+04 ± 3.8602E+03	2.2597E+04 ± 5.4781E+03	2.4395E+04 ± 5.3045E+03	1.9658E+04 ± 4.8768E+03	3.6991E+04 ± 4.8809E+03
F5	1.6082E+02 ± 4.3489E+01	6.9519E+02 ± 3.6534E+01	6.4604E+02 ± 3.5027E+01	7.1311E+02 ± 5.3709E+01	7.0759E+02 ± 4.8264E+01	6.2401E+02 ± 3.7946E+01	7.0970E+02 ± 2.9845E+01
F6	4.2306E+01 ± 9.9929E+00	1.3316E+02 ± 7.9271E+00	1.3405E+02 ± 9.1075E+00	1.4154E+02 ± 1.7680E+01	1.3691E+02 ± 1.1057E+01	1.2568E+02 ± 1.1802E+01	1.3890E+02 ± 5.3747E+00
F7	2.8632E+02 ± 5.6333E+01	1.3541E+03 ± 5.5007E+01	1.2839E+03 ± 8.0811E+01	1.4113E+03 ± 3.8959E+01	1.3304E+03 ± 9.9640E+01	1.2501E+03 ± 8.6958E+01	1.3985E+03 ± 4.0800E+01
F8	1.6606E+02 ± 4.8427E+01	7.0913E+02 ± 3.8582E+01	6.7581E+02 ± 5.2638E+01	7.1296E+02 ± 6.5884E+01	6.8999E+02 ± 6.2249E+01	6.3380E+02 ± 4.0425E+01	7.1624E+02 ± 3.2434E+01
F9	2.8402E+03 ± 1.1612E+03	3.8402E+04 ± 3.3704E+03	3.7795E+04 ± 5.0871E+03	4.8712E+04 ± 7.5396E+03	4.4106E+04 ± 7.4048E+03	3.5466E+04 ± 4.2431E+03	4.0846E+04 ± 3.8479E+03
F10	6.7531E+03 ± 1.6110E+03	1.3526E+04 ± 6.3153E+02	1.3274E+04 ± 6.4083E+02	1.2580E+04 ± 8.9651E+02	1.3873E+04 ± 4.9879E+02	1.3113E+04 ± 9.6455E+02	1.3944E+04 ± 6.5267E+02
F11	3.6290E+04 ± 9.8326E+03	2.3268E+04 ± 3.4992E+03	1.9388E+04 ± 2.9915E+03	2.4677E+04 ± 5.0444E+03	2.1180E+04 ± 3.8091E+03	1.7739E+04 ± 3.1356E+03	2.6553E+04 ± 2.6107E+03
F12	2.2371E+07 ± 1.2473E+07	7.3511E+10 ± 1.4090E+10	2.8419E+10 ± 9.4486E+09	3.5535E+10 ± 1.1537E+10	3.6432E+10 ± 7.9413E+09	3.7800E+10 ± 1.0510E+10	6.4219E+10 ± 1.2656E+10
F13	6.8037E+06 ± 3.2952E+07	4.1838E+10 ± 1.2000E+10	8.5733E+09 ± 4.0625E+09	1.3553E+10 ± 8.6544E+09	8.9727E+09 ± 4.0201E+09	1.3328E+10 ± 8.9945E+09	3.3089E+10 ± 8.3228E+09
F14	1.2727E+07 ± 1.1807E+07	1.1018E+08 ± 8.2123E+07	2.2373E+07 ± 1.9949E+07	2.0586E+07 ± 2.1580E+07	2.3160E+07 ± 1.6181E+07	3.0065E+07 ± 2.7039E+07	5.3212E+07 ± 2.4674E+07
F15	1.2809E+08 ± 5.4925E+08	1.1826E+10 ± 4.5651E+09	1.0764E+09 ± 8.2517E+08	1.7479E+09 ± 1.6012E+09	2.1642E+09 ± 1.1957E+09	1.5035E+09 ± 1.3526E+09	6.2901E+09 ± 1.7420E+09
F16	1.6574E+03 ± 5.9743E+02	6.7224E+03 ± 1.4376E+03	5.1283E+03 ± 8.6035E+02	4.6120E+03 ± 1.0445E+03	5.4632E+03 ± 8.5805E+02	5.3947E+03 ± 8.9992E+02	6.9128E+03 ± 9.6570E+02
F17	4.3815E+03 ± 1.1147E+03	2.2207E+04 ± 1.3908E+04	3.2495E+03 ± 6.6568E+02	3.5617E+03 ± 2.5256E+03	3.5945E+03 ± 7.1933E+02	2.9955E+03 ± 7.0010E+02	1.4789E+04 ± 8.7409E+03
F18	4.3012E+07 ± 3.0593E+07	2.2380E+08 ± 1.2980E+08	6.2309E+07 ± 3.6654E+07	7.9951E+07 ± 5.8388E+07	1.1204E+08 ± 6.8221E+07	6.8306E+07 ± 3.5553E+07	1.9146E+08 ± 7.1647E+07
F19	6.9352E+07 ± 9.0432E+07	5.5520E+09 ± 2.0942E+09	4.9782E+08 ± 3.8372E+08	1.0379E+09 ± 9.8886E+08	4.9388E+08 ± 2.6445E+08	3.5752E+08 ± 4.2584E+08	3.0440E+09 ± 1.0638E+09
F20	1.4546E+03 ± 3.5957E+02	2.3778E+03 ± 1.8041E+02	2.1004E+03 ± 2.7416E+02	2.0431E+03 ± 3.5783E+02	2.4158E+03 ± 2.1663E+02	1.9438E+03 ± 3.0608E+02	2.3627E+03 ± 2.1384E+02
F21	3.8167E+02 ± 2.8112E+01	1.0482E+03 ± 6.6065E+01	1.0063E+03 ± 8.5782E+01	9.5068E+02 ± 8.5382E+01	9.9053E+02 ± 8.3543E+01	9.0450E+02 ± 8.0330E+01	1.1078E+03 ± 5.5020E+01
F22	8.1836E+03 ± 1.6714E+03	1.4294E+04 ± 6.1543E+02	1.3871E+04 ± 6.4471E+02	1.3396E+04 ± 9.0451E+02	1.4354E+04 ± 6.2315E+02	1.3605E+04 ± 9.5172E+02	1.4471E+04 ± 6.3943E+02
F23	7.6739E+02 ± 1.0023E+02	2.2335E+03 ± 2.9222E+02	1.6205E+03 ± 2.1258E+02	1.7558E+03 ± 2.1525E+02	1.9357E+03 ± 1.8703E+02	2.1187E+03 ± 2.5967E+02	2.1169E+03 ± 1.8790E+02
F24	1.1400E+03 ± 9.2832E+01	2.5457E+03 ± 1.5308E+02	1.8116E+03 ± 1.9521E+02	2.1285E+03 ± 1.5448E+02	2.3925E+03 ± 1.8420E+02	2.4575E+03 ± 2.4007E+02	2.5451E+03 ± 1.4147E+02
F25	1.6246E+02 ± 6.4573E+01	1.0850E+04 ± 2.5149E+03	5.8967E+03 ± 1.1903E+03	7.1637E+03 ± 2.0808E+03	7.0031E+03 ± 1.8160E+03	5.5501E+03 ± 1.4322E+03	9.4641E+03 ± 1.3399E+03
F26	4.3083E+03 ± 8.0860E+02	1.4873E+04 ± 1.9749E+03	1.2239E+04 ± 1.4169E+03	1.2425E+04 ± 1.6019E+03	1.4220E+04 ± 1.7318E+03	1.2204E+04 ± 1.4312E+03	1.6525E+04 ± 1.7186E+03
F27	1.2360E+03 ± 1.3711E+02	3.7646E+03 ± 4.9815E+02	2.1857E+03 ± 5.0103E+02	2.2711E+03 ± 3.8138E+02	3.0666E+03 ± 4.6450E+02	3.3675E+03 ± 5.6202E+02	3.5484E+03 ± 3.7847E+02
F28	9.8033E+02 ± 1.7181E+02	5.4994E+03 ± 1.6023E+03	3.7657E+03 ± 6.9821E+02	4.0631E+03 ± 8.0567E+02	4.5123E+03 ± 8.5003E+02	3.7043E+03 ± 7.1863E+02	6.2312E+03 ± 7.2382E+02
F29	7.8775E+03 ± 1.8429E+03	1.0282E+05 ± 1.2736E+05	1.0747E+04 ± 5.3105E+03	7.3736E+03 ± 3.1710E+03	2.7626E+04 ± 5.1174E+04	1.8902E+04 ± 1.6080E+04	1.6458E+05 ± 1.3575E+05
F30	3.2055E+07 ± 3.2796E+07	1.0645E+10 ± 3.3816E+09	3.5709E+09 ± 2.0299E+09	2.3983E+09 ± 1.4910E+09	5.6606E+09 ± 2.5262E+09	5.7796E+09 ± 2.2432E+09	1.2088E+10 ± 2.8058E+09

**Table 20:** Two-sided Wilcoxon signed-rank test results (α = 0.05) of SAWHALE against each competitor on the CEC 2017 problems, by function family. Each cell reports wins/ties/losses from SAWHALE’s perspective.

D	Family	AOA	PARROT	SINHCOSH	DGSWOA	EWOA	MHWOA
	Unimodal (F1–F3)	1/0/2	1/1/1	1/1/1	1/1/1	1/0/2	2/1/0
30	Multimodal (F4–F10)	7/0/0	7/0/0	7/0/0	7/0/0	7/0/0	7/0/0
	Hybrid (F11–F20)	9/0/1	7/2/1	7/2/1	8/2/0	4/4/2	9/1/0
	Composition (F21–F30)	9/1/0	9/0/1	9/0/1	9/0/1	9/0/1	9/1/0
	Unimodal (F1–F3)	2/1/0	2/0/1	1/1/1	2/0/1	1/0/2	3/0/0
50	Multimodal (F4–F10)	7/0/0	7/0/0	7/0/0	7/0/0	7/0/0	7/0/0
	Hybrid (F11–F20)	9/0/1	8/0/2	8/0/2	8/0/2	8/0/2	9/0/1
	Composition (F21–F30)	10/0/0	10/0/0	9/1/0	10/0/0	10/0/0	10/0/0

**Table 21:** Formulation characteristics of the employed constrained engineering design problems.

	Engineering Design Problems	*D*	*g_num_*	*h_num_*	*NFEs*
CONST1	Stepped Cantilever Beam Design	10	11	0	40,000
CONST2	Car Side Impact Problem	11	10	0	30,000
CONST3	Belleville Spring Design Problem	4	7	0	40,000
CONST4	Optimal Design of a Reactor	8	4	0	30,000
CONST5	Optimal Operation of an Alkylation Unit	7	14	0	40,000
CONST6	Optimal Design of a Refrigeration System	14	15	0	30,000
CONST7	Optimal Design of a Speed Reducer	7	11	0	40,000
CONST8	Optimal Design of a Pressure Vessel	4	4	0	30,000
CONST9	Minimum Weight of Tension/Compression Spring	3	4	0	40,000
CONST10	Optimal Design of a Welded Beam	4	7	0	30,000
CONST11	Optimal Design of a Hydrostatic Thrust Bearing	4	7	0	10,000
CONST12	Optimal Design of a Heat Exchanger	8	6	0	50,000

**Table 22:** Optimal results for constrained engineering design problems.

	CONST1	CONST2	CONST3	CONST4	CONST5	CONST6
	EQUIL	SAWHALE	SAWHALE	SAWHALE	SAWHALE	SAWHALE
*x_1_*	3.0000000	0.5000000	12.00999308	6.31473063	1695.91531	0.00100000
*x_2_*	60.000000	1.1163609	10.03046461	2.37494536	54.9196209	0.00100000
*x_3_*	3.1000000	0.5000000	0.2041433	0.66788149	3029.43867	0.00100000
*x_4_*	55.000000	1.3022049	0.2000000	0.59526366	90.2758675	0.00100000
*x_5_*	2.6000000	0.5000000	—	5.95842188	94.9991714	0.00100000
*x_6_*	50.000000	1.5000000	—	5.52885470	10.3208089	0.00100000
*x_7_*	2.2808873	0.5000000	—	1.05174645	153.532830	1.52400000
*x_8_*	45.617747	0.3450000	—	0.40303949	—	1.52400000
*x_9_*	1.7497572	0.3450000	—	—	—	4.99999277
*x_10_*	34.995142	−19.562359	—	—	—	2.00000001
*x_11_*	—	−0.0000786	—	—	—	0.00100000
*x_12_*	—	—	—	—	—	0.00100000
*x_13_*	—	—	—	—	—	0.00729331
*x_14_*	—	—	—	—	—	0.08755363
*g_1_*(*x*)	−0.0030914	−0.61758157	−0.00000004	−0.00004209	−0.0000061	−0.0000001
*g_2_*(*x*)	−1359.0492	−0.07832427	−0.00000193	−0.00000561	−0.0198962	−0.0000003
*g_3_*(*x*)	−153.84615	−0.09219170	−0.77970365	−0.00006730	−0.0000032	−7.5615924
*g_4_*(*x*)	−1203.4124	−0.02171317	−1.59585663	−0.00000554	−0.0199000	−0.9788564
*g_5_*(*x*)	−111.11111	−3.68949981	−0.00000692	—	−0.0094308	−0.0000131
*g_6_*(*x*)	−1.239E−10	−5.28782450	−1.97952847	—	−0.0000002	−0.0000253
*g_7_*(*x*)	0.0000000	−0.98685000	−0.19896584	—	−0.0000005	−0.9801997
*g_8_*(*x*)	−2.2580645	−1.4353E−10	—	—	−0.0232805	−0.9389367
*g_9_*(*x*)	−0.7692307	−0.63231455	—	—	−0.0000056	−0.9901000
*g_10_*(*x*)	−9.742E−10	−0.16675720	—	—	−0.5408674	−0.9807000
*g_11_*(*x*)	−0.0000013	—	—	—	−0.6122217	−0.9702000
*g_12_*(*x*)	—	—	—	—	−0.0178412	−0.9439999
*g_13_*(*x*)	—	—	—	—	−0.0310488	−0.5999994
*g_14_*(*x*)	—	—	—	—	−9.1409151	−0.0000032
*g_15_*(*x*)	—	—	—	—	—	−0.0000004
*f*(*x*)	64578.19447	22.84296920	1.97967523	3.95230523	1228.18924	0.03221358

— means not available for this entity.

**Table 23:** Best-performing algorithms for different engineering design problems.

	CONST7	CONST8	CONST9	CONST10	CONST11	CONST12
	SAWHALE, EQUIL	GRAD	SAWHALE	SAWHALE, GRAD, EQUIL	GRAD	SAWHALE
*x_1_*	3.50000000	0.7720000	0.05170854	0.20572964	5.95846769	304.48468260
*x_2_*	0.70000000	0.3816000	0.35718608	3.47048867	5.38641474	1464.8899579
*x_3_*	17.0000000	40.000000	11.2616170	9.03662391	2.33980573	5337.1232354
*x_4_*	7.30000000	204.49767	—	0.20572964	0.00000544	153.52076260
*x_5_*	7.80000000	—	—	—	—	286.51508740
*x_6_*	3.35021467	—	—	—	—	246.47867713
*x_7_*	5.00000000	—	—	—	—	267.00567331
*x_8_*	—	—	—	—	—	386.51508259
*g_1_*(*x*)	−0.07391528	−0.00007923	−0.00000364	0.00000000	−0.00000776	−0.00000003
*g_2_*(*x*)	−0.19799853	−0.00000397	−0.00000094	0.00000000	−0.00006147	−0.00000042
*g_3_*(*x*)	−0.49917225	−0.00000042	−4.05468613	0.00000000	−1.22273871	−0.00000001
*g_4_*(*x*)	−0.87685575	−35.5023256	−0.72740358	−3.43298377	−0.00034846	−0.00000149
*g_5_*(*x*)	−0.00000023	—	—	−0.08072964	−0.57205295	−0.00000037
*g_6_*(*x*)	−0.62366525	—	—	−0.23554032	−0.00082933	−0.00000005
*g_7_*(*x*)	−0.70250000	—	—	−0.00000078	−46.2364534	—
*g_8_*(*x*)	−0.00000049	—	—	—	—	—
*g_9_*(*x*)	−0.58333333	—	—	—	—	—
*g_10_*(*x*)	−0.05132575	—	—	—	—	—
*g_11_*(*x*)	−0.05128205	—	—	—	—	—
*f*(*x*)	2823.66247	5874.871899	0.01266530	1.7245275	19698.17767	7106.497876

— means not available for this entity.

**Table 24:** Description of the individual data sets employed in numerical experiments.

No	Dataset	No. of Features	No. of Instances
1	Tic-tac-toe	9	958
2	Breastcancer	9	699
3	HeartEW	13	270
4	Exactly	13	10
5	Exactly2	13	10
6	M-of-n	13	1000
7	Zoo	16	101
8	CongressEW	16	435
9	Vote	16	300
10	SpectEW	22	267
11	BreastEW	30	569
12	IonosphereEW	34	351
13	KrvskpEW	36	3196
14	WaveformEW	40	5000
15	SonarEW	60	208
16	Clean1	166	476
17	Clean2	166	6598
18	Semeion	256	1593

**Table 25:** Respective *p*-values from the Wilcoxon signed-rank test results for feature selection problems.

	WHALE	GWO	MOTH	MVO	EQUIL	GRAD
Tic-tac-toe	1.763E-04	7.942E-05	8.743E-05	9.732E-05	8.553E-04	2.172E-05
Breastcancer	2.772E-05	6.284E-05	6.592E-05	7.894E-05	1.824E-01	6.921E-04
HeartEW	8.641E-05	1.042E-03	2.421E-03	1.422E-02	7.621E-02	8.989E-02
Exactly	1.042E-05	8.833E-03	7.542E-03	5.823E-01	1.083E-01	1.112E-02
Exactly2	1.484E-06	7.924E-05	5.781E-03	1.842E-03	8.924E-03	4.733E-02
M-of-n	8.082E-08	1.782E-07	1.344E-06	7.035E-06	3.841E-04	1.762E-04
Zoo	1.298E-05	7.978E-06	5.867E-05	5.972E-05	1.926E-06	6.331E-05
CongressEW	7.921E-05	1.021E-04	9.826E-03	9.621E-03	9.827E-02	8.821E-04
Vote	6.789E-06	3.078E-03	3.721E-03	3.295E-03	2.978E-04	1.083E-03
SpectEW	9.256E-03	9.875E-03	1.027E-02	2.861E-02	1.982E-01	8.992E-02
BreastEW	8.721E-05	5.672E-04	1.782E-04	9.721E-03	8.721E-03	8.752E-03
IonosphereEW	1.826E-03	5.063E-03	6.927E-03	7.892E-03	8.633E-02	4.826E-04
KrvskpEW	1.762E-04	1.081E-03	9.652E-04	2.789E-03	8.984E-01	5.792E-01
WaveformEW	9.442E-04	7.894E-02	1.074E-03	1.002E-02	3.921E-02	9.869E-03
SonarEW	1.284E-04	6.762E-02	8.422E-04	9.725E-03	8.721E-03	9.671E-03
Clean1	8.762E-03	2.451E-02	1.826E-02	1.863E-02	2.134E-02	1.123E-02
Clean2	1.826E-05	7.927E-04	2.973E-05	1.029E-03	2.656E-02	2.821E-02
Semeion	1.781E-05	1.942E-04	6.821E-05	3.829E-05	2.866E-04	2.762E-04

## Data Availability

The original contributions presented in this study are included in the article. Further inquiries can be directed to the corresponding author.
